# A chromosome-scale reference genome of *Lobularia maritima*, an ornamental plant with high stress tolerance

**DOI:** 10.1038/s41438-020-00422-w

**Published:** 2020-12-01

**Authors:** Li Huang, Yazhen Ma, Jiebei Jiang, Ting Li, Wenjie Yang, Lei Zhang, Lei Wu, Landi Feng, Zhenxiang Xi, Xiaoting Xu, Jianquan Liu, Quanjun Hu

**Affiliations:** 1 Key Laboratory of Bio-Resource and Eco-Environment of Ministry of Education, College of Life Sciences, Sichuan University, 610065 Chengdu, China; 2 State Key Laboratory of Grassland Agro-Ecosystem, Institute of Innovation Ecology, Lanzhou University, Lanzhou, China

**Keywords:** Plant stress responses, Plant breeding, Genome

## Abstract

*Lobularia maritima* (L.) Desv. is an ornamental plant cultivated across the world. It belongs to the family Brassicaceae and can tolerate dry, poor and contaminated habitats. Here, we present a chromosome-scale, high-quality genome assembly of *L. maritima* based on integrated approaches combining Illumina short reads and Hi–C chromosome conformation data. The genome was assembled into 12 pseudochromosomes with a 197.70 Mb length, and it includes 25,813 protein-coding genes. Approximately 41.94% of the genome consists of repetitive sequences, with abundant long terminal repeat transposable elements. Comparative genomic analysis confirmed that *L. maritima* underwent a species-specific whole-genome duplication (WGD) event ~22.99 million years ago. We identified ~1900 species-specific genes, 25 expanded gene families, and 50 positively selected genes in *L. maritima*. Functional annotations of these genes indicated that they are mainly related to stress tolerance. These results provide new insights into the stress tolerance of *L. maritima*, and this genomic resource will be valuable for further genetic improvement of this important ornamental plant.

## Introduction

Whole-genome duplication (WGD), or polyploidy, has had a strong influence on the evolution of the tree of life, and it seems to have occurred in the evolutionary history of most plant species^1,2^, especially in angiosperms^3^. WGDs have been found in most angiosperm families with abundant species, including Brassicaceae, Poaceae, Asteraceae, Solanaceae, Fabaceae and Orchidaceae^4–11^. Previous studies suggested that WGDs can strengthen the adaptation of plants to environmental challenges^12^ because of genomic reorganization and novelties. Through subfunctionalization or reciprocal loss of duplicated genes in differentiated populations of an ancestral species, WGDs can also promote reproductive isolation and thus facilitate speciation^13^. Brassicaceae (also known as Cruciferae), a monophyletic group distributed worldwide, has been highly diversified by complicated WGD events and subsequent evolution, with ~350 genera and 4000 species^14,15^. It contains many important crops (e.g., cabbage, rapeseed and mustard) that have been domesticated for food, biofuels, and ornamentals^16^. The well-known model organism *Arabidopsis thaliana*, which is of paramount importance in studies of the development, gene expression and genome evolution of flowering plants, is also a member of this family^17,18^. Analyses of the *A. thaliana* genome have provided clear evidence that three ancient WGD events (γ, β and α), occurred in its evolutionary history. The oldest WGD event, the At-γ event, was related to the diversification of eudicots and perhaps all angiosperms^19–21^. The At-β event postdated the Brassicaceae–Caricaceae divergence ~70 million years ago (Mya)^22,23^. However, the At-α event was specific to the Brassicaceae family^19^, occurring ~40 Mya^24^. In addition, independent WGDs more recent than the Neogene may have promoted the colonization of harsh environments by Brassicaceae taxa by increasing their stress tolerance and conferring high adaptability^25,26^. However, detailed investigation of WGDs in numerous genera present in arid habitats is still badly needed^27,28^.

*Lobularia maritima* (L.) Desv., commonly known as sweet alyssum, is a perennial and diploid (2n = 24) herbaceous plant of the family Brassicaceae. This ornamental plant naturally occurs in the western Mediterranean region and has been widely cultivated since its domestication^29,30^. Its flowers range in color from pale violet to deep purple^31^. In addition to tolerating dry and poor habitats, *L. maritima* is recognized as a nickel hyperaccumulator that can remove heavy metals from contaminated soils^32^. As a facultative halophyte closely related to *Arabidopsis thaliana*, *L. maritima* seems to be an ideal model for revealing the molecular mechanisms underlying plant tolerance to drought and salt stress^33^. However, studies of *L. maritima* have focused mainly on its cultivation, management and rapid propagation in vitro^29^.

In this study, we report a chromosome-scale assembly of the *L. maritima* genome anchored on 12 pseudochromosomes. We further identified a recent *L. maritima*-specific WGD event that occurred after the Brassicaceae-specific At-α event using comparative and evolutionary analyses. We also revealed numerous genomic changes by which *L. maritima* has adapted to harsh habitats.

## Results

### Genome sequencing and assembly

**Fig. 1:**
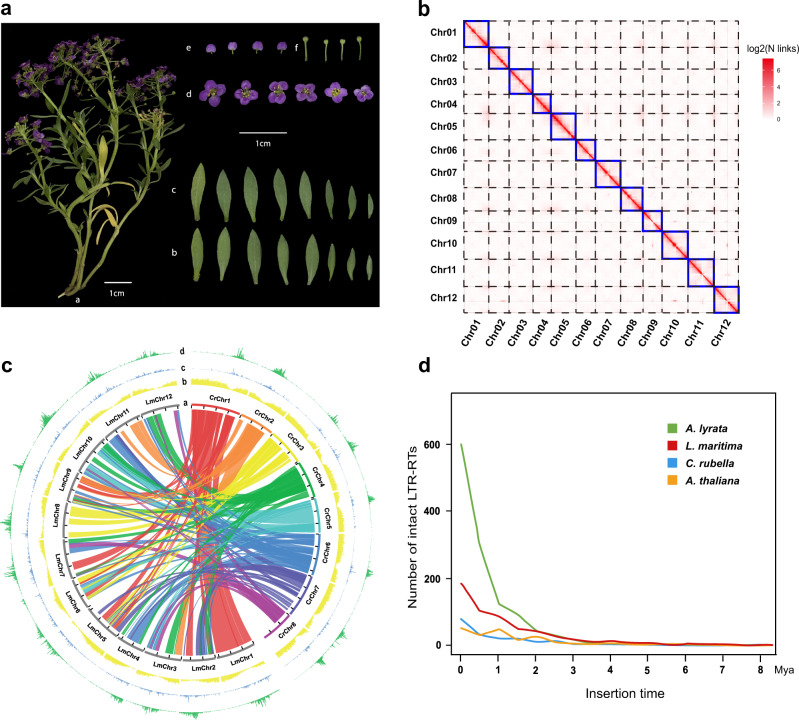
Morphological and genomic characteristics of *L. maritima*. **a** Morphological characteristics of *L. maritima*: schematic representations of (a) aerial parts; (b) abaxial surfaces of leaves; (c) adaxial surfaces of leaves; (d) flowers; (e) petals; and (f) a receptacle and pedicel. **b** Hi–C chromatin interaction map for the 12 pseudochromosomes of the *L. maritima* genome. **c** Genome comparison between *L. maritima* and *C. rubella* at the chromosome level: (a) syntenic relationships between the *L. maritima* and *C. rubella* genomes; (b) gene density (window size = 100 kb, nonoverlapping); (c) density distribution of *Copia* elements (window size = 100 kb, nonoverlapping); and (d) density distribution of *Gypsy* elements (window size = 100 kb, nonoverlapping). **d** Estimated insertion times of intact LTR retrotransposons

Samples for genome sequencing were obtained from an *L. maritima* seedling with purple flowers (Fig. 1a). We obtained 59.77 Gb of clean reads with various insert sizes and 22.31 Gb of Hi–C clean reads (~112.89-fold coverage) after Illumina sequencing and quality control (Supplementary Table 1). Two methods were employed to estimate the genome size of *L. maritima*. First, we determined the *L. maritima* genome size to be 225 Mb using flow cytometry with *A. thaliana* as the external control (Supplementary Fig. 1). Second, we used *k*-mer-based statistics^34^, and the genome size was calculated to be 264 Mb (Supplementary Fig. 2).

Based on the clean reads, a de novo genome was assembled with a 197.70 Mb length. We further anchored this genome on 12 pseudochromosomes (Fig. 1b and Table 1 and Supplementary Fig. 3). We then evaluated the completeness of this genome using BUSCO v4.1.2^35^ and found that 99% of the single-copy orthologs were intact (Supplementary Table 2), suggesting the high quality of the assembled genome.

**Table 1:** Assembly and annotation statistics of the *L. maritima* genome

Genome feature	Value
Estimated genome size (Mb)	264
Total scaffold number	27,734
Total length (bp)	197,688,650
Total length of chromosomes (bp)	174,586,151
Longest scaffold length (bp)	16,503,592
Scaffold L50	7
Scaffold N50 length (bp)	14,943,599
GC content (%)	36.02
Repeat content (%)	41.94
Number of predicted genes	25,813
Average coding sequence length (bp)	241
Average gene length (bp)	2431
Number of exons	140,984
Average number of exons per gene	5.46

### Genome annotation

To predict protein-coding sequences, we combined de novo and homology- and transcriptome-based methods. We predicted 25,813 complete protein-coding genes. Gene length and the number of exons of these protein-coding genes were 2431 base pairs (bp) and 5.46 exons, respectively, on average (Table 1). In our assembly, 97.99% (25,295 of 25,813) of the genes were annotated on 12 pseudochromosomes, and only 2.01% (518 of 25,813) were located on scaffolds. The Circos v0.69 (http://circos.ca) was used to visualize the collinearity blocks between *L. maritima* and *Capsella rubella*, gene density, *Copia* density, and *Gypsy* density on individual chromosomes (Fig. 1c). Among the 25,813 predicted genes, 81.30% and 95.71% had homologs in the Swiss-Prot^36^ and TrEMBL^36^ databases, respectively. Additionally, we annotated 95.15%, 80.65%, and 36.55% of the genes using the InterPro^37^, Gene Ontology (GO)^38^ and Kyoto Encyclopedia of Genes and Genomes (KEGG)^39^ databases, respectively (Supplementary Table 3). In addition, 41.94% (83 Mb) of the assembled *L. maritima* genome comprised repetitive sequences (Supplementary Table 4). Of these repetitive sequences, long terminal repeat (LTR) retrotransposons were the most frequent, spanning 14.24% of the assembled genome with 13.23% intact LTR retrotransposons. The other common repetitive sequences were DNA transposons (10.06%), Tandem Repeats (8.56%) and LINEs (5.65%) (Supplementary Tables 4 and 5). To analyze the evolutionary dynamics of these LTRs, we estimated their insertion dates in four related species (*A. thaliana*, *Arabidopsis lyrata*, *C. rubella* and *L. maritima*). The recent insertions in *A. lyrata* may have contributed to its relatively large genome size (207 Mb). Similarly, *L. maritima* had more recent insertions than *A. thaliana* and *C. rubella* (Fig. 1d and Supplementary Table 5). Diverse genetic changes can be caused by transposable elements (including LTR retrotransposons), which might have promoted lineage-specific diversification and adaptation^40^. This may partly contribute to the tolerance of *L. maritima* to arid habitats. However, the *L. maritima* genome contained a similar number of transcription factors (1799) as the other closely related Brassicaceae species (Supplementary Table 6, all transcription factor data for other species were downloaded from http://www.transcriptionfactor.org).

### Comparative genomic analyses and WGD analyses

**Fig. 2:**
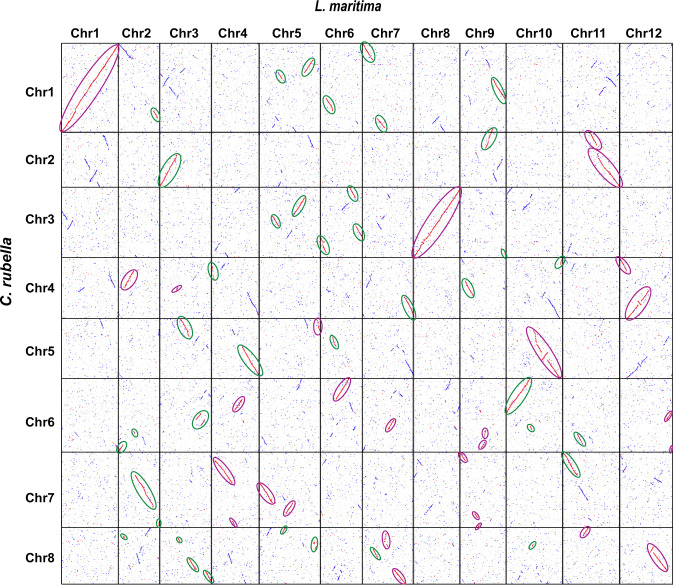
Dotplot comparing the *L. maritima* and *C. rubella* genomes. Collinear regions in the *L. maritima* genome. Regions from putative subgenomes are circled in purple and green, respectively

Using the ColinearScan v1.0.1^41^ program and MCScanX v1^42^ package, the protein sequences of *L. maritima* were compared to those of the diploid *C. rubella*, which has not been affected by a recent WGD event, to identify the collinear blocks in the genomes. The whole-genome alignments showed high collinearity and conservation, and several collinear regions almost completely spanned chromosomes of the two species (Fig. 2). It is worth noting that each chromosome or chromosomal region in *C. rubella* was represented on multiple independent chromosomes in the *L. maritima* genome after the Brassicaceae-specific At-α WGD event, suggesting that the *L. maritima* genome experienced a specific WGD event.

Furthermore, we determined the karyotype of *L. maritima* using previously reported methods^28,43^ (Supplementary Fig. 4) and recovered two sets of conserved genomic blocks^44,45^. However, the patterns of genomic blocks suggested that *L. maritima* experienced many postpolyploid diploidization events and a reduction in chromosome number. We also analyzed the gene retention rates of the two subgenomes in each genomic block with the *C. rubella* genome as the reference. The results showed that the two subgenomes retained similar numbers of genes (Supplementary Table 7). We also assessed the absence or presence of genome dominance by examining the expression levels of each pair of duplicated genes with high confidence. Based on RNA-seq data from flower, leaf, and stem tissues, we failed to find any evidence of biased expression in each genomic block between the two subgenomes (Supplementary Table 8). These results are largely consistent with the patterns of autopolyploids, which usually show a few instances of biased gene retention and no genome dominance.

### Recent WGD event in *L. maritima*

**Fig. 3:**
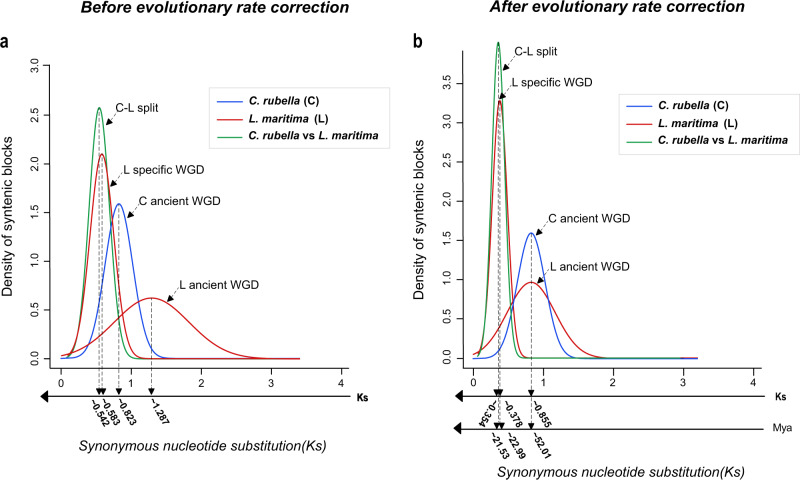
Evolutionary rate correction. **a** Distribution of uncorrected Ks values in syntenic blocks; **b** distribution of corrected Ks values in syntenic blocks and age estimates for the events

To identify possible WGD events, we calculated the Ks values between the collinear genes. The *L. maritima* collinear blocks produced two visible peaks, at 0.583 and 1.287 (Fig. 3a), representing two different WGD events. We then estimated the occurrence times of each WGD event based on the Ks values. However, dating ancestral events in plants can be influenced by divergent evolutionary rates^46^. Thus, by aligning the *L. maritima* peak with the corresponding location in the *C. rubella* Ks distribution, as in a previous report^46^, we performed evolutionary rate correction (Fig. 3b). After correction, the peaks of Ks for the two WGD events were 0.378 and 0.855, corresponding to 22.99 and 52.01 Mya, respectively (Fig. 3b). The results indicated an ancient WGD event shared with *C. rubella* and a recent species-specific WGD event in *L. maritima*. In addition, Ks estimation indicated that *C. rubella* and *L. maritima* diverged approximately 21.53 Mya (Fig. 3b). These findings were consistent with those of the synteny and collinearity analyses of *L. maritima* and *C. rubella* and suggested that *L. maritima* experienced a species-specific WGD event after sharing a WGD event with other Brassicaceae species.

### Phylogeny and divergence

**Fig. 4:**
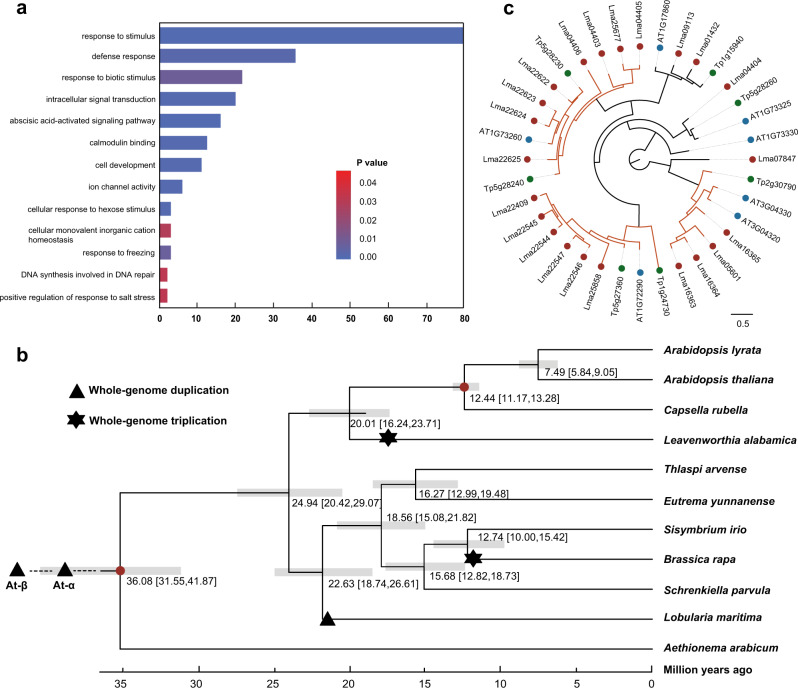
Evolutionary analyses of the *L. maritima* genome. **a** Gene Ontology enrichment of species-specific genes in *L. maritima* compared to 10 other species. **b** Estimation of divergence times between 11 species in the Brassicaceae family. The *A. thaliana* α and β duplication events were estimated to have occurred ~50 and ~60 million years ago, respectively^76^. **c** Phylogenetic tree of the *KTI* gene family constructed from sequences of *L. maritima*, *A. thaliana*, and *S. parvula*, indicating the expanded gene copies in *L. maritima*

We obtained the genome sequences of representative Brassicaceae species to clarify the genome evolution and divergence of *L. maritima*. Gene family clusters were defined based on the *L. maritima* protein-coding genes and the annotated gene sets of 10 published genomes (Supplementary Table 9) using OrthoFinder v2.3.12^47^. A total of 25,316 orthogroups were determined across the 11 species. Among these orthogroups, 1,986 were putative single-copy gene families, and 24,705 genes from *L. maritima* could be clustered into 16,821 orthogroups. In addition, we identified 1878 *L. maritima*-specific genes in these gene families. Functional annotations of these genes indicated that they were distinctly enriched in the GO terms “positive regulation of response to salt stress”, “abscisic acid-activated signaling pathway”, “response to freezing”, “response to stimulus”, and “response to biotic stimulus”, indicating that the genes retained after the WGD event may be relevant in the adaptation of *L. maritima* to multiple environmental stress factors (Fig. 4a and Supplementary Tables 10 and 11). For example, a homolog of these *L. maritima*-specific genes, *ABI4*, acts as both an activator and a repressor of gene expression and plays a critical role in phytohormone signaling pathways in plant development and biotic/abiotic stress responses^48^. Another homolog, *ABI1*, serves as a key repressor of the abscisic acid (ABA) signaling pathway and regulates diverse ABA responses to abiotic stress^49,50^. The species-specific calcium-dependent protein kinase (*CDPK*) genes recovered here (Supplementary Table 11) were also demonstrated to be involved in numerous aspects of plant growth and development, from sensing biotic and abiotic stress to mediating hormone-related development^51^.

To verify the phylogenetic position of *L. maritima*, we used the concatenated protein sequence alignment of the 1986 single-copy gene families in the 11-species phylogenetic analyses. The results confirmed that *L. maritima* belonged to Lineage II^11,52^ (Fig. 4b), consistent with its position in the chloroplast genome phylogeny reported previously^53^. In our analyses performed using MCMCtree^54^, *L. maritima* was estimated to have diverged from the other closely related species ~22.63 (18.74, 26.61) Mya (Fig. 4b).

### Expansion and contraction of gene families in *L. maritima*

Gene families with significantly expanded or contracted copy numbers are usually related to the adaptive divergence of one species from closely related species^55,56^. We compared the genomes of *L. maritima* and 10 other species, with *Aethionema arabicum* as the outgroup (Fig. 4b), to explore the expansion and contraction of the gene families in *L. maritima*. Twenty-five gene families, comprising 319 genes, were significantly expanded in *L. maritima* (*P* < 0.05). Functional annotation of these genes indicated that they were mainly enriched in “response to molecule of bacterial origin”, “response to insect”, “response to molecule of fungal origin”, “response to wounding” and “response to salt stress” (Supplementary Tables 12 and 13). For example, one of the expanded gene families, the *KTI* gene family, comprised versatile protease inhibitors related to defense against insect attack (Fig. 4c)^57^. In addition, the *HIPP* gene family, involved in stress responses^58^, was also greatly expanded in *L. maritima*.

### Positively selected genes in *L. maritima*

**Fig. 5:**
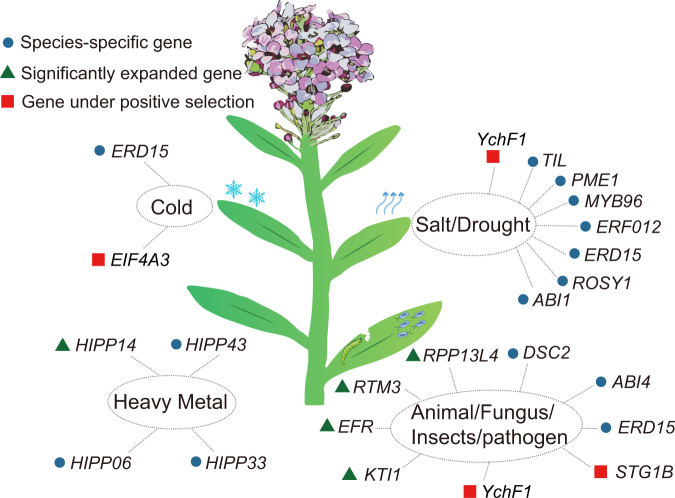
Genes in the *L. maritima* genome associated with various biotic and abiotic stimuli. Gene IDs for the gene names are listed below. *ABI1*: Lma13276; *ABI4*: Lma14310, Lma25376; *EFR*: Lma05693, Lma14720, Lma21740; *EIF4A3*: Lma15462; *ERD15*: Lma14373, Lma26266; *ERF012*: Lma17999; *HIPP06*: Lma19275; *HIPP14*: Lma12975; *HIPP33*: Lma25212; *HIPP43*: Lma16268; *KTI1*: Lma22625, Lma04403, Lma25677, Lma22622, Lma22621, Lma04406, Lma22624, Lma22623, Lma25676, Lma04405; *MYB96*: Lma13875; *STG1B*: Lma21436; *PME1*: Lma13269; *ROSY1*:Lma11854; *RPP13L4*: Lma09152, Lma03536, Lma21662, Lma03543, Lma09149, Lma21664, Lma09145; *RTM3*: Lma20357, Lma22531, Lma20352, Lma10871, Lma08006, Lma20353; *TIL*: Lma24858, Lma24859; *DSC2*: Lma14716 Lma17306; *YchF1*: Lma17801; Abbreviations: ABI ABA insensitive; EFR EF-TU receptor; EIF eukaryotic initiation factor; ERD early responsive to dehydration stress; ERF ethylene-responsive factor; HIPP heavy metal-associated isoprenylated plant protein; KTI Kunitz trypsin inhibitor; MYB myeloblastosis oncogene; PME pectin methyl esterase; ROSY interactor of synaptotagmin; RPP resistance to *P. pachyrhizi*; RTM restricted tobacco etch virus movement; TIL temperature-induced lipocalin; DSC2, desmocollin-2

Genes with signs of positive selection are usually regarded to be involved in the adaptive divergence of one species from closely related species^59^. We conducted positive selection analysis by using *L. maritima* as the foreground branch and five related Brassicaceae species (*Eutrema yunnanense*, *C. rubella*, *A. arabicum*, *A. lyrata*, and *Schrenkiella parvula*) as the background branches. We identified 10,581 single-copy orthologous gene families. To identify the genes that evolved in response to positive selection, we adopted the branch-site model in the PAML v4.9 package^54^. After false discovery rate (FDR) correction, we identified 50 genes that were possibly under positive selection. The functions of the significantly positively selected genes (PSGs) indicated that they were associated with stress tolerance and the survival of plants (Fig. 5 and Supplementary Table 14). For example, one of the genes was *SGT1B*, which was found to be involved in innate immunity and resistance in plants mediated by multiple R genes^60–63^. Another of the genes was *YchF1*, which is involved in salinity stress tolerance and disease resistance against bacterial pathogens^64^. Another of the genes, *EIF4A3*, is an important factor for abiotic stress adaptation, which can regulate plant resistance to abiotic stress partially by regulating the expression of acetoacetyl-CoA thiolase 2^65^.

## Discussion

*L. maritima* is an important ornamental plant in horticulture because of its colorful flowers and stress tolerance. In this study, by combining Illumina and Hi–C data, a chromosome-level high-quality *L. maritima* genome was assembled. The *L. maritima* genome was ~197.70 Mb in size, and 88.31% (174.59 Mb) of the sequences were assigned to 12 pseudochromosomes. We annotated 25,813 genes and found substantially more repetitive elements (especially intact LTR retrotransposons) in the *L. maritima* genome than in the genomes of other Brassicaceae species. In addition, most intact LTR retrotransposons expanded rapidly in the recent past. Such proliferation of LTR retrotransposons may have partly resulted in the increased genome size of *L. maritima*. Phylogenetic reconstructions showed that *L. maritima* diverged early as an independent branch of Brassicaceae Lineage II.

In the histories of many diverse eukaryotes, including *Danio rerio*^66^, *Saccharomyces cerevisiae*^67^, and *A. thaliana*^68–71^, WGDs have been discovered. Through large-scale phylogenomic analyses, ancient WGDs were found to occur in the common ancestors of both seed plants and angiosperms^4,9,71,72^. WGDs have played an essential role in angiosperm diversification and environmental adaptation^9^. Polyploids can tolerate high environmental stress, with present-day polyploids often appearing to occur at high frequencies in disturbed and harsh environments^73–75^. Under environmental stresses, polyploids may have been more successful because their changing environments created many opportunities to make use of the evolutionary benefits of WGDs^76^. The comparison of *L. maritima* and the diploid *C. rubella* indicated a recent WGD event that was specific to *L. maritima*, followed by extensive chromosomal rearrangements. Furthermore, we evaluated whether biased gene retention occurred after the WGD event. Two subgenomes retained a similar number of genes. However, neither subgenome showed genome dominance. This indicates that *L. maritima* might have undergone an autopolyploidization event. Analysis of the Ks values between the collinear genes suggested that the recent *L. maritima*-specific WGD event occurred ~22.99 Mya. The comparison of between-species Ks distributions indicated that the *L. maritima*-*C. rubella* divergence occurred ~21.53 Mya. Thus, this divergence and the aforementioned *L. maritima*-specific WGD event occurred at almost the same time. *L. maritima* and *C. rubella* belong to two major lineages, and it is highly likely that the divergence of the two major lineages and genus diversification of each lineage in Brassicaceae occurred radiatively at the same time. This rapid radiation was accompanied by polyploidy in a few of the genera. This is also consistent with the previous suggestion that further WGDs might have occurred in Brassicaceae since the Neogene, with radiative diversification, which further helped members of this family colonize arid habitats by increasing their stress tolerance^26^. As a result of the WGD event, species-specific genes and expanded gene families become further involved in responses to environmental stresses, for example, drought and pathogen attack, which might have facilitated the adaptation of *L*. maritima to harsh environments. In addition, the positively selected genes in *L. maritima* may have increased defense against fungal and bacterial attack. Thus, the species-specific WGD event may have promoted the adaptation of *L. maritima* to harsh environments, which is consistent with previous findings for numerous plants^76,77^. These genomic traits may also explain why *L. maritima* is a nickel hyperaccumulator^32^ and a halophyte with a high tolerance to salt stress^33^. Overall, whole-genome sequencing of *L. maritima* could elucidate the stress tolerance of this ornamental plant and be useful in future breeding programs.

## Materials and methods

### Materials and DNA/RNA extraction

The *L. maritima* seedling was cultivated in Jinjiang District, Chengdu City, Sichuan Province, China (N 30°34′21.86″, E 104°09′45.47″). We harvested fresh and healthy roots, stems, leaves and flowers and immediately froze them in liquid nitrogen. Before DNA/RNA extraction, we stored these tissues in a −80 °C freezer in the laboratory. To extract high-quality genomic DNA, the cetyl trimethylammonium bromide (CTAB)^78^ method was used. Additionally, we extracted total RNA from the flower, stem and leaf tissues using Qiagen RNeasy Plant Mini Kits.

### Library construction and sequencing

We randomly fragmented the purified genomic DNA using a focused ultrasonicator and obtained fragments of desired lengths by electrophoresing the DNA fragments in 0.8% General Purpose Agarose E-Gel. Then, we created Illumina libraries with large (2-, 5-, 10- and 20-kb) and small (350- and 500-bp) inserts using the purified DNA fragments. Based on the PE-150 protocol, the libraries were finally sequenced on an Illumina HiSeq 2000 platform. RNA libraries were constructed with a TruSeq RNA Library Preparation Kit v2 and sequenced on the same platform.

A Hi–C library was constructed using five main steps. First, we fixed the sample with formaldehyde and crosslinked DNA-DNA interactions that are bridged by proteins. Second, the crosslinked DNA was treated with the restriction endonuclease Hind III to produce sticky ends. Third, terminal DNA repair was used to introduce biotin-labeled bases in order to facilitate subsequent DNA purification and capture. Next, we ensured the location of the interacting DNA through cyclization of the end-repaired DNA and DNA fragments. Finally, we extracted and purified the DNA sample and then used Covaris S2 to shear the DNA sample. After A-tailing, pulldown, and adapter ligation, the DNA library was sequenced on an Illumina platform using the PE-150 protocol. We used HiCPro v2.8.1^79^ to remove duplicates and then assessed quality. After trimming low-quality reads and removing adapters, more than 22.31 Gb (~112.89-fold coverage) of clean data was generated. Then, all clean data were submitted to the 3D-DNA v180419 pipeline^80^.

### Genome assembly

Approximately 79.49 Gb of raw reads was generated by sequencing all six DNA libraries. These raw reads were filtered following a previous study^81^. We first used Trimmomatic v0.33^82^ to perform quality filtering of short reads. We then used the BFC error corrector^83^ followed by FastUniq v1.1^84^ to delete duplicates in the mate pair data. The resultant reads produced approximately 59.77 Gb of clean data (Supplementary Table 1).

We used Platanus v1.2.4^85^ software to perform *de novo* assembly of the *L. maritima* genome. Thereafter, using the 3D-DNA v180419 pipeline^80^, the draft assembly was scaffolded with the Hi–C clean reads. Using the Juicer v1.6.2 pipeline^86^, we aligned the Hi–C clean reads to the draft assembly genome. We then used Juicebox Assembly Tools^87^ to polish the results from the 3D-DNA v180419 pipeline. The Hi–C scaffolding was anchored on 12 pseudochromosomes. In total, 88.31% of the assembled sequences were related to the pseudochromosomes. In addition, we assessed the quality of the assembled genome using the BUSCO v4.1.2^35^ pipeline (database: embryophyta odb10, 2020-09-02, containing 1,614 BUSCO genes).

### Repeat element annotation

Repeat elements were identified with the RepeatMasker v4.0.7^88^ and RepeatModeler v1.0.11^89^ programs using the assembled *L. maritima* genome as the input. We also identified intact LTR retrotransposons by searching the *L. maritima* genome using LTRharvest v1.5.10^90^ and LTR_Finder v1.06^91^. We further combined these results using LTR_retriever v1.9^92^. We also estimated insertion time according to a substitution rate of 7 × 10^−9^/site/year.

### Gene prediction and annotation

To predict genes in the *L. maritima* genome, we first assembled transcripts using the *de novo* and genome-guided modes in Trinity v2.6.6^93^. Then, these transcripts were used to create transcript-based predictions with the PASA v2.1.0 pipeline^94^. We also carried out homolog predictions. In such predictions, the protein sequences of *A. thaliana*, *A. arabicum*, *A. lyrata*, *Eutrema yunnanense*, *Brassica rapa*, *Sisymbrium irio*, *C. rubella*, *Tarenaya hassleriana*, *Leavenworthia alabamica* and *Carica papaya* were mapped to the *L. maritima* genome using Exonerate v2.2.0 (https://www.ebi.ac.uk/about/vertebrate-genomics/software/exonerate). GlimmerHMM v3.0.4^95^ and Augustus v3.2.2^96^ were trained with genes from the PASA results and used for *de novo* gene prediction. We merged the gene models from the three sources using EVidenceModeler v1.1.1^97^. To annotate the functions of all predicted genes, we aligned the protein sequences of *L. maritima* to Swiss-Prot and TrEMBL^36^ using blastp and generated functional assignments based on the best hit. Protein domains were determined by searching against the InterPro^37^ database. In addition, Blast2GO v2.5^98^ was used to identify the Gene Ontology^38^ annotations and KEGG^39^ pathways using the KAAS server (https://www.genome.jp/kegg/kaas).

### Synteny and WGD

To construct syntenic blocks between *L. maritima* and *C. rubella*, all protein sequences of *L. maritima* were compared to protein sequences of *C. rubella*. The gene pairs with an *e*-value ≤ 1e-5 were further analyzed. We applied the ColinearScan v1.0.1^41^ program, which can effectively evaluate genomic blocks of collinear genes, and the MCScanX v1 package^42^ to find the syntenic blocks between the *C. rubella* and *L. maritima* genomes. Thereafter, we used these collinear gene pairs to construct a dotplot. Next, we used the script “add_ka_and_ks_to_collinearity.pl” in MCScanX to calculate the Ks values of the collinear orthologous gene pairs. We converted the Ks values to divergence times (T) based on *T* = *K*s/2*r*, where r is the neutral substitution rate (8.22 × 10^−9^). Finally, we performed evolutionary rate correction because of the inconsistent evolutionary rates among species. The evolutionary rate correction method was as reported by Wang et al.^46^. Briefly, under the assumption that the *C. rubella* peak appears at *k*_*C*_ and the *L. maritima* peak appears at *k*_*L*_, we can use the equation *r* = (*k*_*L*_ − *k*_*C*_)/*k*_*C*_ to describe the relative evolutionary rate of *L. maritima*. Then, rate correction was performed to discover the corrected rate *k*_*L* correction_ of *L. maritima* relative to *k*_*C*_: (1) For the Ks between duplicates in *L. maritima*, we defined the correction coefficient *W*_*L*_ as *k*_L correction_/*k*_*L*_ = *k*_*C*_/*k*_*L*_ = *W*_*L*_; thus, we obtained *k*_L correction_ = *k*_*C*_/*k*_*L*_ × *k*_*L*_ = 1/(1 + r) × *k*_*L*_ and *W*_*L*_ = 1/(1 + r). (2) For the Ks between homologous genes from *C. rubella* and *L. maritima*, if the peak was located at *k*_*L-C*_, supposing the correction coefficient *W*_*L*_ in *L. maritima*, we then calculated a corrected evolutionary rate *k*_L-C-correction_ = *W*_*L*_ × k_*L-C*._

### Phylogeny and divergence

The genomes of *L. maritima* and 10 other species (*A. arabicum*, *B. rapa*, *L. alabamica*, *E. yunnanense*, *S. irio*, *A. thaliana*, *A. lyrata*, *C. rubella*, *S. parvula* and *Thlaspi arvense*) were selected to generate clusters of gene families. We retained only the longest protein sequence. We removed redundant sequences based on alternative splicing variations. Using OrthoFinder v2.3.12^47^, we obtained orthologous gene families. Protein sequences from 1986 single-copy gene families were used to construct a phylogenetic tree. MAFFT v7.313^99^ software was used for sequence alignment of each single-copy gene family with default settings. A phylogenetic tree was built using RAxML v8.0.0^100^ under the PROTGAMMALGX model, and divergence times were calculated using the MCMCTree program of the PAML v4.9 package^54^. The calibration information for MCMCTree was extracted based on the TimeTree database^101^ (http://www.time.org/).

### Gene family expansion and contraction

Based on the dated phylogeny, we determined the expansions and contractions of orthologous gene families in the 11 Brassicaceae species (*A. arabicum*, *B. rapa*, *L. alabamica*, *E. yunnanense*, *S. irio*, *T. arvense, C. rubella*, *A. thaliana*, *A. lyrata*, *S. parvula*, and *L. maritima*) by using the CAFÉ v4.2^102^ program. Genes in significantly expanded families were then used for Gene Ontology enrichment analysis.

### Genes under positive selection

We selected six genomes, i.e., those of *A. arabicum*, *A. lyrata*, *C. rubella*, *E. yunnanense*, *S. parvula* and *L. maritima*, to identify orthologs for analyzing positive selection. First, Proteinortho v6.0.21^103^ was used to detect orthologs among the six genomes. Next, we used the PosiGene v0.1^104^ pipeline for genome-wide detection of the genes with positive selection and specified the *L. maritima* clade as the foreground branch. Finally, PSGs were identified based on an FDR-corrected *P* value < 0.05.

## Supplementary information

Supplementary figure 1-4

Supplementary table 1-10,12-14

Supplementary table 11

## Data Availability

Raw Illumina-short reads and Hi–C reads used for de novo whole-genome assembly have been deposited in the National Center for Biotechnology Information (NCBI) Sequence Read Archive database under accession number PRJNA630530. The genome and related annotation data have been deposited in the National Genomics Data Center (PRJCA002888).
