# Supplementary material for: A chromosome-scale reference genome of Lobularia maritima, an ornamental plant with high stress tolerance
Source: Hortic Res. 2020 Dec 1;7:197. doi: 10.1038/s41438-020-00422-w (PMC7705659; doi:10.1038/s41438-020-00422-w)
Supplement: Supplementary file 1 — Supplementary figure 1-4 [file 41438_2020_422_MOESM1_ESM.docx]

**Supplementary Figures**


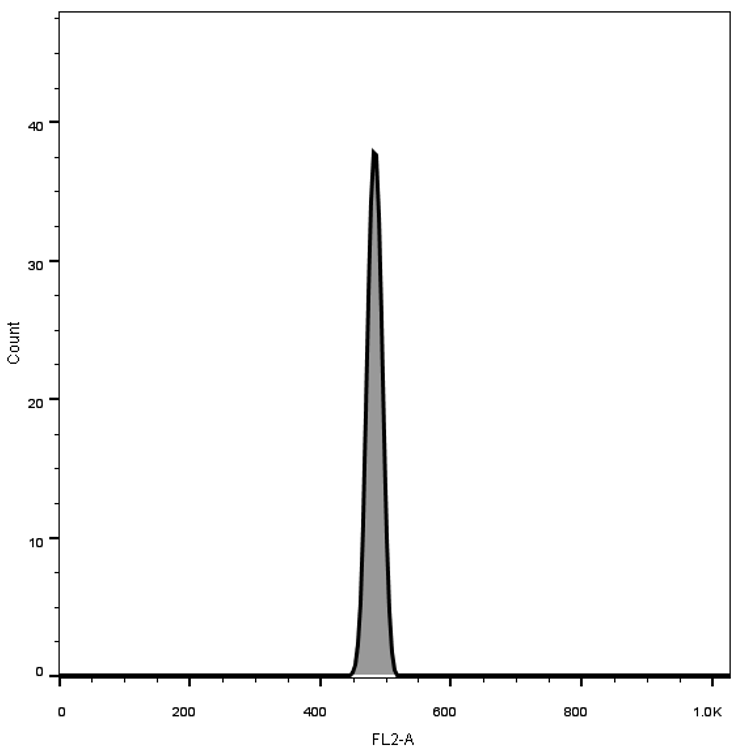


Supplementary Figure 1. The flow cytometry was used to estimate *L. maritima* genome size. The genome size was estimated to be 225 Mb.


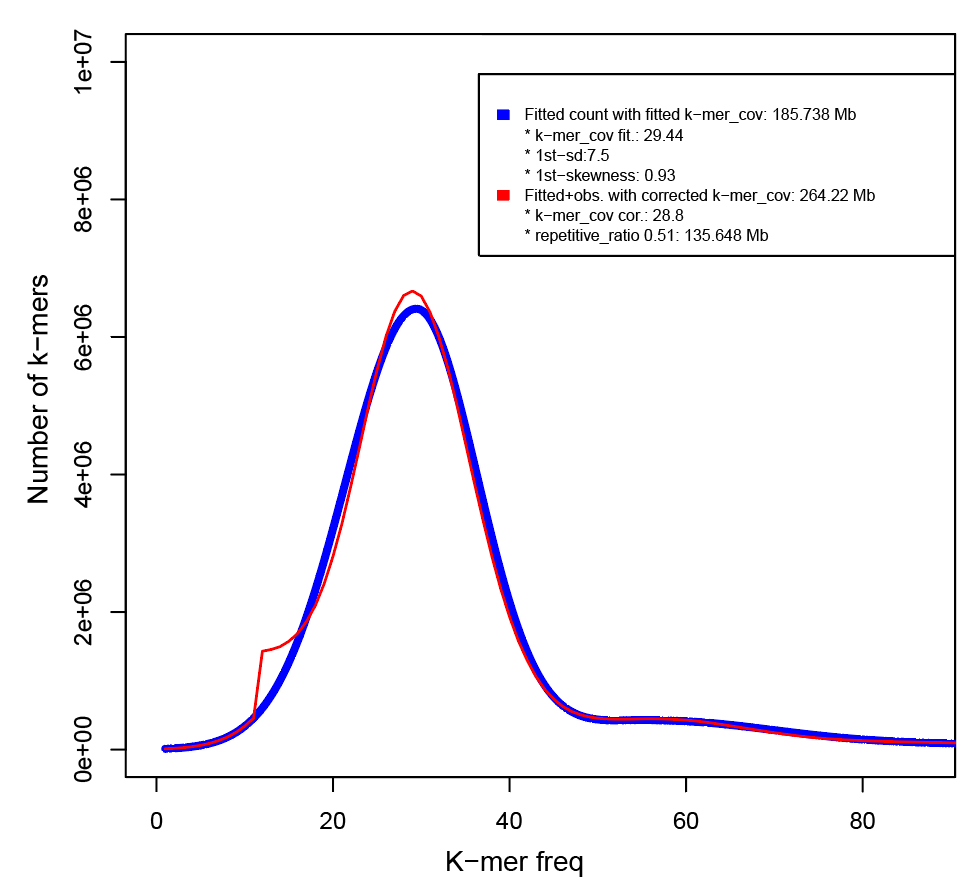


Supplementary Figure 2. The *K*-mer analysis used to estimate *L. maritima* genome size. The frequency of 17-mers were shown representing 17 bp sequences within reads (after filtering) from the clean reads of short-insert size libraries (350bp). The genome size of *L. maritima* was estimated to be 264 Mb.

.


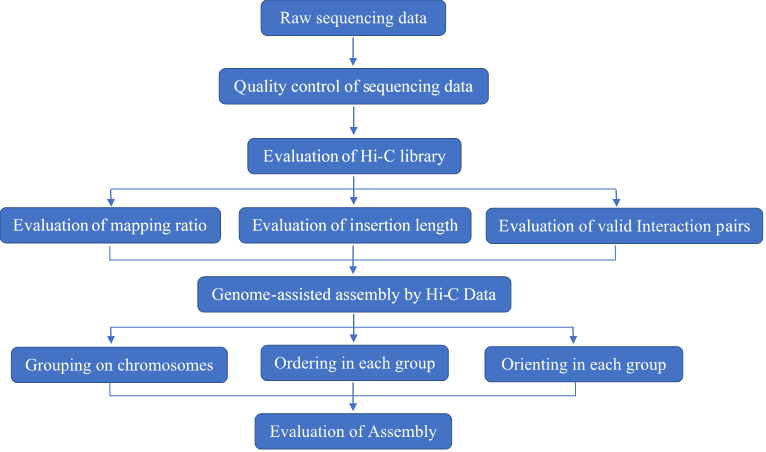


Supplementary Figure 3. The pipeline for Hi-C directed chromosome assembly in this study. After quality assessment of Hi-C library, those valid interactions were used for genome assembly.


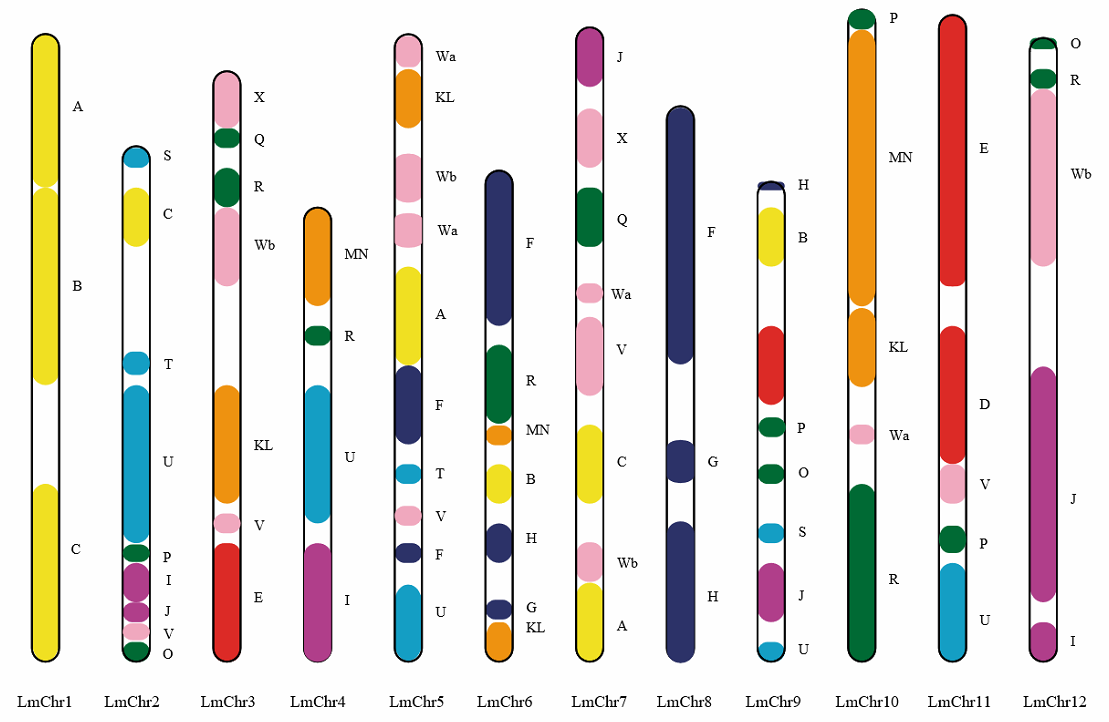


Supplementary Figure 4. The digital karyotype of *L. maritima*. Distribution of genic regions that are homologous to *Arabidopsis thaliana* were coloured according to previously defined genomic blocks by (Lysak and Mandakova, 2016).
