# Supplementary material for: A chromosome-scale reference genome of Lobularia maritima, an ornamental plant with high stress tolerance
Source: Hortic Res. 2020 Dec 1;7:197. doi: 10.1038/s41438-020-00422-w (PMC7705659; doi:10.1038/s41438-020-00422-w)
Supplement: Supplementary file 2 — Supplementary table 1-10,12-14 [file 41438_2020_422_MOESM2_ESM.docx]

**Supplementary Table 1. Overview of sequencing data in *Lobularia maritima*.**

| **Illumina Hiseq** | **Library ID** | **Insert size (bp)** | **Read length (bp)** | **Raw sequencing data** | | | **Clean sequencing data** | | | |
| --- | --- | --- | --- | --- | --- | --- | --- | --- | --- | --- |
|  |  |  |  | **Raw paired reads** | **Data (bp)** | **Sequencing depth (X)** | **Clean paired reads** | **Data (bp)** | **Clean_Data/Raw_Data (%)** | **Sequencing depth (X)** |
|  | DES00849_L2 | 350 | 150 | 67,307,382 | 20,192,214,600 | 102.14 | 58,330,606 | 17,499,181,800 | 86.66 | 88.52 |
|  | DES00850_L3 | 500 | 150 | 36,229,472 | 10,868,841,600 | 54.98 | 34,333,021 | 10,299,906,300 | 94.77 | 52.10 |
|  | DEL00658_L3 | 2000 | 150 | 41,731,746 | 12,519,523,800 | 63.33 | 35,534,906 | 10,660,471,800 | 85.15 | 53.93 |
|  | DEL00659_L8 | 5000 | 150 | 35,184,893 | 10,555,467,900 | 53.39 | 28,711,445 | 8,613,433,500 | 81.60 | 43.57 |
|  | DEL00660_L3 | 10000 | 150 | 40,926,609 | 12,277,982,700 | 62.11 | 24,962,630 | 7,488,789,000 | 60.99 | 37.88 |
|  | DEL00661_L3 | 20000 | 150 | 43,603,206 | 13,080,961,800 | 66.17 | 17,372,883 | 5,211,864,900 | 39.84 | 26.36 |
|  |  | Total |  | 264,983,308 | 79,494,992,400 | 402.12 | 199,245,491 | 59,773,647,300 | 449.02 | 302.36 |

| **Hi-C** | **Library-ID** | **Read length (bp)** | **Read Pairs Number** | **Base Number (bp)** | **GC Content（%）** | **%≥Q30** | **Sequencing depth (X)** |
| --- | --- | --- | --- | --- | --- | --- | --- |
|  | Sweetalyssum_G968-H02 | 150 | 74,522,600 | 22,317,085,726 | 38.68 | 90.87 | 112.89 |

**Note: Assuming the genome size of *Lobularia maritima* is 197.70 Mb.**

**Supplementary Table 2. Quality assessment of the assembled genome of *L. maritima* using BUSCOs.**

| **Type** | **Number** | **Percent (%)** |
| --- | --- | --- |
| Complete BUSCOs (C) | 1,597 | 99 |
| Complete and single-copy BUSCOs (S) | 1,531 | 94.9 |
| Complete and duplicated BUSCOs (D) | 66 | 4.1 |
| Fragmented BUSCOs (F) | 11 | 0.7 |
| Missing BUSCOs (M) | 6 | 0.3 |
| Total BUSCO groups searched | 1,614 | 100 |

**Supplementary Table 3. Summary of the functional annotation in *L. maritima* genome.**

| **Annotation database** | **Annotated number** | **Percentage (%)** |
| --- | --- | --- |
| Swiss-prot | 20,987 | 81.30 |
| TrEMBL | 24,706 | 95.71 |
| InterPro | 24,561 | 95.15 |
| GO | 20,817 | 80.65 |
| KEGG Pathway | 9,435 | 36.55 |
| Total | 25,381 | 98.33 |

**Supplementary Table 4. Repeat content within the genome of *L. maritima*.**

| **Type** | **Repeat size (bp)** | **% of genome** |
| --- | --- | --- |
| DNA | 19,880,518 | 10.06 |
| LTR | 28,153,423 | 14.24 |
| nonLTR | 67,229 | 0.03 |
| SINE | 813,160 | 0.41 |
| LINE | 11,160,649 | 5.65 |
| TRF | 16,919,928 | 8.56 |
| Simple repeats | 1,654,255 | 0.84 |
| Low complexity | 541,426 | 0.27 |
| Other | 2,202,589 | 1.11 |
| Unknown | 1,524,398 | 0.77 |
| Total | 82,917,575 | 41.94 |

**Supplementary Table 5. *L. maritima* and other three species intact LTR-RTs content.**

| **Species** | **LTR size (bp)** | **Genome size (bp)** | **% of genome** |
| --- | --- | --- | --- |
| *A. thaliana* | 7,863,937 | 119,146,348 | 6.60 |
| *C. rubella* | 7,563,566 | 134,110,326 | 5.64 |
| *A. lyrata* | 32,337,407 | 206,667,935 | 15.49 |
| *L. maritima* | 26,151,770 | 197,688,650 | 13.23 |

**Supplementary Table 6. Statistics of transcription factors (TFs) in *L. maritima* genome.**

| **TFs** | ***A. thaliana*** | ***C. rubella*** | ***A. lyrata*** | ***L. maritima*** | ***S. irio*** | ***A. arabicum*** | ***E. salsugineum*** |
| --- | --- | --- | --- | --- | --- | --- | --- |
| AP2 | 30 | 18 | 20 | 21 | 16 | 20 | 17 |
| ARF | 37 | 21 | 20 | 16 | 16 | 15 | 15 |
| ARR-B | 21 | 17 | 12 | 11 | 13 | 11 | 8 |
| B3 | 77 | 69 | 81 | 66 | 63 | 58 | 57 |
| BBR-BPC | 17 | 9 | 8 | 5 | 6 | 6 | 7 |
| BES1 | 14 | 10 | 9 | 7 | 8 | 9 | 8 |
| C2H2 | 116 | 108 | 104 | 103 | 98 | 70 | 89 |
| C3H | 66 | 60 | 54 | 54 | 53 | 40 | 49 |
| CAMTA | 10 | 7 | 6 | 7 | 6 | 6 | 6 |
| CO-like | 22 | 16 | 17 | 19 | 14 | 13 | 13 |
| CPP | 9 | 8 | 7 | 10 | 7 | 10 | 7 |
| DBB | 14 | 8 | 7 | 9 | 8 | 6 | 8 |
| Dof | 47 | 39 | 34 | 45 | 35 | 31 | 39 |
| E2F/DP | 16 | 8 | 7 | 9 | 9 | 7 | 8 |
| EIL | 6 | 6 | 6 | 8 | 7 | 7 | 7 |
| ERF | 139 | 126 | 124 | 140 | 123 | 102 | 109 |
| FAR1 | 26 | 22 | 5 | 16 | 19 | 15 | 15 |
| G2-like | 64 | 55 | 54 | 51 | 46 | 44 | 42 |
| GATA | 41 | 35 | 28 | 30 | 28 | 23 | 30 |
| GRAS | 37 | 39 | 37 | 37 | 34 | 30 | 36 |
| GRF | 9 | 8 | 9 | 11 | 9 | 7 | 9 |
| GeBP | 23 | 19 | 22 | 15 | 30 | 7 | 14 |
| HB-PHD | 3 | 4 | 2 | 4 | 2 | 2 | 2 |
| HB-other | 11 | 8 | 8 | 12 | 10 | 7 | 7 |
| HD-ZIP | 58 | 53 | 35 | 52 | 51 | 41 | 48 |
| HRT-like | 2 | 3 | 2 | 1 | 2 | 3 | 2 |
| HSF | 25 | 31 | 38 | 21 | 27 | 22 | 22 |
| LBD | 50 | 47 | 47 | 46 | 40 | 30 | 35 |
| LFY | 1 | 1 | 1 | 1 | 1 | 1 | 1 |
| LSD | 12 | 4 | 3 | 5 | 4 | 4 | 4 |
| M-type_MADSDS | 70 | 87 | 40 | 45 | 65 | 36 | 41 |
| MIKC_MADSDS | 76 | 43 | 39 | 44 | 35 | 26 | 31 |
| MYB | 168 | 157 | 149 | 149 | 139 | 117 | 129 |
| MYB_relatedd | 97 | 73 | 64 | 69 | 63 | 53 | 57 |
| NAC | 138 | 119 | 122 | 103 | 121 | 85 | 115 |
| NF-X1 | 2 | 2 | 3 | 3 | 2 | 2 | 3 |
| NF-YA | 21 | 12 | 12 | 12 | 10 | 7 | 9 |
| NF-YB | 27 | 12 | 16 | 17 | 12 | 13 | 13 |
| NF-YC | 21 | 14 | 16 | 16 | 14 | 7 | 13 |
| NZZ/SPL | 1 | 1 | 1 | 2 | 1 | 1 | 1 |
| Nin-like | 17 | 18 | 16 | 19 | 14 | 13 | 14 |
| RAV | 7 | 5 | 6 | 6 | 6 | 4 | 8 |
| S1Fa-like | 4 | 3 | 3 | 4 | 3 | 1 | 3 |
| SAP | 1 | 2 | 1 | 1 | 1 | 1 | 1 |
| SBP | 30 | 19 | 15 | 16 | 16 | 13 | 16 |
| SRS | 16 | 9 | 10 | 10 | 9 | 11 | 8 |
| STAT | 4 | 3 | 3 | 1 | 2 | 2 | 2 |
| TALE | 33 | 24 | 21 | 22 | 17 | 15 | 20 |
| TCP | 33 | 27 | 25 | 24 | 24 | 21 | 22 |
| Trihelix | 34 | 34 | 30 | 34 | 28 | 23 | 28 |
| VOZ | 3 | 2 | 3 | 2 | 2 | 2 | 2 |
| WOX | 18 | 15 | 16 | 16 | 16 | 13 | 15 |
| WRKY | 90 | 78 | 79 | 84 | 67 | 58 | 64 |
| Whirly | 4 | 4 | 4 | 3 | 3 | 2 | 2 |
| YABBY | 8 | 7 | 6 | 8 | 7 | 5 | 6 |
| ZF-HD | 18 | 18 | 17 | 20 | 15 | 15 | 16 |
| bHLH | 225 | 163 | 155 | 157 | 156 | 125 | 140 |
| bZIP | 127 | 88 | 80 | 80 | 77 | 53 | 68 |
| total | 2,296 | 1,898 | 1,759 | 1,799 | 1,710 | 1,371 | 1,561 |

**Supplementary Table 7. *L. maritima* gene retention rates in subgenomes with *C. rubella* as the reference genome.**

| **Genomic blocks** | **Gene Number in *C. rubella*** | **Retained gene number(L-A)** | **L-A/Gene Number** | **Retained gene number(L-B)** | **L-B/Gene Number** | **L A-B difference** |
| --- | --- | --- | --- | --- | --- | --- |
| A | 1936 | 1048 | 0.5413 | 788 | 0.4070 | 0.1343 |
| B | 1471 | 689 | 0.4684 | 689 | 0.4684 | 0.0000 |
| C | 1071 | 559 | 0.5219 | 442 | 0.4127 | 0.1092 |
| D | 810 | 330 | 0.4074 | 218 | 0.2691 | 0.1383 |
| E | 1788 | 930 | 0.5201 | 681 | 0.3809 | 0.1393 |
| F | 2583 | 1397 | 0.5408 | 1291 | 0.4998 | 0.0410 |
| G | 72 | 37 | 0.5139 | 32 | 0.4444 | 0.0694 |
| H | 812 | 416 | 0.5123 | 202 | 0.2488 | 0.2635 |
| I | 1223 | 524 | 0.4285 | 102 | 0.0834 | 0.3451 |
| J | 1864 | 1047 | 0.5617 | 757 | 0.4061 | 0.1556 |
| KL | 956 | 220 | 0.2301 | 358 | 0.3745 | -0.1444 |
| MN | 2139 | 1036 | 0.4843 | 855 | 0.3997 | 0.0846 |
| O | 520 | 223 | 0.4288 | 181 | 0.3481 | 0.0808 |
| P | 562 | 49 | 0.0872 | 240 | 0.4270 | -0.3399 |
| Q | 556 | 273 | 0.4910 | 152 | 0.2734 | 0.2176 |
| R | 2077 | 861 | 0.4145 | 1173 | 0.5648 | -0.1502 |
| S | 741 | 194 | 0.2618 | 56 | 0.756 | 0.1862 |
| T | 482 | 251 | 0.5207 | 77 | 0.1598 | 0.3610 |
| U | 2528 | 1409 | 0.5574 | 1355 | 0.5360 | 0.0214 |
| V | 721 | 298 | 0.4133 | 276 | 0.3828 | 0.0305 |
| Wa | 201 | 65 | 0.3234 | 78 | 0.3881 | -0.0647 |
| Wb | 1219 | 687 | 0.5636 | 538 | 0.4413 | 0.1222 |
| X | 711 | 414 | 0.5823 | 306 | 0.4304 | 0.1519 |

**Supplementary Table 8. Gene expression of high-confidence homoeologous genes pairs in the two subgenomes of *L. maritima.***

| **Genomic blocks** | **Subgenome A dominance^*^** | | | **Subgenome B dominance^*^** | | | **< 2-fold change** | | | **Low expression (<3 TPM)** | | |
| --- | --- | --- | --- | --- | --- | --- | --- | --- | --- | --- | --- | --- |
|  | Flower | Leaf | Stem | Flower | Leaf | Stem | Flower | Leaf | Stem | Flower | Leaf | Stem |
| A | 34 | 26 | 28 | 35 | 29 | 31 | 142 | 120 | 130 | 111 | 133 | 123 |
| B | 29 | 25 | 31 | 25 | 19 | 20 | 92 | 83 | 89 | 80 | 89 | 83 |
| C | 5 | 6 | 6 | 10 | 9 | 7 | 34 | 27 | 28 | 35 | 42 | 41 |
| D | 6 | 5 | 5 | 1 | 3 | 2 | 12 | 12 | 12 | 19 | 19 | 19q |
| E | 20 | 20 | 22 | 24 | 26 | 19 | 113 | 93 | 102 | 99 | 119 | 110 |
| F | 104 | 62 | 76 | 47 | 45 | 64 | 279 | 224 | 250 | 157 | 212 | 186 |
| G | 0 | 0 | 0 | 0 | 0 | 0 | 0 | 0 | 0 | 0 | 0 | 0 |
| H | 3 | 7 | 5 | 4 | 5 | 4 | 23 | 23 | 25 | 18 | 18 | 16 |
| I | 1 | 0 | 0 | 2 | 1 | 0 | 5 | 5 | 5 | 7 | 7 | 7 |
| J | 33 | 18 | 26 | 24 | 20 | 28 | 97 | 65 | 90 | 113 | 145 | 120 |
| KL | 0 | 0 | 0 | 3 | 0 | 1 | 4 | 1 | 2 | 5 | 8 | 7 |
| MN | 35 | 39 | 40 | 32 | 36 | 34 | 151 | 131 | 148 | 119 | 139 | 122 |
| O | 8 | 7 | 8 | 6 | 2 | 6 | 27 | 19 | 24 | 18 | 26 | 21 |
| P | 0 | 0 | 0 | 0 | 0 | 0 | 0 | 0 | 0 | 0 | 0 | 0 |
| Q | 7 | 5 | 3 | 7 | 2 | 5 | 22 | 21 | 21 | 20 | 21 | 21 |
| R | 55 | 41 |  | 43 | 31 |  | 217 | 171 |  | 172 | 218 |  |
| S | 0 | 0 | 0 | 0 | 0 | 0 | 0 | 0 | 0 | 0 | 0 | 0 |
| T | 0 | 0 | 1 | 1 | 1 | 1 | 2 | 2 | 3 | 3 | 3 | 2 |
| U | 38 | 30 | 37 | 30 | 28 | 31 | 141 | 123 | 133 | 156 | 174 | 164 |
| V | 7 | 6 | 6 | 5 | 6 | 6 | 23 | 24 | 25 | 24 | 23 | 22 |
| Wa | 0 | 0 | 0 | 0 | 0 | 0 | 0 | 0 | 0 | 0 | 0 | 0 |
| Wb | 16 | 18 | 16 | 15 | 11 | 20 | 71 | 60 | 74 | 58 | 69 | 55 |
| X | 24 | 22 | 21 | 21 | 14 | 21 | 77 | 65 | 79 | 55 | 67 | 53 |

^*^Subgenome A dominance is the number genes of TPM_L-A_/TPM_L-B_>2 fold change.

^*^Subgenome B dominance is the number genes of TPM_L-B_/ TPM_L-A_>2 fold change.

**Supplementary Table 9. Information for species used for reconstructing the phylogenetic tree.**

| **Species name** | **Version** | **Gene number** | **Data source/ GenBank accession number** |
| --- | --- | --- | --- |
| *A. arabicum* | V3.0 | 21,650 | https://genomevolution.org/coge/SearchResults.pl?s=Aethionema&p=genome |
| *A. lyrata* | v1.0 | 30,924 | https://phytozome.jgi.doe.gov/pz/portal.html#!bulk?org=Org_Alyrata |
| *A. thaliana* | Araport11 | 27,621 | https://phytozome.jgi.doe.gov/pz/portal.html#!bulk?org=Org_Athaliana_er |
| *B. rapa* | V3.0 | 46,221 | http://brassicadb.org/brad/datasets/pub/Genomes/Brassica_rapa/V3.0/ |
| *C. rubella* | v1.1 | 27,682 | https://phytozome.jgi.doe.gov/pz/portal.html#!bulk?org=Org_Crubella |
| *E. yunnanense* | v1.0 | 28,401 | NCBI:PKML00000000 |
| *L. alabamica* | v1.0 | 30,343 | http://mustang.biol.mcgill.ca:8885/download |
| *S. irio* | v1.0 | 26,959 | http://mustang.biol.mcgill.ca:8885/downloads.html |
| *T. arvense* | v1.0 | 38,735 | https://genome.jgi.doe.gov/portal/Phytozome/download |
| *S. parvula* | v2.0 | 25,185 | http://thellungiella.org/data/ |

**Supplementary Table 10. GO enrichment of species-specific genes in *L. maritima* compared to 10 other species.**

| **GO terms** | **Function** | **Annotated** | **Significant** | **P-value** |
| --- | --- | --- | --- | --- |
| GO:0050896 | response to stimulus | 4873 | 80 | 4.50E-07 |
| GO:0044699 | single-organism process | 9024 | 214 | 7.00E-06 |
| GO:0009738 | abscisic acid-activated signaling pathway | 213 | 16 | 1.90E-05 |
| GO:0018105 | peptidyl-serine phosphorylation | 67 | 8 | 0.00039 |
| GO:0030048 | actin filament-based movement | 17 | 4 | 0.0009 |
| GO:0007021 | tubulin complex assembly | 10 | 3 | 0.00197 |
| GO:0006768 | biotin metabolic process | 11 | 3 | 0.00265 |
| GO:0048468 | cell development | 266 | 11 | 0.00343 |
| GO:0071331 | cellular response to hexose stimulus | 12 | 3 | 0.00346 |
| GO:0009987 | cellular process | 11349 | 274 | 0.0038 |
| GO:0006952 | defense response | 987 | 36 | 0.00438 |
| GO:0032784 | regulation of DNA-templated transcription, elongation | 13 | 3 | 0.00441 |
| GO:0009554 | megasporogenesis | 14 | 3 | 0.00551 |
| GO:0006625 | protein targeting to peroxisome | 14 | 3 | 0.00551 |
| GO:0052386 | cell wall thickening | 41 | 6 | 0.00663 |
| GO:0032880 | regulation of protein localization | 21 | 3 | 0.00671 |
| GO:0050826 | response to freezing | 15 | 3 | 0.00675 |
| GO:0035556 | intracellular signal transduction | 531 | 20 | 0.00798 |
| GO:0007623 | circadian rhythm | 111 | 8 | 0.00971 |
| GO:0046777 | protein autophosphorylation | 134 | 9 | 0.00977 |
| GO:0008152 | metabolic process | 11080 | 259 | 0.0101 |
| GO:0009607 | response to biotic stimulus | 779 | 22 | 0.01126 |
| GO:0052543 | callose deposition in cell wall | 36 | 4 | 0.01498 |
| GO:0000911 | cytokinesis by cell plate formation | 56 | 5 | 0.01652 |
| GO:0005983 | starch catabolic process | 23 | 3 | 0.02245 |
| GO:0032501 | multicellular organismal process | 2433 | 63 | 0.0269 |
| GO:0050794 | regulation of cellular process | 4151 | 91 | 0.02715 |
| GO:1901002 | positive regulation of response to salt stress | 10 | 2 | 0.02772 |
| GO:0000731 | DNA synthesis involved in DNA repair | 10 | 2 | 0.02772 |
| GO:0043043 | peptide biosynthetic process | 672 | 14 | 0.02848 |
| GO:0000226 | microtubule cytoskeleton organization | 89 | 6 | 0.03163 |
| GO:0030004 | cellular monovalent inorganic cation homeostasis | 37 | 3 | 0.03324 |
| GO:0051570 | regulation of histone H3-K9 methylation | 11 | 2 | 0.03329 |
| GO:0010584 | pollen exine formation | 27 | 3 | 0.03431 |
| GO:0051260 | protein homooligomerization | 28 | 3 | 0.03769 |
| GO:0052325 | cell wall pectin biosynthetic process | 12 | 2 | 0.03926 |
| GO:0009863 | salicylic acid mediated signaling pathway | 49 | 4 | 0.04122 |
| GO:0016567 | protein ubiquitination | 416 | 16 | 0.04392 |
| GO:0032973 | amino acid export | 13 | 2 | 0.04559 |
| GO:0016020 | membrane | 8257 | 204 | 5.80E-05 |
| GO:0044444 | cytoplasmic part | 7933 | 160 | 0.0013 |
| GO:0016459 | myosin complex | 23 | 4 | 0.0022 |
| GO:0044446 | intracellular organelle part | 4272 | 83 | 0.0065 |
| GO:0019013 | viral nucleocapsid | 24 | 3 | 0.0202 |
| GO:0043229 | intracellular organelle | 12614 | 285 | 0.021 |
| GO:0031595 | nuclear proteasome complex | 11 | 2 | 0.0285 |
| GO:0031597 | cytosolic proteasome complex | 11 | 2 | 0.0285 |
| GO:0043227 | membrane-bounded organelle | 12177 | 273 | 0.0289 |
| GO:0043231 | intracellular membrane-bounded organelle | 12116 | 271 | 0.043 |
| GO:0009504 | cell plate | 33 | 3 | 0.0464 |
| GO:0005737 | cytoplasm | 9554 | 216 | 0.0472 |
| GO:0019867 | outer membrane | 86 | 5 | 0.0482 |
| GO:0005911 | cell-cell junction | 775 | 16 | 0.0489 |
| GO:0005488 | binding | 10381 | 244 | 1.60E-06 |
| GO:0009931 | calcium-dependent protein serine/threonine kinase activity | 34 | 8 | 1.60E-06 |
| GO:0004683 | calmodulin-dependent protein kinase activity | 37 | 8 | 3.20E-06 |
| GO:0016740 | transferase activity | 3639 | 102 | 0.00027 |
| GO:0003774 | motor activity | 88 | 7 | 0.00114 |
| GO:0000166 | nucleotide binding | 3346 | 78 | 0.00389 |
| GO:0043167 | ion binding | 5993 | 125 | 0.00411 |
| GO:0005516 | calmodulin binding | 200 | 12 | 0.005 |
| GO:0008146 | sulfotransferase activity | 31 | 4 | 0.00739 |
| GO:0005216 | ion channel activity | 114 | 6 | 0.01774 |
| GO:0051119 | sugar transmembrane transporter activity | 91 | 5 | 0.0195 |
| GO:0016881 | acid-amino acid ligase activity | 25 | 3 | 0.02446 |
| GO:0016653 | oxidoreductase activity, acting on NAD(P)H, heme protein as acceptor | 10 | 2 | 0.02511 |
| GO:0004553 | hydrolase activity, hydrolyzing O-glycosyl compounds | 415 | 16 | 0.02983 |
| GO:0016799 | hydrolase activity, hydrolyzing N-glycosyl compounds | 34 | 3 | 0.03012 |
| GO:0036402 | proteasome-activating ATPase activity | 11 | 2 | 0.03018 |
| GO:0016892 | endoribonuclease activity, producing 3'-phosphomonoesters | 12 | 2 | 0.03562 |
| GO:0016730 | oxidoreductase activity, acting on iron-sulfur proteins as donors | 12 | 2 | 0.03562 |
| GO:0003887 | DNA-directed DNA polymerase activity | 30 | 3 | 0.03938 |
| GO:0016207 | 4-coumarate-CoA ligase activity | 13 | 2 | 0.04141 |
| GO:0035064 | methylated histone binding | 13 | 2 | 0.04141 |
| GO:0042285 | xylosyltransferase activity | 13 | 2 | 0.04141 |
| GO:0016760 | cellulose synthase (UDP-forming) activity | 31 | 3 | 0.0428 |
|  |  |  |  |  |

**Supplementary Table 12. GO enrichment of significantly expanded genes in *L. maritima* compared to other related species.**

| **GO terms** | **Function** | **Annotated** | **Significant** | **P value** |
| --- | --- | --- | --- | --- |
| GO:0002238 | response to molecule of fungal origin | 12 | 8 | 5.70E-16 |
| GO:0080156 | mitochondrial mRNA modification | 28 | 9 | 4.00E-14 |
| GO:0015074 | DNA integration | 31 | 9 | 1.20E-13 |
| GO:0009625 | response to insect | 25 | 8 | 1.20E-12 |
| GO:0002237 | response to molecule of bacterial origin | 35 | 8 | 2.40E-11 |
| GO:0009624 | response to nematode | 59 | 8 | 2.00E-09 |
| GO:0042542 | response to hydrogen peroxide | 64 | 8 | 4.00E-09 |
| GO:0012501 | programmed cell death | 140 | 9 | 6.90E-09 |
| GO:0046950 | cellular ketone body metabolic process | 13 | 5 | 8.40E-09 |
| GO:0009960 | endosperm development | 26 | 5 | 4.00E-07 |
| GO:0018874 | benzoate metabolic process | 37 | 5 | 2.50E-06 |
| GO:0006635 | fatty acid beta-oxidation | 41 | 5 | 4.30E-06 |
| GO:0007169 | transmembrane receptor protein tyrosine kinase signaling pathway | 122 | 7 | 8.20E-06 |
| GO:0009611 | response to wounding | 179 | 8 | 1.10E-05 |
| GO:0009751 | response to salicylic acid | 184 | 8 | 1.40E-05 |
| GO:0006550 | isoleucine catabolic process | 53 | 5 | 1.60E-05 |
| GO:0010951 | negative regulation of endopeptidase activity | 26 | 4 | 1.60E-05 |
| GO:0006574 | valine catabolic process | 54 | 5 | 1.70E-05 |
| GO:0006552 | leucine catabolic process | 55 | 5 | 1.90E-05 |
| GO:0006397 | mRNA processing | 252 | 9 | 1.90E-05 |
| GO:0006554 | lysine catabolic process | 58 | 5 | 2.40E-05 |
| GO:0019915 | lipid storage | 16 | 3 | 0.00011 |
| GO:0009809 | lignin biosynthetic process | 97 | 5 | 0.00028 |
| GO:0042742 | defense response to bacterium | 317 | 8 | 0.00061 |
| GO:0006568 | tryptophan metabolic process | 142 | 5 | 0.0016 |
| GO:0032259 | methylation | 394 | 8 | 0.00243 |
| GO:0006090 | pyruvate metabolic process | 186 | 5 | 0.00512 |
| GO:0006633 | fatty acid biosynthetic process | 206 | 5 | 0.00782 |
| GO:0009069 | serine family amino acid metabolic process | 1031 | 13 | 0.00803 |
| GO:0009651 | response to salt stress | 488 | 8 | 0.00869 |
| GO:0019953 | sexual reproduction | 143 | 4 | 0.01058 |
| GO:0042967 | acyl-carrier-protein biosynthetic process | 254 | 5 | 0.01802 |
| GO:0048544 | recognition of pollen | 35 | 2 | 0.01847 |
| GO:0006468 | protein phosphorylation | 1165 | 13 | 0.0206 |
| GO:0006833 | water transport | 40 | 2 | 0.02375 |
| GO:0006351 | transcription, DNA-templated | 2192 | 12 | 0.04716 |
| GO:0004866 | endopeptidase inhibitor activity | 33 | 8 | 9.80E-11 |
| GO:0008171 | O-methyltransferase activity | 41 | 8 | 6.40E-10 |
| GO:0003988 | acetyl-CoA C-acyltransferase activity | 10 | 5 | 5.50E-09 |
| GO:0016747 | transferase activity, transferring acyl groups other than amino-acyl groups | 378 | 15 | 9.10E-06 |
| GO:0004519 | endonuclease activity | 222 | 9 | 4.70E-05 |
| GO:0003676 | nucleic acid binding | 3332 | 29 | 6.70E-05 |
| GO:0004497 | monooxygenase activity | 291 | 10 | 7.10E-05 |
| GO:0005506 | iron ion binding | 332 | 10 | 0.00021 |
| GO:0020037 | heme binding | 336 | 10 | 0.00023 |
| GO:0016866 | intramolecular transferase activity | 59 | 4 | 0.00102 |
| GO:0016407 | acetyltransferase activity | 133 | 5 | 0.00336 |
| GO:0009975 | cyclase activity | 14 | 2 | 0.00485 |
| GO:0046983 | protein dimerization activity | 497 | 9 | 0.01298 |
| GO:0004674 | protein serine/threonine kinase activity | 899 | 13 | 0.01804 |
| GO:0016788 | hydrolase activity, acting on ester bonds | 1085 | 19 | 0.04868 |
| GO:0012511 | monolayer-surrounded lipid storage body | 23 | 4 | 8.00E-06 |
| GO:0005739 | mitochondrion | 2035 | 23 | 0.00091 |
|  |  |  |  |  |

**Supplementary Table 13. Function of genes with homolog in Swiss-prot database in significantly expanded gene families of *L. maritima.***

| **Gene ID** | **Swiss-Prot Annotation** | **Gene Function** |
| --- | --- | --- |
| Lma22722.t1 | Y3475_ARATH | NA |
| Lma00685.t1 | POLR1_ARATH | NA |
| Lma09250.t1 | M310_ARATH | NA |
| Lma14759.t1 | ALPL_ARATH | Transposase-derived protein that may have nuclease activity. |
| Lma22625.t1 | KTI1_ARATH | Exhibits Kunitz trypsin protease inhibitor activity. Involved in modulating programmed cell death (PCD) in plant- pathogen interactions. |
| Lma20473.t1 | Y3720_ARATH | NA |
| Lma00190.t1 | THAH_ARATH | Hydroxylates thalianol into thalian-diol. |
| Lma20357.t1 | RTM3_ARATH | Required for the restriction of long-distance movement of the pathogenic tobacco etch virus (TEV) without causing a hypersensitive response or inducing systemic acquired resistance. |
| Lma03977.t1 | GH312_ARATH | Catalyzes the conjugation of specific amino acids (e.g. Glu and possibly His, Lys, and Met) to their preferred acyl substrates (e.g. 4-substituted benzoates), in a magnesium ion- and ATP-dependent manner. Can use 4-substituted benzoates such as 4- aminobenzoate (pABA), 4-fluorobenzoate and 4-hydroxybenzoate (4- HBA), and, to a lesser extent, benzoate, vanillate and trans- cinnamate, but not 2-substituted benzoates and salicylic acid (SA), as conjugating acyl substrates. Involved in both basal and induced resistance in a SA-dependent manner. Confers resistance to virulent and avirulent pathogens (at least bacteria and oomycetes), and promotes SA glucosides accumulation. Required for the establishment of hyper-sensitive response (HR) upon incompatible interaction and subsequent systemic acquired resistance (SAR). |
| Lma12284.t1 | RNHX1_ARATH | NA |
| Lma04403.t1 | KTI1_ARATH | Exhibits Kunitz trypsin protease inhibitor activity. Involved in modulating programmed cell death (PCD) in plant- pathogen interactions. |
| Lma15769.t1 | POLX_TOBAC | NA |
| Lma00410.t1 | GH312_ARATH | Catalyzes the conjugation of specific amino acids (e.g. Glu and possibly His, Lys, and Met) to their preferred acyl substrates (e.g. 4-substituted benzoates), in a magnesium ion- and ATP-dependent manner. Can use 4-substituted benzoates such as 4- aminobenzoate (pABA), 4-fluorobenzoate and 4-hydroxybenzoate (4- HBA), and, to a lesser extent, benzoate, vanillate and trans- cinnamate, but not 2-substituted benzoates and salicylic acid (SA), as conjugating acyl substrates. Involved in both basal and induced resistance in a SA-dependent manner. Confers resistance to virulent and avirulent pathogens (at least bacteria and oomycetes), and promotes SA glucosides accumulation. Required for the establishment of hyper-sensitive response (HR) upon incompatible interaction and subsequent systemic acquired resistance (SAR). |
| Lma06542.t1 | GH312_ARATH | Catalyzes the conjugation of specific amino acids (e.g. Glu and possibly His, Lys, and Met) to their preferred acyl substrates (e.g. 4-substituted benzoates), in a magnesium ion- and ATP-dependent manner. Can use 4-substituted benzoates such as 4- aminobenzoate (pABA), 4-fluorobenzoate and 4-hydroxybenzoate (4- HBA), and, to a lesser extent, benzoate, vanillate and trans- cinnamate, but not 2-substituted benzoates and salicylic acid (SA), as conjugating acyl substrates. Involved in both basal and induced resistance in a SA-dependent manner. Confers resistance to virulent and avirulent pathogens (at least bacteria and oomycetes), and promotes SA glucosides accumulation. Required for the establishment of hyper-sensitive response (HR) upon incompatible interaction and subsequent systemic acquired resistance (SAR). |
| Lma11339.t1 | Y3475_ARATH | NA |
| Lma20358.t1 | MCC30_ARATH | NA |
| Lma03536.t1 | R13L4_ARATH | CC-NB-LRR receptor-like protein required for recognition of the Pseudomonas syringae type III effector HopZ1a. Confers allele-specific recognition and virulence attenuation of HopZ1a. Immunity mediated by ZAR1 is independent of several genes required by other resistance protein signaling pathways such as NDR1 and RAR1. |
| Lma16566.t1 | POLX_TOBAC | NA |
| Lma03537.t1 | ALPL_ARATH | Transposase-derived protein that may have nuclease activity. |
| Lma05693.t1 | EFR_ARATH | Constitutes the pattern-recognition receptor (PPR) that determines the specific perception of elongation factor Tu (EF- Tu), a potent elicitor of the defense response to pathogen- associated molecular patterns (PAMPs). Reduces transformation by Rhizobium radiobacter probably by inducing plant defense during the interaction. Binding to the effector AvrPto1 from P.syringae blocks the downstream plant immune response while interaction with hopD2 decreases the phosphorylation level of EFR upon elf18 treatment. Specific endoplasmic reticulum quality control components (ERD2B, CRT3, UGGT and STT3A) are required for the biogenesis of EFR. |
| Lma26020.t1 | Y3475_ARATH | NA |
| Lma06977.t1 | PP339_ARATH | NA |
| Lma17746.t1 | PPR94_ARATH | NA |
| Lma21662.t1 | R13L4_ARATH | CC-NB-LRR receptor-like protein required for recognition of the Pseudomonas syringae type III effector HopZ1a. Confers allele-specific recognition and virulence attenuation of HopZ1a. Immunity mediated by ZAR1 is independent of several genes required by other resistance protein signaling pathways such as NDR1 and RAR1. |
| Lma17617.t1 | WRK10_ARATH | Transcription factor. Interacts specifically with the W box (5'-(T)TGAC[CT]-3'), a frequently occurring elicitor- responsive cis-acting element (By similarity). Modulates seed size by negatively regulating the cellularization of syncytial endosperm. |
| Lma15100.t1 | THAH_ARATH | Hydroxylates thalianol into thalian-diol. |
| Lma17002.t1 | POLX_TOBAC | NA |
| Lma14359.t1 | Y3720_ARATH | NA |
| Lma02730.t1 | POLX_TOBAC | NA |
| Lma12114.t1 | POLX_TOBAC | NA |
| Lma03543.t1 | R13L4_ARATH | CC-NB-LRR receptor-like protein required for recognition of the Pseudomonas syringae type III effector HopZ1a. Confers allele-specific recognition and virulence attenuation of HopZ1a. Immunity mediated by ZAR1 is independent of several genes required by other resistance protein signaling pathways such as NDR1 and RAR1. |
| Lma25942.t1 | POLX_TOBAC | NA |
| Lma04814.t1 | AGL80_ARATH | Probable transcription factor. Controls central cell differentiation during female gametophyte development. Required for the expression of DEMETER and DD46, but not for the expression of FIS2. Probable transcription factor that may function in the maintenance of the proper function of the central cell in pollen tube attraction (Probable). |
| Lma15160.t1 | M310_ARATH | NA |
| Lma05305.t1 | MCC17_ARATH | NA |
| Lma12414.t1 | FB181_ARATH | NA |
| Lma00885.t1 | POLR1_ARATH | NA |
| Lma10119.t1 | POLX_TOBAC | NA |
| Lma25410.t1 | PP412_ARATH | NA |
| Lma00188.t1 | BAHD1_ARATH | Probably involved in the modification of desaturated thalian-diol. |
| Lma11317.t1 | Y3471_ARATH | NA |
| Lma00199.t1 | M310_ARATH | NA |
| Lma26087.t1 | POLX_TOBAC | NA |
| Lma07014.t1 | POLX_TOBAC | NA |
| Lma20344.t1 | MCC13_ARATH | NA |
| Lma22780.t1 | RNHX1_ARATH | NA |
| Lma14894.t1 | WRK10_ARATH | Transcription factor. Interacts specifically with the W box (5'-(T)TGAC[CT]-3'), a frequently occurring elicitor- responsive cis-acting element (By similarity). Modulates seed size by negatively regulating the cellularization of syncytial endosperm. |
| Lma00030.t1 | WRK10_ARATH | Transcription factor. Interacts specifically with the W box (5'-(T)TGAC[CT]-3'), a frequently occurring elicitor- responsive cis-acting element (By similarity). Modulates seed size by negatively regulating the cellularization of syncytial endosperm. |
| Lma20356.t1 | MCC25_ARATH | NA |
| Lma11207.t1 | BAHD2_ARATH | NA |
| Lma06453.t1 | IRK_ARATH | NA |
| Lma12415.t1 | FB181_ARATH | NA |
| Lma09149.t1 | R13L4_ARATH | CC-NB-LRR receptor-like protein required for recognition of the Pseudomonas syringae type III effector HopZ1a. Confers allele-specific recognition and virulence attenuation of HopZ1a. Immunity mediated by ZAR1 is independent of several genes required by other resistance protein signaling pathways such as NDR1 and RAR1. |
| Lma25677.t1 | KTI1_ARATH | Exhibits Kunitz trypsin protease inhibitor activity. Involved in modulating programmed cell death (PCD) in plant- pathogen interactions. |
| Lma14358.t1 | Y3720_ARATH | NA |
| Lma05454.t1 | POLX_TOBAC | NA |
| Lma26482.t1 | PEN7_ARATH | NA |
| Lma17686.t1 | PPR91_ARATH | NA |
| Lma03988.t1 | ALPL_ARATH | Transposase-derived protein that may have nuclease activity. |
| Lma04225.t1 | M310_ARATH | NA |
| Lma17615.t1 | WRK10_ARATH | Transcription factor. Interacts specifically with the W box (5'-(T)TGAC[CT]-3'), a frequently occurring elicitor- responsive cis-acting element (By similarity). Modulates seed size by negatively regulating the cellularization of syncytial endosperm. |
| Lma04439.t1 | BAHD2_ARATH | NA |
| Lma20354.t1 | MCC28_ARATH | NA |
| Lma22621.t1 | KTI1_ARATH | Exhibits Kunitz trypsin protease inhibitor activity. Involved in modulating programmed cell death (PCD) in plant- pathogen interactions. |
| Lma22531.t1 | RTM3_ARATH | Required for the restriction of long-distance movement of the pathogenic tobacco etch virus (TEV) without causing a hypersensitive response or inducing systemic acquired resistance. |
| Lma11080.t1 | PPP7L_ARATH | Maybe required to maintain cell division activity in meristematic cells. |
| Lma22622.t1 | KTI1_ARATH | Exhibits Kunitz trypsin protease inhibitor activity. Involved in modulating programmed cell death (PCD) in plant- pathogen interactions. |
| Lma19036.t1 | OLEO3_ARATH | May have a structural role to stabilize the lipid body during desiccation of the seed by preventing coalescence of the oil. Probably interacts with both lipid and phospholipid moieties of lipid bodies. May also provide recognition signals for specific lipase anchorage in lipolysis during seedling growth (By similarity). |
| Lma24837.t1 | M310_ARATH | NA |
| Lma11571.t1 | GRP17_ARATH | Lipid-binding oleosin pollen coat protein required to mediate pollen recognition by stigma cells and subsequent pollen hydration. |
| Lma20343.t1 | MCC13_ARATH | NA |
| Lma11341.t1 | Y3475_ARATH | NA |
| Lma08104.t1 | Y3720_ARATH | NA |
| Lma06298.t1 | AGL80_ARATH | Probable transcription factor. Controls central cell differentiation during female gametophyte development. Required for the expression of DEMETER and DD46, but not for the expression of FIS2. Probable transcription factor that may function in the maintenance of the proper function of the central cell in pollen tube attraction (Probable). |
| Lma18134.t1 | RNHX1_ARATH | NA |
| Lma05515.t1 | POLX_TOBAC | NA |
| Lma21930.t1 | IGMT1_ARATH | Involved in indole glucosinolate biosynthesis. Catalyzes methoxylation reactions of the glucosinolate indole ring. Converts the hydroxy intermediates 4-hydroxy-indol-3-yl-methylglucosinolate (4OH-I3M) and 1-hydroxy-indol-3-yl-methylglucosinolate (1OH-I3M) to 4-methoxy-indol-3-yl-methylglucosinolate (4MO-I3M) and 1- methoxy-indol-3-yl-methylglucosinolate (1MO-I3M), respectively. |
| Lma00195.t1 | BIA1_ARATH | Monitors brassinosteroids (BR) responses and homeostasis, particularly in the root and hypocotyl in darkness. Promotes flavonoid biosynthesis. |
| Lma23153.t1 | BIA1_ARATH | Monitors brassinosteroids (BR) responses and homeostasis, particularly in the root and hypocotyl in darkness. Promotes flavonoid biosynthesis. |
| Lma25686.t1 | PP339_ARATH | NA |
| Lma15102.t1 | THAH_ARATH | Hydroxylates thalianol into thalian-diol. |
| Lma22721.t1 | Y3475_ARATH | NA |
| Lma21664.t1 | R13L4_ARATH | CC-NB-LRR receptor-like protein required for recognition of the Pseudomonas syringae type III effector HopZ1a. Confers allele-specific recognition and virulence attenuation of HopZ1a. Immunity mediated by ZAR1 is independent of several genes required by other resistance protein signaling pathways such as NDR1 and RAR1. |
| Lma13169.t1 | POLX_TOBAC | NA |
| Lma22874.t1 | Y3720_ARATH | NA |
| Lma00255.t1 | PPP7L_ARATH | Maybe required to maintain cell division activity in meristematic cells. |
| Lma19734.t1 | PPP7L_ARATH | Maybe required to maintain cell division activity in meristematic cells. |
| Lma18994.t1 | THAH_ARATH | Hydroxylates thalianol into thalian-diol. |
| Lma00254.t1 | PPP7L_ARATH | Maybe required to maintain cell division activity in meristematic cells. |
| Lma10879.t1 | FB181_ARATH | NA |
| Lma24212.t1 | ALPL_ARATH | Transposase-derived protein that may have nuclease activity. |
| Lma14914.t1 | POLR1_ARATH | NA |
| Lma20345.t1 | MCC14_ARATH | NA |
| Lma10812.t1 | POLR2_ARATH | NA |
| Lma08349.t1 | POLR1_ARATH | NA |
| Lma14646.t1 | M1250_ARATH | NA |
| Lma10942.t1 | PHE2_ARATH | Probable transcription factor involved in the development of gametophytes and seeds. |
| Lma21611.t1 | Y3475_ARATH | NA |
| Lma24712.t1 | PEN7_ARATH | NA |
| Lma11569.t1 | OLNB1_BRANA | Many of the major pollen coat proteins are derived from endoproteolytic cleavage of oleosin-like proteins. |
| Lma12410.t1 | AGL80_ARATH | Probable transcription factor. Controls central cell differentiation during female gametophyte development. Required for the expression of DEMETER and DD46, but not for the expression of FIS2. Probable transcription factor that may function in the maintenance of the proper function of the central cell in pollen tube attraction (Probable). |
| Lma00189.t1 | PEN7_ARATH | NA |
| Lma05304.t1 | MCC21_ARATH | NA |
| Lma24266.t1 | PPP7L_ARATH | Maybe required to maintain cell division activity in meristematic cells. |
| Lma18772.t1 | Y3475_ARATH | NA |
| Lma12443.t1 | M310_ARATH | NA |
| Lma17317.t1 | MCC31_ARATH | NA |
| Lma19038.t1 | OLEO3_ARATH | May have a structural role to stabilize the lipid body during desiccation of the seed by preventing coalescence of the oil. Probably interacts with both lipid and phospholipid moieties of lipid bodies. May also provide recognition signals for specific lipase anchorage in lipolysis during seedling growth (By similarity). |
| Lma05306.t1 | MCC19_ARATH | NA |
| Lma04438.t1 | BAHD2_ARATH | NA |
| Lma18661.t1 | GH312_ARATH | Catalyzes the conjugation of specific amino acids (e.g. Glu and possibly His, Lys, and Met) to their preferred acyl substrates (e.g. 4-substituted benzoates), in a magnesium ion- and ATP-dependent manner. Can use 4-substituted benzoates such as 4- aminobenzoate (pABA), 4-fluorobenzoate and 4-hydroxybenzoate (4- HBA), and, to a lesser extent, benzoate, vanillate and trans- cinnamate, but not 2-substituted benzoates and salicylic acid (SA), as conjugating acyl substrates. Involved in both basal and induced resistance in a SA-dependent manner. Confers resistance to virulent and avirulent pathogens (at least bacteria and oomycetes), and promotes SA glucosides accumulation. Required for the establishment of hyper-sensitive response (HR) upon incompatible interaction and subsequent systemic acquired resistance (SAR). |
| Lma20352.t1 | RTM3_ARATH | Required for the restriction of long-distance movement of the pathogenic tobacco etch virus (TEV) without causing a hypersensitive response or inducing systemic acquired resistance. |
| Lma23124.t1 | BAHD2_ARATH | NA |
| Lma13469.t1 | POLR1_ARATH | NA |
| Lma24891.t1 | FLOR1_ARATH | Promotes flowering transition in long days (LD). |
| Lma15172.t1 | POLX_TOBAC | NA |
| Lma11012.t1 | Y3720_ARATH | NA |
| Lma22105.t1 | BAHD2_ARATH | NA |
| Lma07591.t1 | POLX_TOBAC | NA |
| Lma19502.t1 | POLX_TOBAC | NA |
| Lma19487.t1 | POLR2_ARATH | NA |
| Lma23147.t1 | POLX_TOBAC | NA |
| Lma14720.t1 | EFR_ARATH | Constitutes the pattern-recognition receptor (PPR) that determines the specific perception of elongation factor Tu (EF- Tu), a potent elicitor of the defense response to pathogen- associated molecular patterns (PAMPs). Reduces transformation by Rhizobium radiobacter probably by inducing plant defense during the interaction. Binding to the effector AvrPto1 from P.syringae blocks the downstream plant immune response while interaction with hopD2 decreases the phosphorylation level of EFR upon elf18 treatment. Specific endoplasmic reticulum quality control components (ERD2B, CRT3, UGGT and STT3A) are required for the biogenesis of EFR. |
| Lma21563.t1 | WRK10_ARATH | Transcription factor. Interacts specifically with the W box (5'-(T)TGAC[CT]-3'), a frequently occurring elicitor- responsive cis-acting element (By similarity). Modulates seed size by negatively regulating the cellularization of syncytial endosperm. |
| Lma12335.t1 | PPR99_ARATH | NA |
| Lma26034.t1 | PPR99_ARATH | NA |
| Lma06691.t1 | IGMT3_ARATH | Involved in indole glucosinolate biosynthesis. Catalyzes methoxylation reactions of the glucosinolate indole ring. Converts the hydroxy intermediates 4-hydroxy-indol-3-yl-methylglucosinolate (4OH-I3M) and 1-hydroxy-indol-3-yl-methylglucosinolate (1OH-I3M) to 4-methoxy-indol-3-yl-methylglucosinolate (4MO-I3M) and 1- methoxy-indol-3-yl-methylglucosinolate(1MO-I3M), respectively. |
| Lma19037.t1 | OLEO3_ARATH | May have a structural role to stabilize the lipid body during desiccation of the seed by preventing coalescence of the oil. Probably interacts with both lipid and phospholipid moieties of lipid bodies. May also provide recognition signals for specific lipase anchorage in lipolysis during seedling growth (By similarity). |
| Lma18659.t1 | GH312_ARATH | Catalyzes the conjugation of specific amino acids (e.g. Glu and possibly His, Lys, and Met) to their preferred acyl substrates (e.g. 4-substituted benzoates), in a magnesium ion- and ATP-dependent manner. Can use 4-substituted benzoates such as 4- aminobenzoate (pABA), 4-fluorobenzoate and 4-hydroxybenzoate (4- HBA), and, to a lesser extent, benzoate, vanillate and trans- cinnamate, but not 2-substituted benzoates and salicylic acid (SA), as conjugating acyl substrates. Involved in both basal and induced resistance in a SA-dependent manner. Confers resistance to virulent and avirulent pathogens (at least bacteria and oomycetes), and promotes SA glucosides accumulation. Required for the establishment of hyper-sensitive response (HR) upon incompatible interaction and subsequent systemic acquired resistance (SAR). |
| Lma00678.t1 | M310_ARATH | NA |
| Lma06690.t1 | IGMT4_ARATH | Involved in indole glucosinolate biosynthesis. Catalyzes methoxylation reactions of the glucosinolate indole ring. Converts the hydroxy intermediates 4-hydroxy-indol-3-yl-methylglucosinolate (4OH-I3M) and 1-hydroxy-indol-3-yl-methylglucosinolate (1OH-I3M) to 4-methoxy-indol-3-yl-methylglucosinolate (4MO-I3M) and 1- methoxy-indol-3-yl-methylglucosinolate(1MO-I3M), respectively. |
| Lma11570.t1 | GRP17_ARATH | Lipid-binding oleosin pollen coat protein required to mediate pollen recognition by stigma cells and subsequent pollen hydration. |
| Lma09996.t1 | POLX_TOBAC | NA |
| Lma12975.t1 | HIP14_ARATH | Probable heavy-metal-binding protein. |
| Lma00595.t1 | AGL80_ARATH | Probable transcription factor. Controls central cell differentiation during female gametophyte development. Required for the expression of DEMETER and DD46, but not for the expression of FIS2. Probable transcription factor that may function in the maintenance of the proper function of the central cell in pollen tube attraction (Probable). |
| Lma24890.t1 | FLOR1_ARATH | Promotes flowering transition in long days (LD). |
| Lma25790.t1 | GH312_ARATH | Catalyzes the conjugation of specific amino acids (e.g. Glu and possibly His, Lys, and Met) to their preferred acyl substrates (e.g. 4-substituted benzoates), in a magnesium ion- and ATP-dependent manner. Can use 4-substituted benzoates such as 4- aminobenzoate (pABA), 4-fluorobenzoate and 4-hydroxybenzoate (4- HBA), and, to a lesser extent, benzoate, vanillate and trans- cinnamate, but not 2-substituted benzoates and salicylic acid (SA), as conjugating acyl substrates. Involved in both basal and induced resistance in a SA-dependent manner. Confers resistance to virulent and avirulent pathogens (at least bacteria and oomycetes), and promotes SA glucosides accumulation. Required for the establishment of hyper-sensitive response (HR) upon incompatible interaction and subsequent systemic acquired resistance (SAR). |
| Lma03771.t1 | M310_ARATH | NA |
| Lma00285.t1 | M310_ARATH | NA |
| Lma20350.t1 | MCC19_ARATH | NA |
| Lma23158.t2 | THAH_ARATH | Hydroxylates thalianol into thalian-diol. |
| Lma09132.t1 | BAHD2_ARATH | NA |
| Lma19505.t1 | Y3475_ARATH | NA |
| Lma03767.t1 | AGL80_ARATH | Probable transcription factor. Controls central cell differentiation during female gametophyte development. Required for the expression of DEMETER and DD46, but not for the expression of FIS2. Probable transcription factor that may function in the maintenance of the proper function of the central cell in pollen tube attraction (Probable). |
| Lma12338.t1 | PPR96_ARATH | NA |
| Lma03335.t1 | M310_ARATH | NA |
| Lma17720.t1 | PPR93_ARATH | NA |
| Lma09152.t1 | R13L4_ARATH | CC-NB-LRR receptor-like protein required for recognition of the Pseudomonas syringae type III effector HopZ1a. Confers allele-specific recognition and virulence attenuation of HopZ1a. Immunity mediated by ZAR1 is independent of several genes required by other resistance protein signaling pathways such as NDR1 and RAR1. |
| Lma10334.t1 | M310_ARATH | NA |
| Lma06352.t1 | PPR96_ARATH | NA |
| Lma17684.t1 | PPR99_ARATH | NA |
| Lma11222.t1 | BAHD2_ARATH | NA |
| Lma11316.t1 | Y3471_ARATH | NA |
| Lma20347.t1 | MCC25_ARATH | NA |
| Lma02785.t1 | THAH_ARATH | Hydroxylates thalianol into thalian-diol. |
| Lma01338.t1 | M310_ARATH | NA |
| Lma14721.t1 | Y3475_ARATH | NA |
| Lma13226.t1 | Y3475_ARATH | NA |
| Lma00411.t1 | GH312_ARATH | Catalyzes the conjugation of specific amino acids (e.g. Glu and possibly His, Lys, and Met) to their preferred acyl substrates (e.g. 4-substituted benzoates), in a magnesium ion- and ATP-dependent manner. Can use 4-substituted benzoates such as 4- aminobenzoate (pABA), 4-fluorobenzoate and 4-hydroxybenzoate (4- HBA), and, to a lesser extent, benzoate, vanillate and trans- cinnamate, but not 2-substituted benzoates and salicylic acid (SA), as conjugating acyl substrates. Involved in both basal and induced resistance in a SA-dependent manner. Confers resistance to virulent and avirulent pathogens (at least bacteria and oomycetes), and promotes SA glucosides accumulation. Required for the establishment of hyper-sensitive response (HR) upon incompatible interaction and subsequent systemic acquired resistance (SAR). |
| Lma03809.t1 | ALPL_ARATH | Transposase-derived protein that may have nuclease activity. |
| Lma20351.t1 | MCC23_ARATH | NA |
| Lma17735.t1 | PPR91_ARATH | NA |
| Lma15096.t1 | BAHD1_ARATH | Probably involved in the modification of desaturated thalian-diol. |
| Lma20046.t1 | Y3475_ARATH | NA |
| Lma17616.t1 | WRK10_ARATH | Transcription factor. Interacts specifically with the W box (5'-(T)TGAC[CT]-3'), a frequently occurring elicitor- responsive cis-acting element (By similarity). Modulates seed size by negatively regulating the cellularization of syncytial endosperm. |
| Lma15103.t2 | THAH_ARATH | Hydroxylates thalianol into thalian-diol. |
| Lma08005.t1 | MCC26_ARATH | NA |
| Lma05840.t1 | M310_ARATH | NA |
| Lma05958.t1 | OLEO3_ARATH | May have a structural role to stabilize the lipid body during desiccation of the seed by preventing coalescence of the oil. Probably interacts with both lipid and phospholipid moieties of lipid bodies. May also provide recognition signals for specific lipase anchorage in lipolysis during seedling growth (By similarity). |
| Lma04437.t1 | BAHD2_ARATH | NA |
| Lma04406.t1 | KTI1_ARATH | Exhibits Kunitz trypsin protease inhibitor activity. Involved in modulating programmed cell death (PCD) in plant- pathogen interactions. |
| Lma05121.t1 | M310_ARATH | NA |
| Lma12337.t1 | PPR96_ARATH | NA |
| Lma17688.t1 | PPR91_ARATH | NA |
| Lma24238.t1 | POLX_TOBAC | NA |
| Lma22306.t1 | M310_ARATH | NA |
| Lma14633.t1 | BAHD2_ARATH | NA |
| Lma19417.t1 | POLR1_ARATH | NA |
| Lma22918.t1 | COMT1_PRUDU | Catalyzes the conversion of caffeic acid to ferulic acid and of 5-hydroxyferulic acid to sinapic acid. The resulting products may subsequently be converted to the corresponding alcohols that are incorporated into lignins. |
| Lma25863.t1 | POLR1_ARATH | NA |
| Lma17747.t1 | PPR90_ARATH | NA |
| Lma22624.t1 | KTI1_ARATH | Exhibits Kunitz trypsin protease inhibitor activity. Involved in modulating programmed cell death (PCD) in plant- pathogen interactions. |
| Lma05622.t1 | POLX_TOBAC | NA |
| Lma12554.t1 | M310_ARATH | NA |
| Lma06253.t1 | POLX_TOBAC | NA |
| Lma10871.t1 | RTM3_ARATH | Required for the restriction of long-distance movement of the pathogenic tobacco etch virus (TEV) without causing a hypersensitive response or inducing systemic acquired resistance. |
| Lma01327.t1 | POLX_TOBAC | NA |
| Lma09145.t1 | R13L4_ARATH | CC-NB-LRR receptor-like protein required for recognition of the Pseudomonas syringae type III effector HopZ1a. Confers allele-specific recognition and virulence attenuation of HopZ1a. Immunity mediated by ZAR1 is independent of several genes required by other resistance protein signaling pathways such as NDR1 and RAR1. |
| Lma00460.t1 | AGL80_ARATH | Probable transcription factor. Controls central cell differentiation during female gametophyte development. Required for the expression of DEMETER and DD46, but not for the expression of FIS2. Probable transcription factor that may function in the maintenance of the proper function of the central cell in pollen tube attraction (Probable). |
| Lma23152.t1 | BIA1_ARATH | Monitors brassinosteroids (BR) responses and homeostasis, particularly in the root and hypocotyl in darkness. Promotes flavonoid biosynthesis. |
| Lma10744.t1 | GSTT3_ARATH | May be involved in the conjugation of reduced glutathione to a wide number of exogenous and endogenous hydrophobic electrophiles and have a detoxification role against certain herbicides. |
| Lma08105.t1 | Y3720_ARATH | NA |
| Lma00596.t1 | AGL80_ARATH | Probable transcription factor. Controls central cell differentiation during female gametophyte development. Required for the expression of DEMETER and DD46, but not for the expression of FIS2. Probable transcription factor that may function in the maintenance of the proper function of the central cell in pollen tube attraction (Probable). |
| Lma00160.t1 | GH312_ARATH | Catalyzes the conjugation of specific amino acids (e.g. Glu and possibly His, Lys, and Met) to their preferred acyl substrates (e.g. 4-substituted benzoates), in a magnesium ion- and ATP-dependent manner. Can use 4-substituted benzoates such as 4- aminobenzoate (pABA), 4-fluorobenzoate and 4-hydroxybenzoate (4- HBA), and, to a lesser extent, benzoate, vanillate and trans- cinnamate, but not 2-substituted benzoates and salicylic acid (SA), as conjugating acyl substrates. Involved in both basal and induced resistance in a SA-dependent manner. Confers resistance to virulent and avirulent pathogens (at least bacteria and oomycetes), and promotes SA glucosides accumulation. Required for the establishment of hyper-sensitive response (HR) upon incompatible interaction and subsequent systemic acquired resistance (SAR). |
| Lma22623.t1 | KTI1_ARATH | Exhibits Kunitz trypsin protease inhibitor activity. Involved in modulating programmed cell death (PCD) in plant- pathogen interactions. |
| Lma17738.t1 | IGMT1_ARATH | Involved in indole glucosinolate biosynthesis. Catalyzes methoxylation reactions of the glucosinolate indole ring. Converts the hydroxy intermediates 4-hydroxy-indol-3-yl-methylglucosinolate (4OH-I3M) and 1-hydroxy-indol-3-yl-methylglucosinolate (1OH-I3M) to 4-methoxy-indol-3-yl-methylglucosinolate (4MO-I3M) and 1- methoxy-indol-3-yl-methylglucosinolate (1MO-I3M), respectively. |
| Lma10813.t1 | POLR1_ARATH | NA |
| Lma12146.t1 | AGL80_ARATH | Probable transcription factor. Controls central cell differentiation during female gametophyte development. Required for the expression of DEMETER and DD46, but not for the expression of FIS2. Probable transcription factor that may function in the maintenance of the proper function of the central cell in pollen tube attraction (Probable). |
| Lma25676.t1 | KTI1_ARATH | Exhibits Kunitz trypsin protease inhibitor activity. Involved in modulating programmed cell death (PCD) in plant- pathogen interactions. |
| Lma18993.t1 | THAH_ARATH | Hydroxylates thalianol into thalian-diol. |
| Lma05303.t1 | MCC16_ARATH | NA |
| Lma14647.t1 | M310_ARATH | NA |
| Lma18660.t1 | GH312_ARATH | Catalyzes the conjugation of specific amino acids (e.g. Glu and possibly His, Lys, and Met) to their preferred acyl substrates (e.g. 4-substituted benzoates), in a magnesium ion- and ATP-dependent manner. Can use 4-substituted benzoates such as 4- aminobenzoate (pABA), 4-fluorobenzoate and 4-hydroxybenzoate (4- HBA), and, to a lesser extent, benzoate, vanillate and trans- cinnamate, but not 2-substituted benzoates and salicylic acid (SA), as conjugating acyl substrates. Involved in both basal and induced resistance in a SA-dependent manner. Confers resistance to virulent and avirulent pathogens (at least bacteria and oomycetes), and promotes SA glucosides accumulation. Required for the establishment of hyper-sensitive response (HR) upon incompatible interaction and subsequent systemic acquired resistance (SAR). |
| Lma04490.t1 | GH312_ARATH | Catalyzes the conjugation of specific amino acids (e.g. Glu and possibly His, Lys, and Met) to their preferred acyl substrates (e.g. 4-substituted benzoates), in a magnesium ion- and ATP-dependent manner. Can use 4-substituted benzoates such as 4- aminobenzoate (pABA), 4-fluorobenzoate and 4-hydroxybenzoate (4- HBA), and, to a lesser extent, benzoate, vanillate and trans- cinnamate, but not 2-substituted benzoates and salicylic acid (SA), as conjugating acyl substrates. Involved in both basal and induced resistance in a SA-dependent manner. Confers resistance to virulent and avirulent pathogens (at least bacteria and oomycetes), and promotes SA glucosides accumulation. Required for the establishment of hyper-sensitive response (HR) upon incompatible interaction and subsequent systemic acquired resistance (SAR). |
| Lma09213.t1 | ALP1_ARATH | Transposase-derived protein that may have nuclease activity (Probable). Antagonist of polycomb-group (PcG) protein- mediated chromatin silencing, probably by preventing the association of POLYCOMB REPRESSIVE COMPLEX 2 (PRC2) with its accessory components. Needed for full reactivation of several floral homeotic genes that are repressed by PcG. |
| Lma04405.t1 | KTI1_ARATH | Exhibits Kunitz trypsin protease inhibitor activity. Involved in modulating programmed cell death (PCD) in plant- pathogen interactions. |
| Lma03770.t1 | BAHD1_ARATH | Probably involved in the modification of desaturated thalian-diol. |
| Lma09872.t1 | M310_ARATH | NA |
| Lma03663.t1 | POLR1_ARATH | NA |
| Lma11572.t1 | GRP17_ARATH | Lipid-binding oleosin pollen coat protein required to mediate pollen recognition by stigma cells and subsequent pollen hydration. |
| Lma15115.t1 | POLX_TOBAC | NA |
| Lma06906.t1 | POLX_TOBAC | NA |
| Lma03729.t1 | MAIN_ARATH | Required for the organization of the root apical meristem (RAM) and the shoot apical meristem (SAM). Required to maintain genome stability and cell division activity in meristematic cells. |
| Lma18495.t1 | BAHD2_ARATH | NA |
| Lma08002.t1 | MCC13_ARATH | NA |
| Lma02867.t1 | POLX_TOBAC | NA |
| Lma01101.t1 | POLX_TOBAC | NA |
| Lma22917.t1 | IGMT1_ARATH | Involved in indole glucosinolate biosynthesis. Catalyzes methoxylation reactions of the glucosinolate indole ring. Converts the hydroxy intermediates 4-hydroxy-indol-3-yl-methylglucosinolate (4OH-I3M) and 1-hydroxy-indol-3-yl-methylglucosinolate (1OH-I3M) to 4-methoxy-indol-3-yl-methylglucosinolate (4MO-I3M) and 1- methoxy-indol-3-yl-methylglucosinolate (1MO-I3M), respectively. |
| Lma14895.t1 | WRK10_ARATH | Transcription factor. Interacts specifically with the W box (5'-(T)TGAC[CT]-3'), a frequently occurring elicitor- responsive cis-acting element (By similarity). Modulates seed size by negatively regulating the cellularization of syncytial endosperm. |
| Lma20359.t1 | MCC31_ARATH | NA |
| Lma16116.t1 | Y4200_ARATH | NA |
| Lma23151.t1 | THAH_ARATH | Hydroxylates thalianol into thalian-diol. |
| Lma18996.t1 | BIA1_ARATH | Monitors brassinosteroids (BR) responses and homeostasis, particularly in the root and hypocotyl in darkness. Promotes flavonoid biosynthesis. |
| Lma23149.t1 | PEN7_ARATH | NA |
| Lma21740.t1 | EFR_ARATH | Constitutes the pattern-recognition receptor (PPR) that determines the specific perception of elongation factor Tu (EF- Tu), a potent elicitor of the defense response to pathogen- associated molecular patterns (PAMPs). Reduces transformation by Rhizobium radiobacter probably by inducing plant defense during the interaction. Binding to the effector AvrPto1 from P.syringae blocks the downstream plant immune response while interaction with hopD2 decreases the phosphorylation level of EFR upon elf18 treatment. Specific endoplasmic reticulum quality control components (ERD2B, CRT3, UGGT and STT3A) are required for the biogenesis of EFR. |
| Lma25925.t1 | M310_ARATH | NA |
| Lma07535.t1 | THAH_ARATH | Hydroxylates thalianol into thalian-diol. |
| Lma25359.t1 | PP339_ARATH | NA |
| Lma21478.t1 | M310_ARATH | NA |
| Lma08006.t1 | RTM3_ARATH | Required for the restriction of long-distance movement of the pathogenic tobacco etch virus (TEV) without causing a hypersensitive response or inducing systemic acquired resistance. |
| Lma17683.t1 | PPR91_ARATH | NA |
| Lma03256.t1 | PHE1_ARATH | Probable transcription factor involved in the development of gametophytes and seeds. |
| Lma20036.t1 | Y3475_ARATH | NA |
| Lma21829.t1 | PP105_ARATH | NA |
| Lma10881.t1 | FB181_ARATH | NA |
| Lma20933.t1 | POLR1_ARATH | NA |
| Lma23156.t1 | THAH_ARATH | Hydroxylates thalianol into thalian-diol. |
| Lma25736.t1 | POLX_TOBAC | NA |
| Lma04228.t1 | IGMT1_ARATH | Involved in indole glucosinolate biosynthesis. Catalyzes methoxylation reactions of the glucosinolate indole ring. Converts the hydroxy intermediates 4-hydroxy-indol-3-yl-methylglucosinolate (4OH-I3M) and 1-hydroxy-indol-3-yl-methylglucosinolate (1OH-I3M) to 4-methoxy-indol-3-yl-methylglucosinolate (4MO-I3M) and 1- methoxy-indol-3-yl-methylglucosinolate (1MO-I3M), respectively. |
| Lma17748.t1 | PPR99_ARATH | NA |
| Lma17273.t1 | ALPL_ARATH | Transposase-derived protein that may have nuclease activity. |
| Lma17749.t1 | PPR99_ARATH | NA |
| Lma20353.t1 | RTM3_ARATH | Required for the restriction of long-distance movement of the pathogenic tobacco etch virus (TEV) without causing a hypersensitive response or inducing systemic acquired resistance. |
| Lma17687.t1 | PPR99_ARATH | NA |
| Lma15159.t1 | M310_ARATH | NA |
| Lma23061.t1 | ALPL_ARATH | Transposase-derived protein that may have nuclease activity. |
| Lma11573.t1 | GRP17_ARATH | Lipid-binding oleosin pollen coat protein required to mediate pollen recognition by stigma cells and subsequent pollen hydration. |
| Lma08003.t1 | MCC17_ARATH | NA |
| Lma24612.t1 | GH312_ARATH | Catalyzes the conjugation of specific amino acids (e.g. Glu and possibly His, Lys, and Met) to their preferred acyl substrates (e.g. 4-substituted benzoates), in a magnesium ion- and ATP-dependent manner. Can use 4-substituted benzoates such as 4- aminobenzoate (pABA), 4-fluorobenzoate and 4-hydroxybenzoate (4- HBA), and, to a lesser extent, benzoate, vanillate and trans- cinnamate, but not 2-substituted benzoates and salicylic acid (SA), as conjugating acyl substrates. Involved in both basal and induced resistance in a SA-dependent manner. Confers resistance to virulent and avirulent pathogens (at least bacteria and oomycetes), and promotes SA glucosides accumulation. Required for the establishment of hyper-sensitive response (HR) upon incompatible interaction and subsequent systemic acquired resistance (SAR). |
| Lma15095.t1 | BIA1_ARATH | Monitors brassinosteroids (BR) responses and homeostasis, particularly in the root and hypocotyl in darkness. Promotes flavonoid biosynthesis. |
| Lma05302.t1 | MCC16_ARATH | NA |
| Lma19040.t1 | GRP17_ARATH | Lipid-binding oleosin pollen coat protein required to mediate pollen recognition by stigma cells and subsequent pollen hydration. |
| Lma23150.t1 | BIA1_ARATH | Monitors brassinosteroids (BR) responses and homeostasis, particularly in the root and hypocotyl in darkness. Promotes flavonoid biosynthesis. |
| Lma17750.t1 | PPR99_ARATH | NA |
| Lma08419.t1 | FB181_ARATH | NA |
| Lma24376.t1 | POLR1_ARATH | NA |
| Lma04229.t1 | IGMT1_ARATH | Involved in indole glucosinolate biosynthesis. Catalyzes methoxylation reactions of the glucosinolate indole ring. Converts the hydroxy intermediates 4-hydroxy-indol-3-yl-methylglucosinolate (4OH-I3M) and 1-hydroxy-indol-3-yl-methylglucosinolate (1OH-I3M) to 4-methoxy-indol-3-yl-methylglucosinolate (4MO-I3M) and 1- methoxy-indol-3-yl-methylglucosinolate (1MO-I3M), respectively. |

**Supplementary Table 14. PSGs with function description in Swiss-prot database*.***

| **Gene ID** | **Swiss-Prot Annotation** | **Gene function description** |
| --- | --- | --- |
| Lma02126.t1 | CHX23_ARATH | Operates as a K(+)/H(+) antiporter or Na(+)/H(+) antiporter of the chloroplast envelope that functions in pH homeostasis and chloroplast development. Monovalent cation transporter with a preference for Cs(+), K(+) and Rb(+) relative to Na(+) or Li(+). Required for pollen tube guidance, but not for normal pollen development. May also be involved in the development or function of the female gametophyte. |
| Lma02857.t1 | BGAL8_ARATH | NA |
| Lma05374.t1 | RLA32_ARATH | Plays an important role in the elongation step of protein synthesis. |
| Lma20722.t1 | SC35_ARATH | Probably involved in intron recognition and spliceosome assembly, but not involved in alternative splicing regulation of the SCL33 intron. |
| Lma22470.t1 | VP52A_ARATH | Acts as component of the GARP complex that is involved in retrograde transport from early and late endosomes to the trans-Golgi network (TGN). The GARP complex facilitates tethering as well as SNARE complex assembly at the Golgi (By similarity). Required for pollen tube elongation and other polar growth. |
| Lma08626.t1 | AHL23_ARATH | Transcription factor that specifically binds AT-rich DNA sequences related to the nuclear matrix attachment regions (MARs). . |
| Lma02976.t1 | PTR27_ARATH | Low-affinity proton-dependent nitrate transporter. Not involved in dipeptides transport. |
| Lma03452.t1 | CPL1_ARATH | Processively dephosphorylates 'Ser-5' but not 'Ser-2' of the heptad repeats YSPTSPS in the C-terminal domain of the largest RNA polymerase II subunit (RPB1). This promotes the activity of RNA polymerase II. Together with CPL2, required for male gametes fertility. Multifunctional regulator that modulates plant growth, stress, and phytohormones responses. Negative regulator of stress gene transcription involved in abscisic acid (ABA) mediated and jasmonic acid (JA) mediated signaling pathways, NaCl, osmotic stress, wounding, and cold resistance. Regulates negatively the expression of jasmonic acid (JA) biosynthetic genes in response to wounding . Forms a complex with RCF3 that modulates co-transcriptional processes such as mRNA capping and polyadenylation, and functions to repress stress-inducible gene expression . Dephosphorylates RCF3 . Involved in the dephosphorylation of EIF4A3. This dephosphorylation retains EIF4A3 in the nucleus and limits its accumulation in the cytoplasm. Is essential for the degradation of the nonsense-mediated mRNA decay (NMD) transcripts. |
| Lma17812.t1 | PMEI_ACTDE | Inhibits pectin methylesterase; may be involved in the regulation of fruit ripening. |
| Lma21436.t1 | SGT1B_ARATH | Involved in plant innate immunity. Is essential for resistance conferred by multiple R genes recognizing different oomycete pathogen isolates like avirulent H.parasitica (downy mildew). Contributes additively with RAR1 to RPP5-dependent resistance. Not required for RPM1, RPS2, RPS4 and RPS5-mediated resistance. Functions as negative regulator of RPS5 accumulation by assisting its degradation. May be involved in heat shock response by associating with HSC70-1 chaperone. Required for the SCF(TIR1)-mediated degradation of Aux/IAA proteins, but maybe not for SCF(TIR1) assembly or binding to its Aux/IAA substrates. Probably required for SCF-mediated ubiquitination, by coupling HSP90 to SCF complex for ubiquitination of HSP90 client proteins. Required for the coronatine/jasmonic acid-mediated signal transduction pathway. |
| Lma09083.t2 | ALAT2_ARATH | NA |
| Lma12783.t1 | STP2_ARATH | Mediates an active uptake of hexoses, probably by sugar/hydrogen symport. Can transport glucose, 3-O-methylglucose, xylose, mannose, fructose and galactose. |
| Lma00860.t1 | GIL1_ARATH | Required for red (R) and far red (FR) light-induced and phytochrome-mediated deregulation of negative gravitropism leading to randomization of hypocotyl growth orientation. |
| Lma12329.t1 | DMC1_ARATH | May participate in meiotic recombination, specifically in homologous strand assimilation, which is required for the resolution of meiotic double-strand breaks . Mediates interhomolog recombination during meiosis . |
| Lma15075.t1 | KASM_ARATH | Catalyzes all the condensation reaction of fatty acid synthesis by the addition to an acyl acceptor of two carbons from malonyl-ACP. Able to elongate saturated acyl chains from 4 to at least 16 carbons. Uses malonyl-CoA but not acetyl-CoA as primer substrate. When expressed in a heterologous system, reveals a bimodal distribution of products, with peaks at C8 and C14-C16. The major product of the reaction (octanoyl-ACP) is required for the lipoylation of essential mitochondrial proteins. |
| Lma04757.t1 | PNSB2_ARATH | NDH shuttles electrons from NAD(P)H:plastoquinone, via FMN and iron-sulfur (Fe-S) centers, to quinones in the photosynthetic chain and possibly in a chloroplast respiratory chain. The immediate electron acceptor for the enzyme in this species is believed to be plastoquinone. Couples the redox reaction to proton translocation, and thus conserves the redox energy in a proton gradient. |
| Lma09821.t1 | PYRP2_ARATH | Catalyzes the dephosphorylation of 5-amino-6-(5-phospho- D-ribitylamino)uracil, also known as ARPP, but has no activity toward flavin mononucleotide (FMN) . |
| Lma17801.t1 | OLA1_ARATH | Hydrolyzes ATP, and can also hydrolyze GTP with lower efficiency. Has lower affinity for GTP (Potential). Exhibits GTPase activity (By similarity). Confers sensitivity to salinity stress by suppressing the anti-oxidation enzymatic activities and increasing lipid peroxidation thus leading to the accumulation of reactive oxygen species (ROS) . Acts as negative regulator of disease resistance against bacterial pathogen . |
| Lma14805.t1 | TRXB2_ARATH | Possesses thioredoxin-disulfide reductase activity towards thioredoxins O1, O2 and F3. |
| Lma05841.t1 | HP302_ARATH | Together with HP30-1 and HP20, triggers the import and insertion of transit sequence-less multi-pass transmembrane proteins (e.g. CEQORH) into the chloroplastic inner membrane. |
| Lma15462.t1 | RH2_ARATH | ATP-dependent RNA helicase. Core component of the splicing-dependent multiprotein exon junction complex (EJC) deposited at splice junctions on mRNAs. The EJC is a dynamic structure consisting of core proteins and several peripheral nuclear and cytoplasmic associated factors that join the complex only transiently either during EJC assembly or during subsequent mRNA metabolism. The EJC marks the position of the exon-exon junction in the mature mRNA for the gene expression machinery and the core components remain bound to spliced mRNAs throughout all stages of mRNA metabolism thereby influencing downstream processes including nuclear mRNA export, subcellular mRNA localization, translation efficiency and nonsense-mediated mRNA decay (NMD). Its RNA-dependent ATPase and RNA-helicase activities are induced by MLN51/CASC3, but abolished in presence of the MAGO-Y14 heterodimer, thereby trapping the ATP-bound EJC core onto spliced mRNA in a stable conformation. The inhibition of ATPase activity by the MAGO-Y14 heterodimer increases the RNA-binding affinity of the EJC (By similarity). Plays a role in abiotic stress adaptation. Can regulate abiotic stress resistance partially via the control of acetoacetyl-CoA thiolase 2 (AC Q8S4Y1) expression . |
| Lma22794.t1 | SAU50_ARATH | Effector of hormonal and environmental signals in plant growth. |
| Lma04193.t1 | FB91_ARATH | Component of SCF(ASK-cullin-F-box) E3 ubiquitin ligase complexes, which may mediate the ubiquitination and subsequent proteasomal degradation of target proteins. |
| Lma21117.t1 | DRL29_ARATH | Probable disease resistance protein. |
| Lma23041.t1 | WRK40_ARATH | Transcription factor (By similarity). Interacts specifically with the W box (5'-(T)TGAC[CT]-3'), a frequently occurring elicitor-responsive cis-acting element (By similarity). |
| Lma17033.t1 | PXL2C_ARATH | NA |
| Lma18774.t1 | MD19A_ARATH | Component of the Mediator complex, a coactivator involved in the regulated transcription of nearly all RNA polymerase II-dependent genes. Mediator functions as a bridge to convey information from gene-specific regulatory proteins to the basal RNA polymerase II transcription machinery. The Mediator complex, having a compact conformation in its free form, is recruited to promoters by direct interactions with regulatory proteins and serves for the assembly of a functional preinitiation complex with RNA polymerase II and the general transcription factors. |
| Lma16374.t1 | PP211_ARATH | NA |
| Lma03423.t1 | AIR1_ARATH | NA |
| Lma06052.t1 | Y3037_ARATH | NA |
| Lma21347.t1 | ATPD_ARATH | F(1)F(0) ATP synthase produces ATP from ADP in the presence of a proton or sodium gradient. F-type ATPases consist of two structural domains, F(1) containing the extramembraneous catalytic core and F(0) containing the membrane proton channel, linked together by a central stalk and a peripheral stalk. During catalysis, ATP synthesis in the catalytic domain of F(1) is coupled via a rotary mechanism of the central stalk subunits to proton translocation (Potential). Essential for photosynthesis, probably by facilitating electron transport in both photosystems I and II . . |
| Lma09415.t1 | GT14_ARATH | Functions in xyloglucan synthesis by adding side chains to the xylosylated glucan backbone. Involved in the galactosylation of hemicellulose xyloglucan. |
| Lma16071.t1 | PAH1_ARATH | Magnesium-dependent phosphatidate phosphatase which catalyzes the dephosphorylation of phosphatidate to yield diacylglycerol. Acts redundantly with PAH2 to repress phospholipid biosynthesis at the endoplasmic reticulum (ER). May function indirectly as repressor of multiple enzymes involved in phospholipid biosynthesis. Is involved in the pathway of galactolipid synthesis in the ER, which is required for the membrane lipid remodeling, an essential adaptation mechanism to cope with phosphate starvation. |
| Lma07143.t1 | DMP8_ARATH | Involved in membrane remodeling. |
| Lma20156.t1 | P2C48_ARATH | NA |
| Lma20387.t1 | SAD2H_ARATH | Functions probably in nuclear protein import, either by acting as autonomous nuclear transport receptor or as an adapter- like protein in association with other importin subunits. . |
| Lma20991.t1 | NRPB1_ARATH | DNA-dependent RNA polymerase catalyzes the transcription of DNA into RNA using the four ribonucleoside triphosphates as substrates. Largest and catalytic component of RNA polymerase II which synthesizes mRNA precursors and many functional non-coding RNAs. Forms the polymerase active center together with the second largest subunit. Pol II is the central component of the basal RNA polymerase II transcription machinery. It is composed of mobile elements that move relative to each other. NRPB1 is part of the core element with the central large cleft, the clamp element that moves to open and close the cleft and the jaws that are thought to grab the incoming DNA template. At the start of transcription, a single-stranded DNA template strand of the promoter is positioned within the central active site cleft of Pol II. A bridging helix emanates from NRPB1 and crosses the cleft near the catalytic site and is thought to promote translocation of Pol II by acting as a ratchet that moves the RNA-DNA hybrid through the active site by switching from straight to bent conformations at each step of nucleotide addition. During transcription elongation, Pol II moves on the template as the transcript elongates. Elongation is influenced by the phosphorylation status of the C-terminal domain (CTD) of Pol II largest subunit (NRPB1), which serves as a platform for assembly of factors that regulate transcription initiation, elongation, termination and mRNA processing. |
