# Supplementary material for: A chromosome-scale reference genome of Lobularia maritima, an ornamental plant with high stress tolerance
Source: Hortic Res. 2020 Dec 1;7:197. doi: 10.1038/s41438-020-00422-w (PMC7705659; doi:10.1038/s41438-020-00422-w)
Supplement: Supplementary file 3 — Supplementary table 11 [file 41438_2020_422_MOESM3_ESM.pdf]

Supplementary Table 11. Function of *L. maritima* specific genes with homolog in Swiss-prot database.

Gene ID	Swiss-Prot Annotatior	Gene Function
Lma12953.t1	ACFR1_ARATH	Two-heme-containing cytochrome. Catalyzes ascorbate- dependent trans-membrane ferric-chelate reduction. Able to use dihydrolipoic acid (DHLA) as an alternative substrate to ascorbate.
Lma16848.t1	RLF4_ARATH	Cell signaling peptide that may regulate plant stress, growth, and development. Mediates a rapid alkalinization of extracellular space by mediating a transient increase in the cytoplasmic Ca(2+) concentration leading to a calcium-dependent signaling events through a cell surface receptor and a concomitant activation of some intracellular mitogen-activated protein kinases (By similarity).
Lma17380.t1	ALEU_ARATH	May play a role in proteolysis leading to mobilization of nitrogen during senescence and starvation.
Lma22861.t1	DNJ10_ARATH	Have a continuous role in plant development probably in the structural organization of compartments.
Lma00592.t1	ASY2_ARATH	Required for normal meiosis.
Lma10446.t1	ASY2_ARATH	Required for normal meiosis.
Lma25849.t1	ASY2_ARATH	Required for normal meiosis.
Lma14963.t1	RFA1B_ARATH	Component of the replication protein A complex (RPA) required for DNA recombination, repair and replication. The activity of RPA is mediated by single-stranded DNA binding and protein interactions (By similarity). Probably involved in repair of double-strand DNA breaks (DSBs) induced by genotoxic stresses (By similarity).
Lma17596.t1	RFA1B_ARATH	Component of the replication protein A complex (RPA) required for DNA recombination, repair and replication. The activity of RPA is mediated by single-stranded DNA binding and protein interactions (By similarity). Probably involved in repair of double-strand DNA breaks (DSBs) induced by genotoxic stresses (By similarity).

Lma00535.t1	SINL7_ARATH	E3 ubiquitin-protein ligase that mediates ubiquitination and subsequent proteasomal degradation of target proteins. E3 ubiquitin ligases accept ubiquitin from an E2 ubiquitin- conjugating enzyme in the form of a thioester and then directly transfers the ubiquitin to targeted substrates. It probably triggers the ubiquitin-mediated degradation of different substrates.
Lma06240.t1	SINL7_ARATH	E3 ubiquitin-protein ligase that mediates ubiquitination and subsequent proteasomal degradation of target proteins. E3 ubiquitin ligases accept ubiquitin from an E2 ubiquitin- conjugating enzyme in the form of a thioester and then directly transfers the ubiquitin to targeted substrates. It probably triggers the ubiquitin-mediated degradation of different substrates.
Lma19508.t1	SINL7_ARATH	E3 ubiquitin-protein ligase that mediates ubiquitination and subsequent proteasomal degradation of target proteins. E3 ubiquitin ligases accept ubiquitin from an E2 ubiquitin- conjugating enzyme in the form of a thioester and then directly transfers the ubiquitin to targeted substrates. It probably triggers the ubiquitin-mediated degradation of different substrates.
Lma19569.t1	SINL7_ARATH	E3 ubiquitin-protein ligase that mediates ubiquitination and subsequent proteasomal degradation of target proteins. E3 ubiquitin ligases accept ubiquitin from an E2 ubiquitin- conjugating enzyme in the form of a thioester and then directly transfers the ubiquitin to targeted substrates. It probably triggers the ubiquitin-mediated degradation of different substrates.
Lma24260.t1	SINL7_ARATH	E3 ubiquitin-protein ligase that mediates ubiquitination and subsequent proteasomal degradation of target proteins. E3 ubiquitin ligases accept ubiquitin from an E2 ubiquitin- conjugating enzyme in the form of a thioester and then directly transfers the ubiquitin to targeted substrates. It probably triggers the ubiquitin-mediated degradation of different substrates.
Lma24392.t1	SINL9_ARATH	E3 ubiquitin-protein ligase that mediates ubiquitination and subsequent proteasomal degradation of target proteins. E3 ubiquitin ligases accept ubiquitin from an E2 ubiquitin- conjugating enzyme in the form of a thioester and then directly transfers the ubiquitin to targeted substrates. It probably triggers the ubiquitin-mediated degradation of different substrates.

Lma10414.t1	PMTT_ARATH	May be involved in the synthesis of homogalacturonan. Required for normal cell adhesion and plant development.
Lma10861.t1	PMTT_ARATH	May be involved in the synthesis of homogalacturonan. Required for normal cell adhesion and plant development.
Lma12584.t1	PMTT_ARATH	May be involved in the synthesis of homogalacturonan. Required for normal cell adhesion and plant development.
Lma25957.t1	PMTT_ARATH	May be involved in the synthesis of homogalacturonan. Required for normal cell adhesion and plant development.
Lma03871.t1	EA6_ARATH	Probable beta-1,3-glucanase that may be involved in the degradation of callose walls around the microspore tetrad during pollen development (Probable). May be required for pollen exine formation
Lma06492.t1	EA6_ARATH	Probable beta-1,3-glucanase that may be involved in the degradation of callose walls around the microspore tetrad during pollen development (Probable). May be required for pollen exine formation
Lma09994.t1	EA6_ARATH	Probable beta-1,3-glucanase that may be involved in the degradation of callose walls around the microspore tetrad during pollen development (Probable). May be required for pollen exine formation
Lma12299.t1	EA6_ARATH	Probable beta-1,3-glucanase that may be involved in the degradation of callose walls around the microspore tetrad during pollen development (Probable). May be required for pollen exine formation
Lma25457.t1	EA6_ARATH	Probable beta-1,3-glucanase that may be involved in the degradation of callose walls around the microspore tetrad during pollen development (Probable). May be required for pollen exine formation
Lma10690.t1	XCP2_ARATH	Cysteine protease involved in xylem tracheary element (TE) autolysis during xylogenesis in roots. Participates in micro autolysis within the intact central vacuole before mega autolysis is initiated by tonoplast implosionInvolved in susceptibility to the bacterial plant pathogen Ralstonia solanacearum
Lma14957.t1	XCP2_ARATH	Cysteine protease involved in xylem tracheary element (TE) autolysis during xylogenesis in roots. Participates in micro autolysis within the intact central vacuole before mega autolysis is initiated by tonoplast implosionInvolved in susceptibility to the bacterial plant pathogen Ralstonia solanacearum
Lma14986.t1	XCP2_ARATH	Cysteine protease involved in xylem tracheary element (TE) autolysis during xylogenesis in roots. Participates in micro autolysis within the intact central vacuole before mega autolysis is initiated by tonoplast implosionInvolved in susceptibility to the bacterial plant pathogen Ralstonia solanacearum
Lma18192.t1	TF2B2_ARATH	General factor that plays a major role in the activation of eukaryotic genes transcribed by RNA polymerase II.

Lma20293.t1	TF2B_SOYBN	General factor that plays a major role in the activation of eukaryotic genes transcribed by RNA polymerase II.
Lma20294.t1	TF2B1_ARATH	General factor that plays a major role in the activation of eukaryotic genes transcribed by RNA polymerase II (By similarity). Interacts with TBP2 and is required for activated transcription and possibly basal transcriptionPlays important roles in pollen tube growth, guidance, and reception as well as endosperm development. Is partially functionally different from TFIIB2 and PBRP2
Lma20295.t1	TF2B1_ARATH	General factor that plays a major role in the activation of eukaryotic genes transcribed by RNA polymerase II (By similarity). Interacts with TBP2 and is required for activated transcription and possibly basal transcriptionPlays important roles in pollen tube growth, guidance, and reception as well as endosperm development. Is partially functionally different from TFIIB2 and PBRP2
Lma20296.t1	TF2B1_ARATH	General factor that plays a major role in the activation of eukaryotic genes transcribed by RNA polymerase II (By similarity). Interacts with TBP2 and is required for activated transcription and possibly basal transcriptionPlays important roles in pollen tube growth, guidance, and reception as well as endosperm development. Is partially functionally different from TFIIB2 and PBRP2
Lma00469.t1	RPS6R_ARATH	Disease resistance (R) protein that specifically recognizes the hopA1 type III effector avirulence protein from Pseudomonas syringae. Resistance proteins guard the plant against pathogens that contain an appropriate avirulence protein via an indirect interaction with this avirulence protein. That triggers a defense system including the hypersensitive response, which restricts the pathogen growth.
Lma17365.t1	RPS6C_ARATH	Disease resistance (R) protein that specifically recognizes the hopA1 type III effector avirulence protein from Pseudomonas syringae. Resistance proteins guard the plant against pathogens that contain an appropriate avirulence protein via an indirect interaction with this avirulence protein. That triggers a defense system including the hypersensitive response, which restricts the pathogen growth.
Lma21707.t1	VICTR_ARATH	Disease resistance protein of the TIR-NB-LRR-type. Part of the RPS6 locus that contains a cluster of several paralogous disease resistance (R) genes. Resistance proteins guard the plant against pathogens that contain an appropriate avirulence protein via an indirect interaction with this avirulence protein. That triggers a defense system including the hypersensitive response, which restricts the pathogen growth (By similarity). Required for [5-(3,4-dichlorophenyl)furan-2-yl]-piperidine-1-ylmethanethione- (DFPM-) induced root growth arrest due to reduced number of meristem cells in the division zone of the primary root and inhibition of abscisic acid- (ABA-) induced stomatal closing.

Lma21709.t1	VICTR_ARATH	Disease resistance protein of the TIR-NB-LRR-type. Part of the RPS6 locus that contains a cluster of several paralogous disease resistance (R) genes. Resistance proteins guard the plant against pathogens that contain an appropriate avirulence protein via an indirect interaction with this avirulence protein. That triggers a defense system including the hypersensitive response, which restricts the pathogen growth (By similarity). Required for [5-(3,4-dichlorophenyl)furan-2-yl]-piperidine-1-ylmethanethione- (DFPM-) induced root growth arrest due to reduced number of meristem cells in the division zone of the primary root and inhibition of abscisic acid- (ABA-) induced stomatal closing.
Lma21710.t1	VICTR_ARATH	Disease resistance protein of the TIR-NB-LRR-type. Part of the RPS6 locus that contains a cluster of several paralogous disease resistance (R) genes. Resistance proteins guard the plant against pathogens that contain an appropriate avirulence protein via an indirect interaction with this avirulence protein. That triggers a defense system including the hypersensitive response, which restricts the pathogen growth (By similarity). Required for [5-(3,4-dichlorophenyl)furan-2-yl]-piperidine-1-ylmethanethione- (DFPM-) induced root growth arrest due to reduced number of meristem cells in the division zone of the primary root and inhibition of abscisic acid- (ABA-) induced stomatal closing.
Lma00536.t1	UBC11_ARATH	Accepts the ubiquitin from the E1 complex and catalyzes its covalent attachment to other proteins. Mediates the selective degradation of short-lived and abnormal proteins.
Lma06241.t1	UBC11_ARATH	Accepts the ubiquitin from the E1 complex and catalyzes its covalent attachment to other proteins. Mediates the selective degradation of short-lived and abnormal proteins.
Lma06273.t1	UBC11_ARATH	Accepts the ubiquitin from the E1 complex and catalyzes its covalent attachment to other proteins. Mediates the selective degradation of short-lived and abnormal proteins.
Lma24259.t1	UBC11_ARATH	Accepts the ubiquitin from the E1 complex and catalyzes its covalent attachment to other proteins. Mediates the selective degradation of short-lived and abnormal proteins.
Lma24393.t1	UBC11_ARATH	Accepts the ubiquitin from the E1 complex and catalyzes its covalent attachment to other proteins. Mediates the selective degradation of short-lived and abnormal proteins.
Lma05158.t1	ASY2_ARATH	Required for normal meiosis.
Lma05162.t1	ASY2_ARATH	Required for normal meiosis.
Lma08617.t1	CYT4_ARATH	Specific inhibitor of cysteine proteinases. Probably involved in the regulation of endogenous processes and in defense against pests and pathogens (By similarity).
Lma08618.t1	CYT4_ARATH	Specific inhibitor of cysteine proteinases. Probably involved in the regulation of endogenous processes and in defense against pests and pathogens (By similarity).

Lma08619.t1	CYT4_ARATH	Specific inhibitor of cysteine proteinases. Probably involved in the regulation of endogenous processes and in defense against pests and pathogens (By similarity).
Lma24225.t1	CYT4_ARATH	Specific inhibitor of cysteine proteinases. Probably involved in the regulation of endogenous processes and in defense against pests and pathogens (By similarity).
Lma03430.t1	3AT1_ARATH	Involved in the acylation of the 6" position of the 3- O-glucose residue of anthocyanin. Also able to use flavonol 3- glucosides as the acyl acceptor.
Lma05002.t1	3AT1_ARATH	Involved in the acylation of the 6" position of the 3- O-glucose residue of anthocyanin. Also able to use flavonol 3- glucosides as the acyl acceptor.
Lma06935.t1	3AT1_ARATH	Involved in the acylation of the 6" position of the 3- O-glucose residue of anthocyanin. Also able to use flavonol 3- glucosides as the acyl acceptor.
Lma26149.t1	3AT1_ARATH	Involved in the acylation of the 6" position of the 3- O-glucose residue of anthocyanin. Also able to use flavonol 3- glucosides as the acyl acceptor.
Lma00172.t1	MIA40_ARATH	Required for the import and folding of small cysteine- containing proteins in the mitochondrial intermembrane space (Probable). Involved in the mitochondrial oxidative folding of the copper-zinc superoxide dismutase CSD1, the copper chaperone for superoxide dismutase CCS, and subunits of the mitochondrial membrane respiratory chain NADH dehydrogenase (Complex I). Involved in the peroxisomal oxidative folding of the copper-zinc superoxide dismutase CSD3, and the fatty acid beta-oxidation multifunctional protein AIM1
Lma00191.t1	MIA40_ARATH	Required for the import and folding of small cysteine- containing proteins in the mitochondrial intermembrane space (Probable). Involved in the mitochondrial oxidative folding of the copper-zinc superoxide dismutase CSD1, the copper chaperone for superoxide dismutase CCS, and subunits of the mitochondrial membrane respiratory chain NADH dehydrogenase (Complex I). Involved in the peroxisomal oxidative folding of the copper-zinc superoxide dismutase CSD3, and the fatty acid beta-oxidation multifunctional protein AIM1
Lma14406.t1	MIA40_ARATH	Required for the import and folding of small cysteine- containing proteins in the mitochondrial intermembrane space (Probable). Involved in the mitochondrial oxidative folding of the copper-zinc superoxide dismutase CSD1, the copper chaperone for superoxide dismutase CCS, and subunits of the mitochondrial membrane respiratory chain NADH dehydrogenase (Complex I). Involved in the peroxisomal oxidative folding of the copper-zinc superoxide dismutase CSD3, and the fatty acid beta-oxidation multifunctional protein AIM1

Lma19027.t1	MIA40_ARATH	Required for the import and folding of small cysteine- containing proteins in the mitochondrial intermembrane space (Probable). Involved in the mitochondrial oxidative folding of the copper-zinc superoxide dismutase CSD1, the copper chaperone for superoxide dismutase CCS, and subunits of the mitochondrial membrane respiratory chain NADH dehydrogenase (Complex I). Involved in the peroxisomal oxidative folding of the copper-zinc superoxide dismutase CSD3, and the fatty acid beta-oxidation multifunctional protein AIM1
Lma05267.t1	RTNLA_ARATH	Plays a role in the Agrobacterium-mediated plant transformation via its interaction with VirB2, the major component of the T-pilus.
Lma11002.t1	RTNLA_ARATH	Plays a role in the Agrobacterium-mediated plant transformation via its interaction with VirB2, the major component of the T-pilus.
Lma19575.t1	RTNLA_ARATH	Plays a role in the Agrobacterium-mediated plant transformation via its interaction with VirB2, the major component of the T-pilus.
Lma22096.t1	RTNLA_ARATH	Plays a role in the Agrobacterium-mediated plant transformation via its interaction with VirB2, the major component of the T-pilus.
Lma06374.t1	B3GT2_ARATH	Beta-1,3-galactosyltransferase that transfers galactose from UDP-galactose to substrates with a terminal glycosyl residue.
Lma10752.t1	B3GT2_ARATH	Beta-1,3-galactosyltransferase that transfers galactose from UDP-galactose to substrates with a terminal glycosyl residue.
Lma04667.t1	SMD1A_ARATH	Involved in splicing regulation. Facilitates post- transcriptional gene silencing (PTGS) by limiting the degradation of transgene aberrant RNAs by the RNA quality control (RQC) machinery, thus favoring their entry into cytoplasmic siRNA bodies where they can trigger PTGS. Does not participate in the production of small RNAs.
Lma10175.t1	SMD1A_ARATH	Involved in splicing regulation. Facilitates post- transcriptional gene silencing (PTGS) by limiting the degradation of transgene aberrant RNAs by the RNA quality control (RQC) machinery, thus favoring their entry into cytoplasmic siRNA bodies where they can trigger PTGS. Does not participate in the production of small RNAs.
Lma26191.t1	SMD1A_ARATH	Involved in splicing regulation. Facilitates post- transcriptional gene silencing (PTGS) by limiting the degradation of transgene aberrant RNAs by the RNA quality control (RQC) machinery, thus favoring their entry into cytoplasmic siRNA bodies where they can trigger PTGS. Does not participate in the production of small RNAs.

Lma00740.t1	CSN6B_ARATH	Component of the COP9 signalosome complex (CSN), a complex involved in various cellular and developmental processes such as photomorphogenesis and auxin and jasmonate responses. The CSN complex is an essential regulator of the ubiquitin (Ubl) conjugation pathway by mediating the deneddylation of the cullin subunits of SCF-type E3 ligase complexes, leading to decrease the Ubl ligase activity of SCF. It is involved in repression of photomorphogenesis in darkness by regulating the activity of COP1- containing Ubl ligase complexes. The complex is also required for degradation of PSIAA6 by regulating the activity of the Ubl ligase SCF-TIR complex. Essential for the structural integrity of the CSN holocomplex
Lma10101.t1	CSN6B_ARATH	Component of the COP9 signalosome complex (CSN), a complex involved in various cellular and developmental processes such as photomorphogenesis and auxin and jasmonate responses. The CSN complex is an essential regulator of the ubiquitin (Ubl) conjugation pathway by mediating the deneddylation of the cullin subunits of SCF-type E3 ligase complexes, leading to decrease the Ubl ligase activity of SCF. It is involved in repression of photomorphogenesis in darkness by regulating the activity of COP1- containing Ubl ligase complexes. The complex is also required for degradation of PSIAA6 by regulating the activity of the Ubl ligase SCF-TIR complex. Essential for the structural integrity of the CSN holocomplex
Lma10594.t1	ARI16_ARATH	Might act as an E3 ubiquitin-protein ligase, or as part of E3 complex, which accepts ubiquitin from specific E2 ubiquitin- conjugating enzymes and then transfers it to substrates.
Lma10595.t1	ARI16_ARATH	Might act as an E3 ubiquitin-protein ligase, or as part of E3 complex, which accepts ubiquitin from specific E2 ubiquitin- conjugating enzymes and then transfers it to substrates.
Lma02077.t1	PMD2_ARATH	Involved in morphogenesis and proliferation of mitochondria. Does not act redundantly with PMD1. Is not involved in peroxisomal proliferation.
Lma10622.t1	PMD2_ARATH	Involved in morphogenesis and proliferation of mitochondria. Does not act redundantly with PMD1. Is not involved in peroxisomal proliferation.
Lma17628.t1	PMD2_ARATH	Involved in morphogenesis and proliferation of mitochondria. Does not act redundantly with PMD1. Is not involved in peroxisomal proliferation.
Lma15071.t1	NB5R1_ARATH	Reductase transferring electrons from NADH to cytochrome b5. Required for the NADH-dependent electron transfer involved in the desaturation and hydroxylation of fatty acids and in the desaturation of sterol precursors. No activity with NADPH as electron donor.

Lma17553.t1	NB5R1_ARATH	Reductase transferring electrons from NADH to cytochrome b5. Required for the NADH-dependent electron transfer involved in the desaturation and hydroxylation of fatty acids and in the desaturation of sterol precursors. No activity with NADPH as electron donor.
Lma19698.t1	C3H19_ARATH	Plays a central role in integrating RNA silencing and chromatin signals in 21 nt siRNA-dependent DNA methylation on cytosine pathway leading to transcriptional gene silencing of specific sequences. Involved in a chromatin-based RNA silencing pathway that encompasses both post-transcriptional gene silencing (PTGS) (e.g. RDR1, RDR6 and AGO2) and transcriptional gene silencing (TGS) (e.g. siRNA-dependent DNA methylation and histone H3) components. Mediates siRNA accumulation at specific chromatin loci. Binds H3K4me0 through its PHD to enforce low levels of H3K4 methylation and gene silencing at a subset of genomic loci.
Lma14215.t1	SCP39_ARATH	Probable carboxypeptidase.
Lma14216.t1	SCP39_ARATH	Probable carboxypeptidase.
Lma20026.t1	SCP36_ARATH	Probable carboxypeptidase.
Lma04423.t1	TMVRN_NICGU	Disease resistance protein. Resistance proteins guard the plant against pathogens that contain an appropriate avirulence protein via a direct or indirect interaction with this avirulence protein. That triggers a defense system including the hypersensitive response, which restricts the pathogen growth.
Lma04424.t1	TIR_ARATH	Disease resistance protein. Resistance proteins guard the plant against pathogens that contain an appropriate avirulence protein via a direct or indirect interaction with this avirulence protein. That triggers a defense system including the hypersensitive response, which restricts the pathogen growth (By similarity).
Lma19833.t1	TMVRN_NICGU	Disease resistance protein. Resistance proteins guard the plant against pathogens that contain an appropriate avirulence protein via a direct or indirect interaction with this avirulence protein. That triggers a defense system including the hypersensitive response, which restricts the pathogen growth.
Lma17406.t1	FHY3_ARATH	Transcription activator that recognizes and binds to the DNA consensus sequence 5'-CACGCGC-3'. Activates the expression of FHY1 and FHL involved in light responses. When associated with PHYA, protects it from being recognized and degraded by the COP1/SPA complex. Positive regulator of chlorophyll biosynthesis via the activation of HEMB1 gene expression.
Lma20161.t1	FHY3_ARATH	Transcription activator that recognizes and binds to the DNA consensus sequence 5'-CACGCGC-3'. Activates the expression of FHY1 and FHL involved in light responses. When associated with PHYA, protects it from being recognized and degraded by the COP1/SPA complex. Positive regulator of chlorophyll biosynthesis via the activation of HEMB1 gene expression.

Lma25607.t1	FRS5_ARATH	Putative transcription activator involved in regulating light control of development.
Lma03847.t1	ASY2_ARATH	Required for normal meiosis.
Lma13315.t1	ASY2_ARATH	Required for normal meiosis.
Lma10801.t1	OEP80_ARATH	Plays an essential role during early stages of plastid development.
Lma15057.t1	OEP80_ARATH	Plays an essential role during early stages of plastid development.
Lma19432.t1	OEP80_ARATH	Plays an essential role during early stages of plastid development.
Lma05131.t1	ITPK2_ARATH	Kinase that can phosphorylate various inositol polyphosphate such as Ins(3,4,5,6)P4 or Ins(1,3,4)P3. Phosphorylates Ins(3,4,5,6)P4 to form InsP5This reaction is thought to have regulatory importance, since Ins(3,4,5,6)P4 is an inhibitor of plasma membrane Ca(2+)-activated Cl(-) channels, while Ins(1,3,4,5,6)P5 is not (By similarity). Also phosphorylates Ins(1,3,4)P3 or a racemic mixture of Ins(1,4,6)P3 and Ins(3,4,6)P3 to form InsP4Ins(1,3,4,6)P4 is an essential molecule in the hexakisphosphate (InsP6) pathway (By similarity). Plays a role in seed coat development and lipid polyester barrier formation
Lma14754.t1	THAH_ARATH	Hydroxylates thalianol into thalian-diol.
Lma13064.t1	BI1_ARATH	Suppressor of apoptosis. Modulator of endoplasmic reticulum stress-mediated programmed cell death. Involved in methyl jasmonate-induced leaf senescence through regulating cytoplasmic calcium level.
Lma22957.t1	RPP1_ARATH	TIR-NB-LRR receptor-like protein that confers resistance to the pathogen Hyaloperonospora arabidopsis.
Lma22958.t1	RPP1_ARATH	TIR-NB-LRR receptor-like protein that confers resistance to the pathogen Hyaloperonospora arabidopsis.
Lma00736.t1	CDPKD_ARATH	May play a role in signal transduction pathways that involve calcium as a second messenger.
Lma15146.t1	CDPKD_ARATH	May play a role in signal transduction pathways that involve calcium as a second messenger.
Lma24053.t1	CDPKD_ARATH	May play a role in signal transduction pathways that involve calcium as a second messenger.
Lma00575.t2	SWET8_ARATH	Mediates both low-affinity uptake and efflux of sugar across the plasma membrane. Required, in pollen, for microspore cell integrity and primexine pattern formation (,).
Lma14978.t2	SWET8_ARATH	Mediates both low-affinity uptake and efflux of sugar across the plasma membrane. Required, in pollen, for microspore cell integrity and primexine pattern formation (,).
Lma15042.t2	SWET8_ARATH	Mediates both low-affinity uptake and efflux of sugar across the plasma membrane. Required, in pollen, for microspore cell integrity and primexine pattern formation (,).

Lma07325.t1	LBD6_MAIZE	Promotes the switch from proliferation to differentiation in the embryo sac. Negative regulator of cell proliferation in the adaxial side of leaves. Regulates the formation of a symmetric lamina and the establishment of venation. Interacts directly with RS2 (rough sheath 2) to repress some knox homeobox genes.
Lma11770.t5	FIS1B_ARATH	Component of the peroxisomal and mitochondrial division machineries. Plays a role in promoting the fission of mitochondria and peroxisomes. In association with PEX11C, PEX11D, PEX11E and DRP3A, is involved in cell cycle-associated constitutive self- replication of preexisting peroxisomes.
Lma17635.t1	FIS1B_ARATH	Component of the peroxisomal and mitochondrial division machineries. Plays a role in promoting the fission of mitochondria and peroxisomes. In association with PEX11C, PEX11D, PEX11E and DRP3A, is involved in cell cycle-associated constitutive self- replication of preexisting peroxisomes.
Lma25970.t5	FIS1B_ARATH	Component of the peroxisomal and mitochondrial division machineries. Plays a role in promoting the fission of mitochondria and peroxisomes. In association with PEX11C, PEX11D, PEX11E and DRP3A, is involved in cell cycle-associated constitutive self- replication of preexisting peroxisomes.
Lma04858.t1	TBCC_ARATH	Essential tubulin-folding protein involved in the final step of the tubulin folding pathway. Required for continuous microtubule cytoskeleton organization, mitotic division, cytokinesis, and to couple cell cycle progression to cell division in embryos and endosperms. Not essential for cell viability. Binds probably to the multimeric supercomplex, stimulating GTP hydrolysis by the bound beta-tubulin and the release of the alpha-/beta-tubulin heterodimer.
Lma05962.t1	TBCC_ARATH	Essential tubulin-folding protein involved in the final step of the tubulin folding pathway. Required for continuous microtubule cytoskeleton organization, mitotic division, cytokinesis, and to couple cell cycle progression to cell division in embryos and endosperms. Not essential for cell viability. Binds probably to the multimeric supercomplex, stimulating GTP hydrolysis by the bound beta-tubulin and the release of the alpha-/beta-tubulin heterodimer.
Lma12386.t1	TBCC_ARATH	Essential tubulin-folding protein involved in the final step of the tubulin folding pathway. Required for continuous microtubule cytoskeleton organization, mitotic division, cytokinesis, and to couple cell cycle progression to cell division in embryos and endosperms. Not essential for cell viability. Binds probably to the multimeric supercomplex, stimulating GTP hydrolysis by the bound beta-tubulin and the release of the alpha-/beta-tubulin heterodimer.
Lma10533.t1	MFDR_ARATH	Associates in vitro with the adrenodoxin-like protein MFDX1 to form an efficient low potential electron transfer chain that is able to reduce cytochrome C (,). Functions as accessory mitochondrial protein involved with BIO2 in the plant biotin synthase reaction

Lma22205.t1	MFDR_ARATH	Associates in vitro with the adrenodoxin-like protein MFDX1 to form an efficient low potential electron transfer chain that is able to reduce cytochrome C (,). Functions as accessory mitochondrial protein involved with BIO2 in the plant biotin synthase reaction
Lma09176.t1	RPS6C_ARATH	Disease resistance (R) protein that specifically recognizes the hopA1 type III effector avirulence protein from Pseudomonas syringae. Resistance proteins guard the plant against pathogens that contain an appropriate avirulence protein via an indirect interaction with this avirulence protein. That triggers a defense system including the hypersensitive response, which restricts the pathogen growth.
Lma09177.t1	RPS6C_ARATH	Disease resistance (R) protein that specifically recognizes the hopA1 type III effector avirulence protein from Pseudomonas syringae. Resistance proteins guard the plant against pathogens that contain an appropriate avirulence protein via an indirect interaction with this avirulence protein. That triggers a defense system including the hypersensitive response, which restricts the pathogen growth.
Lma10525.t1	PUB42_ARATH	Functions as an E3 ubiquitin ligase.
Lma17107.t1	PUB42_ARATH	Functions as an E3 ubiquitin ligase.
Lma09868.t1	UND_ARATH	Probable aspartic protease activated by the transcription factor MYB80. May participate in the regulation of the timing of tapetal programmed cell death (PCD) which is critical for pollen development.
Lma09167.t1	PTC52_ARATH	Part of a translocon most abundantly expressed in etiolated plants and involved in the protochlorophyllide-dependent import of the precursor NADPH:protochlorophyllide oxidoreductase A (pPORA).
Lma13266.t1	PTC52_ARATH	Part of a translocon most abundantly expressed in etiolated plants and involved in the protochlorophyllide-dependent import of the precursor NADPH:protochlorophyllide oxidoreductase A (pPORA).
Lma09925.t1	ALMT3_ARATH	Malate transporter.
Lma18995.t1	ALMT3_ARATH	Malate transporter.
Lma10064.t1	ARR20_ARATH	Putative transcriptional activator that binds specifically to the DNA sequence 5'-[AG]GATT-3'. Functions as response regulator involved in His-to-Asp phosphorelay signal transduction system. Phosphorylation of the Asp residue in the receiver domain activates the ability of the protein to promote the transcription of target genes. Could directly activate some type-A response regulators in response to cytokinins (By similarity).

Lma20591.t1	ARR20_ARATH	Putative transcriptional activator that binds specifically to the DNA sequence 5'-[AG]GATT-3'. Functions as response regulator involved in His-to-Asp phosphorelay signal transduction system. Phosphorylation of the Asp residue in the receiver domain activates the ability of the protein to promote the transcription of target genes. Could directly activate some type-A response regulators in response to cytokinins (By similarity).
Lma10565.t1	OEP61_ARATH	Plays a role in protein import into the endoplasmic reticulum (ER). May function as chaperone docking protein during post-translational protein translocation into the ER. Chaperone receptor mediating Hsp70-dependent protein targeting to chloroplasts. Interacts specifically with some chloroplast precursors, but not with mitochondrial precursors. Able to select precursors for delivery to the chloroplast translocase independently of Hsp70.
Lma12962.t1	OEP61_ARATH	Plays a role in protein import into the endoplasmic reticulum (ER). May function as chaperone docking protein during post-translational protein translocation into the ER. Chaperone receptor mediating Hsp70-dependent protein targeting to chloroplasts. Interacts specifically with some chloroplast precursors, but not with mitochondrial precursors. Able to select precursors for delivery to the chloroplast translocase independently of Hsp70.
Lma10369.t1	SY121_ARATH	Vesicle trafficking protein that functions in the secretory pathway.
Lma10170.t1	ATG1A_ARATH	Serine/threonine protein kinase involved in autophagy in a nutritional condition-dependent manner. The ATG1-ATG13 protein kinase complex regulates downstream events required for autophagosome enclosure and/or vacuolar delivery. Becomes a target of autophagy under nutrient starvation. Connects autophagy to plant nutritional status.
Lma19850.t1	ATG1A_ARATH	Serine/threonine protein kinase involved in autophagy in a nutritional condition-dependent manner. The ATG1-ATG13 protein kinase complex regulates downstream events required for autophagosome enclosure and/or vacuolar delivery. Becomes a target of autophagy under nutrient starvation. Connects autophagy to plant nutritional status.
Lma11003.t1	CNG20_ARATH	Probable cyclic nucleotide-gated ion channel.
Lma19273.t1	CNG20_ARATH	Probable cyclic nucleotide-gated ion channel.
Lma14852.t1	PRS8A_ARATH	The 26S proteasome is involved in the ATP-dependent degradation of ubiquitinated proteins. The regulatory (or ATPase) complex confers ATP dependency and substrate specificity to the 26S complex.

Lma19056.t1	PRS8A_ARATH	The 26S proteasome is involved in the ATP-dependent degradation of ubiquitinated proteins. The regulatory (or ATPase) complex confers ATP dependency and substrate specificity to the 26S complex.
Lma00599.t1	AGL30_ARATH	Probable transcription factor that forms heterodimers with the MADS-box proteins AGL66 and AGL104 and is involved in the regulation of pollen maturation at the late stages of pollen development and pollen tube growth.
Lma19620.t1	CH60B_ARATH	Implicated in mitochondrial protein import and macromolecular assembly. May facilitate the correct folding of imported proteins. May also prevent misfolding and promote the refolding and proper assembly of unfolded polypeptides generated under stress conditions in the mitochondrial matrix (By similarity).
Lma14310.t1	ABI4_ARATH	Transcription regulator that probably binds to the GCC- box pathogenesis-related promoter element. Binds also to the S-box (5'-CACTTCCA-3') photosynthesis-associated nuclear genes-related (PhANGs-related) promoter element, and thus acts as a transcription inhibitor. Involved in the regulation of gene expression by stress factors and by components of stress signal transduction pathways. May have a function in the deetiolation process. Confers sensitivity to abscisic acid (ABA), and regulates the ABA signaling pathway during seed germination, upon nitrate- mediated lateral root inhibition, in hexokinase-dependent sugar responses (including feed-back regulation of photosynthesis and mobilization of storage lipid during germination), and in response to osmotic stress mediated by NaCl, KCl or mannitol. Plays a role in sucrose sensing or signaling, especially at low fluence far red light. Also involved in plant response to glucose treatment, especially at low concentration and in young seedlings. Required for the trehalose-mediated root inhibition and starch accumulation in cotyledons, probably by inhibiting starch breakdown. However, seems to not be involved in sugar-mediated senescence. Required for the ABA-dependent beta-amino-butyric acid (BABA) signaling pathway. BABA primes ABA synthesis and promotes resistance to drought and salt, and leads to a prime callose accumulation that confers resistance against necrotrophic pathogens such as A.brassicicola and P.cucumerina. Seems to be involved in resistance to S.sclerotiorum probably by regulating the ABA- mediated stomatal closure apparently by antagonistic interaction with oxalate. Negative regulator of low water potential-induced Pro accumulation whose effect is decreased by high levels of sugar.

Lma25376.t1	ABI4_ARATH	Transcription regulator that probably binds to the GCC- box pathogenesis-related promoter element. Binds also to the S-box (5'-CACTTCCA-3') photosynthesis-associated nuclear genes-related (PhANGs-related) promoter element, and thus acts as a transcription inhibitor. Involved in the regulation of gene expression by stress factors and by components of stress signal transduction pathways. May have a function in the deetiolation process. Confers sensitivity to abscisic acid (ABA), and regulates the ABA signaling pathway during seed germination, upon nitrate- mediated lateral root inhibition, in hexokinase-dependent sugar responses (including feed-back regulation of photosynthesis and mobilization of storage lipid during germination), and in response to osmotic stress mediated by NaCl, KCl or mannitol. Plays a role in sucrose sensing or signaling, especially at low fluence far red light. Also involved in plant response to glucose treatment, especially at low concentration and in young seedlings. Required for the trehalose-mediated root inhibition and starch accumulation in cotyledons, probably by inhibiting starch breakdown. However, seems to not be involved in sugar-mediated senescence. Required for the ABA-dependent beta-amino-butyric acid (BABA) signaling pathway. BABA primes ABA synthesis and promotes resistance to drought and salt, and leads to a prime callose accumulation that confers resistance against necrotrophic pathogens such as A.brassicicola and P.cucumerina. Seems to be involved in resistance to S.sclerotiorum probably by regulating the ABA- mediated stomatal closure apparently by antagonistic interaction with oxalate. Negative regulator of low water potential-induced Pro accumulation whose effect is decreased by high levels of sugar.
Lma14968.t1	TSL_ARATH	Required for correct initiation of floral organ primordia and for proper development of organ primordia. Phosphorylates in vitro ASF1B/SGA1, the C-terminal part of TKI1 and histone H3.
Lma12881.t1	GSO1_ARATH	Together with GSO2, receptor-like serine/threonine- kinase required during the development of the epidermal surface in embryos and cotyledonsIn coordination with GSO2, regulates root growth through control of cell division and cell fate specification. Controls seedling root growth by modulating sucrose response after germinationReceptor of the peptide hormones CIF1 and CIF2 required for contiguous Casparian strip diffusion barrier formation in rootsRequired for localizing CASP proteins into the Casparian strip following an uninterrupted, ring-like domain, to trigger endodermal differentiation and thus regulate potassium ion (K) homeostasis. Involved in the maintenance of water transport and root pressure. May also be involved in the regulation of suberin accumulation in the endodermis

Lma12882.t1	DR100_ARATH	This protein is able to complement bacterial recA mutations, but its native function in the plant is not known.
Lma14373.t1	ERD15_ARATH	Central component of stress responses that interacts with poly(A)-binding proteins. Negative regulator of abscisic acid (ABA) responses, including resistance to drought and freezing as well as stomatal closure regulation. Mediates resistance to the bacterial necrotroph pathogen Erwinia carotovora subsp. carotovora and promotes the induction of marker genes for systemic acquired resistance (SAR).
Lma26266.t1	ERD15_ARATH	Central component of stress responses that interacts with poly(A)-binding proteins. Negative regulator of abscisic acid (ABA) responses, including resistance to drought and freezing as well as stomatal closure regulation. Mediates resistance to the bacterial necrotroph pathogen Erwinia carotovora subsp. carotovora and promotes the induction of marker genes for systemic acquired resistance (SAR).
Lma13658.t1	ASNA1_CHLRE	ATPase required for the post-translational delivery of tail-anchored (TA) proteins to the chloroplast. Required for the accumulation of TOC34, an essential component of the outer chloroplast membrane translocon (TOC) complex (,). Recognizes and selectively binds the transmembrane domain of TA proteins in the cytosol. This complex then targets to chloroplast, where the tail-anchored protein is released for insertion. This process is regulated by ATP binding and hydrolysis
Lma21263.t1	XTH24_ARATH	Catalyzes xyloglucan endohydrolysis (XEH) and/or endotransglycosylation (XET). Cleaves and religates xyloglucan polymers, an essential constituent of the primary cell wall, and thereby participates in cell wall construction of growing tissues. May be required during development to modify the walls of cells under mechanical stress.
Lma25567.t1	XTH24_ARATH	Catalyzes xyloglucan endohydrolysis (XEH) and/or endotransglycosylation (XET). Cleaves and religates xyloglucan polymers, an essential constituent of the primary cell wall, and thereby participates in cell wall construction of growing tissues. May be required during development to modify the walls of cells under mechanical stress.
Lma20766.t1	GAUT3_ARATH	May be involved in pectin and/or xylans biosynthesis in cell walls.
Lma20768.t1	GAUT3_ARATH	May be involved in pectin and/or xylans biosynthesis in cell walls.
Lma21540.t1	CDPKM_ARATH	May play a role in signal transduction pathways that involve calcium as a second messenger.
Lma21541.t1	CDPKM_ARATH	May play a role in signal transduction pathways that involve calcium as a second messenger.
Lma10785.t1	MSL6_ARATH	Mechanosensitive channel that opens in response to stretch forces in the membrane lipid bilayer.
Lma22972.t1	MSL6_ARATH	Mechanosensitive channel that opens in response to stretch forces in the membrane lipid bilayer.

Lma05615.t1	SNL4_ARATH	Acts as a transcriptional repressor. Plays roles in regulating gene expression and genome stability (By similarity).
Lma22448.t1	SNL4_ARATH	Acts as a transcriptional repressor. Plays roles in regulating gene expression and genome stability (By similarity).
Lma22179.t1	ATX4_ARATH	Histone methyltransferase.
Lma24547.t1	ATX5_ARATH	Histone methyltransferase.
Lma10784.t1	PRB1_ARATH	Probably involved in the defense reaction of plants against pathogens.
Lma21102.t1	PRB1_ARATH	Probably involved in the defense reaction of plants against pathogens.
Lma00244.t1	4CL2_ARATH	Produces CoA thioesters of a variety of hydroxy- and methoxy-substituted cinnamic acids, which are used to synthesize several phenylpropanoid-derived compounds, including anthocyanins, flavonoids, isoflavonoids, coumarins, lignin, suberin and wall- bound phenolics.
Lma15368.t1	4CL4_ARATH	Produces CoA thioesters of a variety of hydroxy- and methoxy-substituted cinnamic acids, which are used to synthesize several phenylpropanoid-derived compounds, including anthocyanins, flavonoids, isoflavonoids, coumarins, lignin, suberin and wall- bound phenolics.
Lma00102.t1	PME2_ARATH	Acts in the modification of cell walls via demethylesterification of cell wall pectin.
Lma12411.t1	CESA4_ARATH	Catalytic subunit of cellulose synthase terminal complexes ('rosettes'), required for beta-1,4-glucan microfibril crystallization, a major mechanism of the cell wall formation. Involved in the secondary cell wall formation. Required for the xylem cell wall thickening.
Lma15231.t1	CESA4_ARATH	Catalytic subunit of cellulose synthase terminal complexes ('rosettes'), required for beta-1,4-glucan microfibril crystallization, a major mechanism of the cell wall formation. Involved in the secondary cell wall formation. Required for the xylem cell wall thickening.
Lma15105.t1	PR19B_ARATH	Probable ubiquitin-protein ligase which is mainly involved pre-mRNA splicing and DNA repair (By similarity). Component of the MAC complex that probably regulates defense responses through transcriptional control and thereby is essential for plant innate immunity.
Lma07599.t1	HR2_ARATH	Probable disease resistance (R) protein.
Lma07600.t1	HR2_ARATH	Probable disease resistance (R) protein.
Lma17690.t1	VP241_ARATH	Component of the ESCRT-III complex, which is required for multivesicular bodies (MVBs) formation and sorting of endosomal cargo proteins into MVBs. The ESCRT-III complex is probably involved in the concentration of MVB cargo (By similarity).

Lma07921.t1	ARR9_ARATH	Functions as response regulator involved in His-to-Asp phosphorelay signal transduction system. Phosphorylation of the Asp residue in the receiver domain activates the ability of the protein to promote the transcription of target genes. Type-A response regulators seem to act as negative regulators of the cytokinin signaling.
Lma24339.t1	ARR9_ARATH	Functions as response regulator involved in His-to-Asp phosphorelay signal transduction system. Phosphorylation of the Asp residue in the receiver domain activates the ability of the protein to promote the transcription of target genes. Type-A response regulators seem to act as negative regulators of the cytokinin signaling.
Lma06261.t1	RFS4_ARATH	Transglycosidase operating by a ping-pong reaction mechanism. Involved in the synthesis of raffinose, a major soluble carbohydrate in seeds, roots and tubers (By similarity).
Lma10737.t1	RFS4_ARATH	Transglycosidase operating by a ping-pong reaction mechanism. Involved in the synthesis of raffinose, a major soluble carbohydrate in seeds, roots and tubers (By similarity).
Lma17915.t1	RK3B_ARATH	One of the primary rRNA binding proteins, it binds directly near the 3'-end of the 23S rRNA, where it nucleates assembly of the 50S subunit.
Lma25041.t1	RK3B_ARATH	One of the primary rRNA binding proteins, it binds directly near the 3'-end of the 23S rRNA, where it nucleates assembly of the 50S subunit.
Lma12130.t2	RFI2_ARATH	Mediates phytochrome (phyA and phyB)-controlled seedling deetiolation responses such as hypocotyl elongation in response to red and far-red light (,). Required for light-induced expression of LHCB3 and CHALCONE SYNTHASE (CHS)Regulates negatively CONSTANS (CO) and FLOWERING LOCUS T (FT) expression and photoperiodic flowering
Lma11554.t1	CHR7_ARATH	Chromatin remodeling factor that represses the expression of embryonic trait genes upon and after seed germination and thus enables the developmental switch to post- germinative growth.
Lma11671.t1	ALFL1_ARATH	Histone-binding component that specifically recognizes H3 tails trimethylated on 'Lys-4' (H3K4me3), which mark transcription start sites of virtually all active genes.
Lma11672.t1	ALFL1_ORYSJ	Histone-binding component that specifically recognizes H3 tails trimethylated on 'Lys-4' (H3K4me3), which mark transcription start sites of virtually all active genes.
Lma10862.t1	SOT7_ARATH	Sulfotransferase that utilizes 3'-phospho-5'-adenylyl sulfate (PAPS) as sulfonate donor.
Lma10863.t1	SOT7_ARATH	Sulfotransferase that utilizes 3'-phospho-5'-adenylyl sulfate (PAPS) as sulfonate donor.
Lma01051.t1	ZAT7_ARATH	Probable transcription factor involved in oxidative stress response.
Lma01052.t1	ZAT9_ARATH	Probable transcription factor that may be involved in stress responses.

Lma00566.t1	MTM1_ARATH	Involved in the mitochondrial activation of MSD1 by specifically facilitating insertion of the essential manganese cofactor. Has the ability to activate iron regulon in an iron- dependent manner.
Lma24291.t1	MTM1_ARATH	Involved in the mitochondrial activation of MSD1 by specifically facilitating insertion of the essential manganese cofactor. Has the ability to activate iron regulon in an iron- dependent manner.
Lma24858.t1	TIL_ARATH	Involved in basal (BT) and acquired thermotolerance (AT), probably by preventing plasma membrane lipids peroxidation induced by severe heat-shock (HS) (,). Lipocalin that confers protection against oxidative stress caused by heat, freezing, paraquat and light (,). Confers resistance to high salt (NaCl) levels, probably by protecting chloroplasts from ion toxicity via ion homeostasis maintenance (,). Required for seed longevity by insuring polyunsaturated lipids integrity
Lma24859.t1	TIL_ARATH	Involved in basal (BT) and acquired thermotolerance (AT), probably by preventing plasma membrane lipids peroxidation induced by severe heat-shock (HS) (,). Lipocalin that confers protection against oxidative stress caused by heat, freezing, paraquat and light (,). Confers resistance to high salt (NaCl) levels, probably by protecting chloroplasts from ion toxicity via ion homeostasis maintenance (,). Required for seed longevity by insuring polyunsaturated lipids integrity
Lma04613.t1	CDPKF_ARATH	May play a role in signal transduction pathways that involve calcium as a second messenger.
Lma04614.t1	CDPKF_ARATH	May play a role in signal transduction pathways that involve calcium as a second messenger.
Lma04080.t1	VQ11_ARATH	May modulate WRKY transcription factor activities.
Lma05255.t1	VQ11_ARATH	May modulate WRKY transcription factor activities.
Lma25903.t1	RDR5_ARATH	Probably involved in the RNA silencing pathway and required for the generation of small interfering RNAs (siRNAs).
Lma26713.t1	PILR1_ARATH	Reductase involved in lignan biosynthesis. Unlike conventional pinoresinol reductases that can reduce both pinoresinol and lariciresinol, PRR1 shows a strict substrate preference toward pinoresinol. Active on both (+) and (-)- pinoresinol. Abstracts the 4R-hydride from the NADPH cofactor during catalysis.
Lma08747.t1	SPL5_ARATH	Trans-acting factor that binds specifically to the consensus nucleotide sequence 5'-TNCGTACAA-3' of AP1 promoter. Promotes both vegetative phase change and flowering.
Lma10561.t1	PSK3_ARATH	Promotes plant cell differentiation, organogenesis and somatic embryogenesis as well as cell proliferation.
Lma10265.t1	MORF9_ARATH	Involved in organellar RNA editing. Required for the processing of multiple editing sites in plastids.

Lma09902.t2	PAE5_ARATH	Hydrolyzes acetyl esters in homogalacturonan regions of pectin. In type I primary cell wall, galacturonic acid residues of pectin can be acetylated at the O-2 and O-3 positions. Decreasing the degree of acetylation of pectin gels in vitro alters their physical properties.
Lma09353.t7	ERD2B_ARATH	Determines the specificity of the luminal endoplasmic reticulum protein retention system. Required for the retro- transport of calreticulin-3 (CRT3) from the Golgi to the ER. Specifically required for elongation factor Tu receptor (EFR) function in response to the pathogen-associated molecular pattern (PAMP) elf18.
Lma10586.t1	LEUC_ARATH	Catalyzes the isomerization between 2-isopropylmalate and 3-isopropylmalate, via the formation of 2-isopropylmaleate.
Lma10383.t1	ACA1_ARATH	This magnesium-dependent enzyme catalyzes the hydrolysis of ATP coupled with the translocation of calcium from the cytosol out of the cell or into organelles.
Lma10120.t1	RNS1_ARATH	May remobilize phosphate, particularly when cells senesce or when phosphate becomes limiting.
Lma09467.t2	IAA14_ARATH	Aux/IAA proteins are short-lived transcriptional factors that function as repressors of early auxin response genes at low auxin concentrations. Repression is thought to result from the interaction with auxin response factors (ARFs), proteins that bind to the auxin-responsive promoter element (AuxRE). Formation of heterodimers with ARF proteins may alter their ability to modulate early auxin response genes expression.
Lma08678.t1	VRN2_ARATH	Polycomb group (PcG) protein. Plays a central role in vernalization by maintaining repressed the homeotic gene FLC, a floral repressor, after a cold treatment. PcG proteins act by forming multiprotein complexes, which are required to maintain the transcriptionally repressive state of homeotic genes throughout development. PcG proteins are not required to initiate repression, but to maintain it during later stages of development. They probably act via the methylation of histones, rendering chromatin heritably changed in its expressibility. Associates constitutively along the whole FLC locus.
Lma08633.t1	PER41_ARATH	Removal of H(2)O(2), oxidation of toxic reductants, biosynthesis and degradation of lignin, suberization, auxin catabolism, response to environmental stresses such as wounding, pathogen attack and oxidative stress. These functions might be dependent on each isozyme/isoform in each plant tissue.
Lma09045.t1	LUP2_ARATH	Multifunctional enzyme that converts oxidosqualene to nine different triterpenes, mainly lupeol, beta-amyrin and alpha- amyrin in a 15:50:30 ratio.

Lma08990.t1	EIF3I_ARATH	Component of the eukaryotic translation initiation factor 3 (eIF-3) complex, which is involved in protein synthesis of a specialized repertoire of mRNAs and, together with other initiation factors, stimulates binding of mRNA and methionyl-tRNAi to the 40S ribosome. The eIF-3 complex specifically targets and initiates translation of a subset of mRNAs involved in cell proliferation.
Lma10668.t1	PHB4_ARATH	Prohibitin probably acts as a holdase/unfoldase for the stabilization of newly synthesized mitochondrial proteins.
Lma09716.t1	NANA_ARATH	Aspartic proteinase that can use azocasein as substrate and regulates endogenous sugar levels (e.g. sucrose, glucose and fructose) by modulating starch accumulation and remobilizationInvolved in the maintenance of the shoot apical meristem (SAM)Influences general morphology and development
Lma09306.t1	NIPL1_ARATH	May be involved in the early steps of the plant defense signaling pathway.
Lma10063.t1	QCR6_SOLTU	This is a component of the ubiquinol-cytochrome c reductase complex (complex III or cytochrome b-c1 complex), which is part of the mitochondrial respiratory chain. This protein may mediate formation of the complex between cytochromes c and c1.
Lma09889.t1	TI141_ARATH	Component of the PAM complex, a complex required for the translocation of transit peptide-containing proteins from the inner membrane into the mitochondrial matrix in an ATP-dependent manner.
Lma08393.t1	ERLL2_ARATH	Probable lipid transfer protein (LTP). May improve freezing survival. Seems to control the flowering process and lignin synthesis. Confers resistance to Botrytis cinerea.
Lma25636.t1	REV3_ARATH	Catalytic subunit of the error prone DNA polymerase zeta. Involved in damage-tolerance mechanisms through translesion DNA synthesis.
Lma25339.t1	SOT17_ARATH	Sulfotransferase that utilizes 3'-phospho-5'-adenylyl sulfate (PAPS) as sulfonate donor to catalyze the sulfate conjugation of desulfo-glucosinolates (dsGSs), the final step in the biosynthesis of the glucosinolate core structure. Substrate preference is desulfo-benzyl glucosinolate > desulfo-6-methylthiohexyl glucosinolate. Increased specific activity with increasing chain length of desulfo-glucosinolate derived from methionine. Preferred substrate is desulfo-8-methylthiooctyl glucosinolate.
Lma25495.t1	KNAT1_ARATH	May play a role in meristem function, and may be involved in maintaining cells in an undifferentiated, meristematic state, and its expression disappears at the same time the shoot apex undergoes the transition from vegetative to reproductive developmentPositive regulator of LATERAL ORGAN BOUNDARIES (LOB)Probably binds to the DNA sequence 5'-TGAC-3'Able to traffic from the L1 to the L2/L3 layers of the meristem, presumably through plasmodesmata

Lma25513.t1	XPB1_ARATH	ATP-dependent 3'-5' DNA helicase, component of the general transcription and DNA repair factor IIH (TFIIH) core complex, which is involved in general and transcription-coupled nucleotide excision repair (NER) of damaged DNA and, when complexed to CAK, in RNA transcription by RNA polymerase II. In NER, TFIIH acts by opening DNA around the lesion to allow the excision of the damaged oligonucleotide and its replacement by a new DNA fragment. The ATPase activity of XPB, but not its helicase activity, is required for DNA opening. In transcription, TFIIH has an essential role in transcription initiation. When the pre- initiation complex (PIC) has been established, TFIIH is required for promoter opening and promoter escape. The ATP-dependent helicase activity of XPB is required for promoter opening and promoter escape. Phosphorylation of the C-terminal tail (CTD) of the largest subunit of RNA polymerase II by the kinase module CAK controls the initiation of transcription (By similarity). Required during the early stages of development, including seed germination
Lma26451.t1	RL51_ARATH	Component of the ribosome, a large ribonucleoprotein complex responsible for the synthesis of proteins in the cell. The small ribosomal subunit (SSU) binds messenger RNAs (mRNAs) and translates the encoded message by selecting cognate aminoacyl- transfer RNA (tRNA) molecules. The large subunit (LSU) contains the ribosomal catalytic site termed the peptidyl transferase center (PTC), which catalyzes the formation of peptide bonds, thereby polymerizing the amino acids delivered by tRNAs into a polypeptide chain. The nascent polypeptides leave the ribosome through a tunnel in the LSU and interact with protein factors that function in enzymatic processing, targeting, and the membrane insertion of nascent chains at the exit of the ribosomal tunnel.
Lma25392.t1	ARR1_ARATH	Transcriptional activator that binds specifically to the DNA sequence 5'-[AG]GATT-3'. Functions as a response regulator involved in His-to-Asp phosphorelay signal transduction system. Phosphorylation of the Asp residue in the receiver domain activates the ability of the protein to promote the transcription of target genes. Could directly activate some type-A response regulators in response to cytokinins. Regulates SHY2 by binding to its promoterInvolved in the root-meristem size determination through the regulation of cell differentiation
Lma25390.t1	GAT17_ARATH	Transcriptional regulator that specifically binds 5'- GATA-3' or 5'-GAT-3' motifs within gene promoters.
Lma19659.t1	PHB4_ARATH	Prohibitin probably acts as a holdase/unfoldase for the stabilization of newly synthesized mitochondrial proteins.

Lma20753.t1	EIF3A_ARATH	RNA-binding component of the eukaryotic translation initiation factor 3 (eIF-3) complex, which is involved in protein synthesis of a specialized repertoire of mRNAs and, together with other initiation factors, stimulates binding of mRNA and methionyl-tRNAi to the 40S ribosome. The eIF-3 complex specifically targets and initiates translation of a subset of mRNAs involved in cell proliferation.
Lma18883.t1	AGP4_ARATH	Proteoglycan that seems to be implicated in diverse developmental roles such as differentiation, cell-cell recognition, embryogenesis and programmed cell death.
Lma18872.t1	ASPA_ARATH	Probably not redundant with AED1 and not involved in restriction of salicylic acid (SA) or systemic acquired resistance (SAR) signaling.
Lma19194.t1	AHL5_ARATH	Transcription factor that specifically binds AT-rich DNA sequences related to the nuclear matrix attachment regions (MARs).
Lma20179.t2	PBL1_ARATH	Contributes to pathogen-associated molecular pattern (PAMP)-triggered immunity (PTI) signaling and defense responses downstream of FLS2. Acts additively with BIK1 in PTI defensesSeems not required for flg22-induced MAPK activation (Probable). Required for Pep1-induced defenses. Pep1 is an endogenous elicitor that potentiates PAMP-inducible plant responses
Lma20481.t1	ZAT9_ARATH	Probable transcription factor that may be involved in stress responses.
Lma20444.t1	ASK4_ARATH	Involved in ubiquitination and subsequent proteasomal degradation of target proteins. Together with CUL1, RBX1 and a F- box protein, it forms a SCF E3 ubiquitin ligase complex. The functional specificity of this complex depends on the type of F- box protein. In the SCF complex, it serves as an adapter that links the F-box protein to CUL1 (By similarity).
Lma20340.t1	MYO16_ARATH	Myosin heavy chain that is required for the cell cycle- regulated transport of various organelles and proteins for their segregation. Functions by binding with its tail domain to receptor proteins on organelles and exerting force with its N-terminal motor domain against actin filaments, thereby transporting its cargo along polarized actin cables (By similarity).
Lma20478.t1	SC61B_ARATH	Necessary for protein translocation in the endoplasmic reticulum.
Lma19288.t3	LOG6_ARATH	Cytokinin-activating enzyme working in the direct activation pathway. Phosphoribohydrolase that converts inactive cytokinin nucleotides to the biologically active free-base forms (By similarity).
Lma20287.t1	WIH2_ARATH	Required for the promotion of megasporogenesis, or promotion of germ cell formation from somatic precursor cells. Acts redundantly with WIH1. Functions in a genetic pathway downstream of SPL/NZZ and WUS and together with TRN2 in promoting megasporogenesis.
Lma19101.t1	MYOB7_ARATH	Membrane-anchored myosin receptors that define a distinct, plant-specific transport vesicle compartment.

Lma19797.t1	HUMS_ARATH	Involved in sesquiterpene (C15) biosynthesis. The major products are beta-caryophyllene and alpha-humulene. Does not convert geranyl diphosphate (GPP) to any monoterpenes.
Lma19892.t1	FIP37_ARATH	Probable regulatory subunit of the N6-methyltransferase complex, a multiprotein complex that mediates N6-methyladenosine (m6A) methylation at the 5'-[AG]GAC-3' consensus sites of some mRNAs (,). Associates with MTA, MTB, VIR and HAKAI to form the m6A writer complex which is essential for adenosine methylation at specific mRNA sequencesN6-methyladenosine (m6A) plays a role in mRNA stability, processing, translation efficiency and editing (,). Essential protein required during endosperm development and embryogenesis. Involved in endoreduplication, especially in trichomes. May play a role in splicing events
Lma18681.t1	NACA1_ARATH	May promote appropriate targeting of ribosome-nascent polypeptide complexes.
Lma20712.t1	RBG7_ARATH	Plays a role in RNA transcription or processing during stress. Binds RNAs and DNAs sequence with a preference to single- stranded nucleic acids. Displays strong affinity to poly(U) and poly(G) sequence. Involved in mRNA alternative splicing of numerous targets by modulating splice site selection. Negatively regulates the circadian oscillations of its own transcript as well as RBG8 transcript. Forms an interlocked post-transcriptional negative feedback loop with the RBG8 autoregulatory circuit. Both proteins negatively autoregulate and reciprocally crossregulate by binding to their pre-mRNAs and promoting unproductive splicing coupled to degradation via the NMD pathway. Involved in the regulation of abscisic acid and stress responses. Affects the growth and stress tolerance under high salt and dehydration stress conditions, and also confers freezing tolerance, particularly via the regulation of stomatal opening and closing in the guard cells. Exhibits RNA chaperone activity during the cold adaptation process. Involved in the export of mRNAs from the nucleus to the cytoplasm under cold stress conditions. Target of the Pseudomonas syringae type III effector HopU1, which could probably be involved in plant innate immunity. Component of the flowering autonomous pathway which promotes floral transition, at least partly by down- regulating FLC.
Lma20309.t1	RMR41_ARATH	Collaborates with REM4.2 to positively regulate the BCTV and BSCTV susceptibility.
Lma18665.t1	LAZY1_ARATH	Involved in the regulation of shoot gravitropism (,). Involved in the regulation of inflorescence branch angle

Lma20595.t1	XXT1_ARATH	Xylosyltransferase specific to UDP-D-xylose that accepts both cellopentaose and cellohexaose as substrates, with a better use of cellohexaose, to produce xyloglucan. Adds preferentially the first xylosyl residue to the fourth glucosyl residue from the reducing end of both acceptors. Transfer one xylose mainly to the second glucose residue from the non-reducing end. The acceptor should have a minimum of four glucose residues.
Lma19641.t1	TGT1_ARATH	Probable transcription factor that binds specifically to the core DNA sequence 5'-GGTTAA-3'. May act as a molecular switch in response to light signals.
Lma19780.t1	CLPC2_ARATH	Molecular chaperone (, ,). May act as a suppressor of FtsH-mediated thylakoid membrane biogenesis and may enhance photoinhibitionSeems not involved in chloroplastic protein importProbable component of the TIC-associated stromal import motor involved in inner membrane translocationHas an ATPase activity, but no ADPase activityInteracts with transit peptides with a positional preference (,). Localization of the signal sequence at the N-terminal end of a protein seems mandatory for interaction to take place
Lma20637.t1	LFG2_ARATH	Regulates the brassinosteroid (BR) signaling pathway that mediates cell elongation and organ morphogenesis
Lma20637.t1	LFG2_ARATH	(Microbial infection) May prevent cell death upon A.alternata f.sp. lycopersici (AAL) toxin treatment.
Lma20315.t1	AGP23_ARATH	Proteoglycan that seems to be implicated in diverse developmental roles such as differentiation, cell-cell recognition, embryogenesis and programmed cell death.
Lma19275.t1	HIP6_ARATH	Heavy-metal-binding protein. Involved in the maintenance of heavy metal homeostasis and/or in detoxification.
Lma18790.t1	BIG1D_ARATH	Involved in auxin transport. Regulator of the auxin signaling pathway.
Lma18745.t1	CLE40_ARATH	Extracellular signal peptide secreted by differentiated root cells that regulates root cell fate. Acts with ACR4 as a ligand-receptor pair in a signal transduction pathway, coordinating movement of the root tip and organization of cell divisions in the root meristem. Promotes cell differentiation in the distal root meristem in a dose-dependent manner, especially the transition from columella stem cells (CSC) daughters into columella cells (CCs). Induces ACR4 expression in root quiescent center (QC). Involved in WUX5 QC-specific expression pattern regulation.
Lma25797.t1	ZW10_ARATH	May be required for accurate chromosome segregation. Required for proper maturation of seed storage proteins. Forms a complex with MAG2, MIP2 and MIP3 on the endoplasmic reticulum that may be responsible for efficient transport of seed storage proteins.

Lma13269.t1	PME1_ARATH	Acts in the modification of cell walls via demethylesterification of cell wall pectin (By similarity). Demethylates protein phosphatase 2A (PP2A) that have been reversibly carboxymethylated by LCMT1. Acts as negative regulators of genes involved in salt stress response
Lma14343.t2	SCAB2_ARATH	Probable plant-specific actin binding protein that bundles and stabilizes microfilaments (MFs).
Lma14396.t1	ZED1_ARATH	Probable non-functional pseudokinase required for recognition of the Pseudomonas syringae type III effector HopZ1a by ZAR1. May function as a decoy to trap HopZ1a in the ZAR1 complex for recognition by the plant immune system.
Lma14372.t1	WIH2_ARATH	Required for the promotion of megasporogenesis, or promotion of germ cell formation from somatic precursor cells. Acts redundantly with WIH1. Functions in a genetic pathway downstream of SPL/NZZ and WUS and together with TRN2 in promoting megasporogenesis.
Lma14137.t1	PUB2_ARATH	Functions as an E3 ubiquitin ligase.
Lma12479.t1	FRO3_ARATH	Ferric chelate reductase involved in iron reduction in roots. May participate in the transport of electrons to a Fe(3+) ion via FAD and heme intermediates.
Lma14108.t1	CRWN4_ARATH	Component of SUN-protein-containing multivariate complexes also called LINC complexes which link the nucleoskeleton and cytoskeleton by providing versatile outer nuclear membrane attachment sites for cytoskeletal filaments (By similarity). Required for nucleus structure organization (e.g. size and shape) (,). Involved in the maintenance of interphase chromocenter integrity and organization
Lma12810.t1	GRS11_ARATH	May only reduce GSH-thiol disulfides, but not protein disulfides.
Lma14074.t1	PABN2_ARATH	Involved in the 3'-end formation of mRNA precursors (pre-mRNA) by the addition of a poly(A) tail of 200 250 nt to the upstream cleavage product. Stimulates poly(A) polymerase (PAPOLA) conferring processivity on the poly(A) tail elongation reaction and controls also the poly(A) tail length. Increases the affinity of poly(A) polymerase for RNA. Binds to poly(A) and to poly(G) with high affinity. May protect the poly(A) tail from degradation.
Lma13458.t1	RPK1_ARATH	Involved in the main abscisic acid-mediated (ABA) signaling pathway and in early ABA perception. Together with RPK2, required for pattern formation along the radial axis (e.g. the apical embryonic domain cell types that generate cotyledon primordia), and the apical-basal axis (e.g. differentiation of the basal pole during early embryogenesis).
Lma13193.t1	MCES1_ARATH	mRNA-capping methyltransferase that methylates the N7 position of the added guanosine to the 5'-cap structure of mRNAs. Binds RNA containing 5'-terminal GpppC (By similarity).

Lma14148.t1	ERF10_ARATH	Probably acts as a transcriptional activator. Binds to the GCC-box pathogenesis-related promoter element. May be involved in the regulation of gene expression by stress factors and by components of stress signal transduction pathways (By similarity).
Lma13276.t1	P2C56_ARATH	Key component and repressor of the abscisic acid (ABA) signaling pathway that regulates numerous ABA responses, such as stomatal closure, osmotic water permeability of the plasma membrane (Pos), drought-induced resistance and rhizogenesis, response to glucose, high light stress, seed germination and inhibition of vegetative growth. During the stomatal closure regulation, modulates the inward calcium-channel permeability as well as the actin reorganization in guard cells in response to ABA. Involved in the resistance to the bacterial pathogen Pseudomonas syringae pv. tomato. Controls negatively fibrillin expression that is involved in mediating ABA-induced photoprotection. May be involved in ABA content regulation. Plays a role in the Pro accumulation in response to reduced water availability (low water potential). Required for the ABA negative regulation of the ethylene-induced hyponastic growth. Involved in acquired thermotolerance of root growth and seedling survival. Activates/represses SRK2E/OST1 in response to ABA-dependent stimuli, especially in stomata closure regulation involving SLAC1. Represses MAPKKK18 activity and promotes MAPKKK18 degradation by the proteasome pathway upon abscisic acid (ABA) treatmentRepresses KIN10 activity by the specific dephosphorylation of its T-loop Thr-198, leading to a poststress inactivation of SnRK1 signaling
Lma13793.t1	VIP4_ARATH	Component of the PAF1 complex (PAF1C) which is involved in histone modifications such as methylation on histone H3 'Lys-4' (H3K4me3)Involved in regulation of flowering time. Required for the expression of the flowering repressor and MADS box gene FLCInvolved in the control of seed dormancy and germination
Lma13646.t1	DSK2A_ARATH	Binds and presumably selects ubiquitin-conjugates for destruction. Prefers multiubiquitin chains rather than single ubiquitins, with a binding affinity for 'Lys-48'-linked ubiquitin chains. Acts as a ubiquitin receptor that associates with the 26S proteasomal docking subunit RPN10 for the indirect recognition of ubiquitinated substrates of ubiquitin/26S proteasome-mediated proteolysis (UPP).
Lma14376.t1	ATS3A_ARATH	May play a role during embryo development.

Lma13875.t1	MYB96_ARATH	Transcription activator involved in the activation of cuticular wax biosynthesis under drought stress. Binds directly to DNA consensus sequences found in the promoters of genes encoding very-long-chain fatty acid-condensing enzymes involved in cuticular wax biosynthesisFunctions together with MYB94 in the activation of cuticular wax biosynthesisInvolved in drought stress response through abscisic acid (ABA) signaling. Mediates ABA signals that enhance plant resistance to drought by reducing stomatal opening. Mediates ABA-auxin cross-talk to regulate lateral root growth under drought stress conditionsInvolved in the regulation of ABA biosynthesis and ABA-dependent seed dormancy state. Binds to the promoters of NCED2 and NCED6, which are enzymes catalyzing the first step of ABA biosynthesisRegulates seed germination by controlling the expression of ABI4, a repressor of lipid breakdown during seed germinationBinds to the promoter of LTP3 and transactivates LTP3 gene in response to drought stress and freezingInvolved in cold stress response. Binds directly to the promoters of heptahelical protein (HHP) genes in response to cold stress. HHPs modulate the expression of SCRM/ICE1, SCRM2/ICE2 and CAMTA3, which are upstream regulators of cold-responsive C-repeat-binding factors (CBFs)Involved in defense responses against the bacterial pathogen Pseudomonas syringae. May act as a molecular link that mediates cross-talks between ABA and salicylateInvolved in a crosstalk between the circadian clock and ABA signaling. Binds directly to the promoter of APRR1/TOC1 to activate its expression
Lma13214.t1	OEP61_ARATH	Plays a role in protein import into the endoplasmic reticulum (ER). May function as chaperone docking protein during post-translational protein translocation into the ER. Chaperone receptor mediating Hsp70-dependent protein targeting to chloroplasts. Interacts specifically with some chloroplast precursors, but not with mitochondrial precursors. Able to select precursors for delivery to the chloroplast translocase independently of Hsp70.
Lma12580.t1	PHB2_ARATH	Prohibitin probably acts as a holdase/unfoldase for the stabilization of newly synthesized mitochondrial proteins.
Lma14428.t2	PCO4_ARATH	Oxidizes N-terminal cysteine residues, thus preparing the protein for N-end rule pathway-mediated proteasomal degradation.
Lma12928.t1	SCP32_ARATH	Probable carboxypeptidase.
Lma12813.t1	SCRK1_MAIZE	May play an important role in maintaining the flux of carbon towards starch formation in endosperm. May also be involved in a sugar-sensing pathway.

Lma13067.t1	RAN4_ARATH	GTP-binding protein involved in nucleocytoplasmic transport. Required for the import of protein into the nucleus and also for RNA export. Involved in chromatin condensation and control of cell cycle (By similarity).
Lma13332.t1	RA51D_ARATH	Involved in the homologous recombination repair (HRR) pathway of double-stranded DNA breaks arising during DNA replication or induced by DNA-damaging agents.
Lma14053.t1	HIT4_ARATH	Essential protein required for basal thermotolerance, especially during heat-induced chromocentre decondensation, thus regulating transcriptional gene silencing (TGS).
Lma13548.t1	CYP59_ARATH	PPIases accelerate the folding of proteins. It catalyzes the cis-trans isomerization of proline imidic peptide bonds in oligopeptides. Influences somehow regulation of RNA pol II (CTD) phosphorylation. Binds RNA with preferences for GC-rich sequences. Probably involved in activities connecting transcription and pre-mRNA processing. Involved in brassinostroid response.
Lma13325.t1	CD27B_ARATH	Component of the anaphase promoting complex/cyclosome (APC/C), a cell cycle-regulated E3 ubiquitin-protein ligase complex that controls progression through mitosis and the G1 phase of the cell cycle. The APC/C complex controls several key steps in the cell cycle by mediating ubiquitination and subsequent degradation of target proteins such as cyclins. The APC/C complex is required for the female gametophyte development and is involved in several aspect of development by controlling cell division and cell elongation. Involved in the control of endoreduplication. Functionally redundant with CDC27A in the control of gametophyte development.
Lma25648.t1	COV1_ARATH	Involved in the regulation of vascular patterning in the stem, probably by negatively regulating the differentiation of vascular tissue.
Lma22741.t1	TI10B_ARATH	Repressor of jasmonate responses. Jasmonoyl-isoleucine (JA-Ile) specifically promotes COI1-TIFY10B/JAZ2 interaction. Activated by MYC2, MYC3 and MYC4 transcription factors.
Lma22377.t1	AHL8_ARATH	Transcription factor that specifically binds AT-rich DNA sequences related to the nuclear matrix attachment regions (MARs).
Lma22154.t1	DNJ10_ARATH	Have a continuous role in plant development probably in the structural organization of compartments.
Lma20981.t1	CDPKI_ARATH	May play a role in signal transduction pathways that involve calcium as a second messenger.
Lma21267.t1	VIL2_ARATH	Maybe involved in both the vernalization and photoperiod pathways by regulating gene expression. Binds preferentially to dimethylated histone H3 'Lys-9' (H3K9me2). Promotes flowering in non-inductive photoperiods (e.g. short days) through the maintenance of the epigenetically repressed state of MAF5 via H3K9me2 and plant homeodomain / polycomb repressive complex 2 (PHD-PRC2)-dependent H3K27me3.

Lma20879.t1	GEM_ARATH	Involved in the spatial control of cell division, patterning and differentiation of Arabidopsis root epidermal cells. Could be part of a complex that negatively modulates GLABRA2 and CAPRICE expression via the maintenance of a repressor histone H3 epigenetics status of the GL2 and CPC promoters.
Lma20800.t1	TIP23_ARATH	Transports methylammonium or ammonium in yeast cells, preferentially at high medium pH. May participate in vacuolar compartmentation and detoxification of ammonium.
Lma21049.t1	CIF2_ARATH	Peptide hormone required for contiguous Casparian strip diffusion barrier formation in roots via the regulation of CASPs protein expression and distribution in a GSO1-GSO2 signaling pathway. The Casparian strip is required for ion homeostasis (e.g. iron and potassium ions).
Lma21013.t1	ZAT3_ARATH	Mediates the regulation of male germ cell division by DUO1.
Lma22207.t1	KN7B_ARATH	Probable plus end-directed motor protein that functions in the NACK-PQR (ANP3-MKK6-MPK4) MAP kinase signaling pathway, which is essential for somatic cell cytokinesis, especially for the cell-plate formation and its expansion. May regulate the activity and the localization of ANP3, probably by association through the non-catalytic region of the kinase. Functionally redundant with NACK1 and essential to promote the progression of cytokinesis and for cellularization (formation of the cell plate) during microgametogenesis and megagametogenesis.
Lma22627.t1	PS1_ARATH	Required for normal spindle orientation at male meiosis II and normal formation of tetrad of microspores. Not involved in female meiosis.
Lma20922.t1	C81F1_ARATH	Involved in indole glucosinolate biosynthesis. Catalyzes hydroxylation reactions of the glucosinolate indole ring. Converts indol-3-yl-methylglucosinolate (I3M) to 4-hydroxy-indol-3-yl- methylglucosinolate (4OH-I3M) and/or 1-hydroxy-indol-3-yl- methylglucosinolate (1OH-I3M) intermediates. These hydroxy intermediates are converted to 4-methoxy-indol-3-yl- methylglucosinolate (4MO-I3M) and 1-methoxy-indol-3-yl- methylglucosinolate (1MO-I3M) by indole glucosinolate methyltransferase 1 and 2 (IGMT1 and IGMT2).
Lma22165.t1	TCPB_ARATH	Molecular chaperone; assists the folding of proteins upon ATP hydrolysis. Known to play a role, in vitro, in the folding of actin and tubulin.
Lma21051.t1	SFH1_ARATH	Required for transport of secretory proteins from the Golgi complex (By similarity). Catalyzes the transfer of phosphatidylinositol and phosphatidylcholine between membranes in vitro. Plays a role in root hair tip elongation as a key regulator of polarized membrane trafficking. May promote the PtdIns(4,5)P2 synthesis and organization in root hair membrane.

Lma21958.t1	RPS5_ARATH	Disease resistance (R) protein that specifically recognizes the avrPphB type III effector avirulence protein from Pseudomonas syringae. Also confers resistance against Hyaloperonospora parasitica (downy mildew). Resistance proteins guard the plant against pathogens that contain an appropriate avirulence protein via an indirect interaction with this avirulence protein. That triggers a defense system including the hypersensitive response, which restricts the pathogen growth. Requires PBS1 to trigger the defense reaction against avrPphB. In case of infection by Pseudomonas syringae, AvrPphB triggers RPS5-mediated defense mechanism via the cleavage of PBS1, suggesting that the cleavage of PBS1 could trigger an exchange of ADP for ATP, thereby activating RPS5. May function as a fine-tuned sensor of alterations in the structure of the effector target PBS1.
Lma22297.t1	WRKY9_ARATH	Transcription factor. Interacts specifically with the W box (5'-(T)TGAC[CT]-3'), a frequently occurring elicitor- responsive cis-acting element (By similarity).
Lma22814.t1	DEF1_RAPSA	Possesses antifungal activity sensitive to inorganic cations.
Lma22836.t1	AEE21_ARATH	May act as an acid--thiol ligase that activates carboxylic acids by forming acyl-CoAs.
Lma21836.t2	MYO8_ARATH	Myosin heavy chain that is required for the cell cycle- regulated transport of various organelles and proteins for their segregation. Functions by binding with its tail domain to receptor proteins on organelles and exerting force with its N-terminal motor domain against actin filaments, thereby transporting its cargo along polarized actin cables.
Lma21857.t1	SMR6_ARATH	Probable cyclin-dependent protein kinase (CDK) inhibitor that functions as a repressor of mitosis in the endoreduplication cell cycle (By similarity). May inhibit CDKA-1/CYCD complexes during S-phase, preventing the re-initiation of DNA replication
Lma21674.t1	PS13A_ARATH	Acts as a regulatory subunit of the 26S proteasome which is involved in the ATP-dependent degradation of ubiquitinated proteins.
Lma21351.t1	IP5P1_ARATH	Has phosphatase activity toward Ins(1,4,5)P3 and Ins(1,3,4,5)P4, but not toward Ins(1,4)P2, Ins(1)PSeems to be involved in the abscisic acid (ABA) signaling pathwayCould also be able to hydrolyze PtdIns(4,5)P2 and PtdIns(3,4,5)P3
Lma22716.t1	RBG5_ARATH	Possibly has a role in RNA transcription or processing during stress (By similarity). Binds RNAs and DNAs sequence with a preference to single-stranded nucleic acids. Displays strong affinity to poly(U) sequenceInvolved in C-to-U editing of mitochondrial RNA. Functions as major mitochondrial editing factor. Controls 44 percent of the mitochondrial editing sites

Lma21759.t1	PER65_ARATH	Removal of H(2)O(2), oxidation of toxic reductants, biosynthesis and degradation of lignin, suberization, auxin catabolism, response to environmental stresses such as wounding, pathogen attack and oxidative stress. These functions might be dependent on each isozyme/isoform in each plant tissue.
Lma22281.t1	GGLO4_ARATH	May be involved in the biosynthesis of ascorbic acid.
Lma21757.t1	PMI12_ARATH	Pectin methylesterase (PME) inhibitor involved in the maintenance of cell wall integrity in response to necrotrophic pathogens. Modulates PME activity and pectin methylesterification during infection by Botrytis cinerea and contributes to resistance against the pathogen.
Lma22757.t2	IF4G_ARATH	Component of the protein complex eIF4F, which is involved in the recognition of the mRNA cap, ATP-dependent unwinding of 5'-terminal secondary structure and recruitment of mRNA to the ribosome. Plays a role in the accumulation of some potyvirus during viral infection. Required for the accumulation of cucumber mosaic virus 3a protein and turnip crinkle virus p28 replication protein during viral infection. These proteins are necessary for cell-to-cell movement of the virus.
Lma22404.t1	FH8_ARATH	Might be involved in the organization and polarity of the actin cytoskeleton. Interacts with the barbed end of actin filaments and nucleates actin-filament polymerization in vitro.
Lma21084.t1	AI5L6_ARATH	Binds to the ABA-responsive element (ABRE). Mediates stress-responsive ABA signaling.
Lma22039.t1	SOT18_ARATH	Sulfotransferase that utilizes 3'-phospho-5'-adenylyl sulfate (PAPS) as sulfonate donor to catalyze the sulfate conjugation of desulfo-glucosinolates (dsGSs), the final step in the biosynthesis of the glucosinolate core structure. Preferred substrate are the long-chain desulfo-glucosinolates, 7-methylthioheptyl and 8-methylthiooctyl, derived from methionine. Substrate preference is desulfo-benzyl glucosinolate > desulfo-4- methylthiobutyl glucosinolate > desulfo-6-methylthiohexyl glucosinolate > desulfo-3-methylthiopropyl glucosinolate > desulfo-indol-3-yl methyl glucosinolate > desulfo-singrin > desulfo-3-butenyl glucosinolate.
Lma22644.t1	FK173_ARATH	PPIases accelerate the folding of proteins. It catalyzes the cis-trans isomerization of proline imidic peptide bonds in oligopeptides (By similarity).
Lma22044.t1	AGD2_ARATH	Probable GTPase-activating protein.
Lma21919.t1	HMA5_ARATH	Involved in copper import into the cell. May play a role in copper detoxification in roots.

Lma15079.t1	ORTH2_ARATH	E3 ubiquitin-protein ligase. Participates in CpG methylation-dependent transcriptional regulation and epigenetic transcriptional silencing. Mediates ubiquitination with the E2 ubiquitin-conjugating enzyme UBC11. Promotes methylation-mediated gene silencing leading, for example, to early flowering. Associates with methylated DNA, and can bind to CpG, CpNpG, and CpNpN DNA motifs, with a strong preference for methylated forms, and with highest affinity for CpG substrate. Probably acts at the DNA methylation?histone interface to maintain centromeric heterochromatin.
Lma15970.t1	PSD7B_ARATH	Acts as a regulatory subunit of the 26S proteasome which is involved in the ATP-dependent degradation of ubiquitinated proteins.
Lma15698.t1	LEA76_BRANA	Lea proteins are late embryonic proteins abundant in higher plant seed embryos.
Lma15975.t1	FAD3C_ARATH	Chloroplast omega-3 fatty acid desaturase introduces the third double bond in the biosynthesis of 16:3 and 18:3 fatty acids, important constituents of plant membranes. It is thought to use ferredoxin as an electron donor and to act on fatty acids esterified to galactolipids, sulfolipids and phosphatidylglycerol.
Lma15068.t1	RVE1_ARATH	Morning-phased transcription factor integrating the circadian clock and auxin pathways. Binds to the evening element (EE) of promoters. Does not act within the central clock, but regulates free auxin levels in a time-of-day specific manner. Positively regulates the expression of YUC8 during the day, but has no effect during the night. Negative regulator of freezing tolerance.
Lma14597.t1	GCL2_ARATH	May play a role in signaling. May be not involved in abscisic acid (ABA) signaling.
Lma15128.t1	C82G1_ARATH	Catalyzes the conversion of the C20 (E,E)- geranyllinalool to the volatile C16-homoterpene 4,8,12-trimethyltrideca-1,3,7,11-tetraene (TMTT) that is produced upon insect herbivore attack. Catalyzes the conversion of the C15 (E)- nerolidol to the volatile C11-homoterpene (E)-4,8-dimethyl-1,3,7- nonatriene (DMNT).
Lma14836.t1	SBT17_ARATH	Serine protease. Has a substrate preference for the hydrophobic residues Phe and Ala and the basic residue Asp in the P1 position, and for Asp, Leu or Ala in the P1' positionEssential for mucilage release from seed coats. Triggers the accumulation and/or activation of cell wall modifying enzymes necessary either for the loosening of the outer primary cell wall, or to facilitate swelling of the mucilage
Lma14691.t1	RPOT3_ARATH	Nuclear-encoded DNA-dependent RNA polymerase that catalyzes the transcription of DNA into RNA in chloroplasts using the four ribonucleoside triphosphates as substrates (Probable). Required for chloroplast development and leaf mesophyll cell proliferation
Lma15056.t1	SR34_ARATH	General splicing factor. Can promote splice site selection in vitro presumably by antagonizing the effects of the A1 heterogeneous nuclear ribonucleoprotein. May have an essential function during early plant development.

Lma15410.t1	GRP5_ARATH	Involved in organ growth by promoting cell elongation processes.
Lma14793.t1	AAE2_ARATH	May act as an acid--thiol ligase that activates carboxylic acids by forming acyl-CoAs.
Lma15788.t2	AT12B_ARATH	Ubiquitin-like protein involved in cytoplasm to vacuole transport (Cvt) and autophagy vesicles formation. Conjugation with ATG5 through a ubiquitin-like conjugating system involving also ATG7 as an E1-like activating enzyme and ATG10 as an E2-like conjugating enzyme, is essential for its function. ATG12/ATG5 conjugate has an essential role in plant nutrient recycling.
Lma15837.t4	HMG10_ARATH	Binds preferentially DNA with A/T-rich content.
Lma16268.t1	HIP43_ARATH	Heavy-metal-binding protein.
Lma16079.t1	PAE5_ARATH	Hydrolyzes acetyl esters in homogalacturonan regions of pectin. In type I primary cell wall, galacturonic acid residues of pectin can be acetylated at the O-2 and O-3 positions. Decreasing the degree of acetylation of pectin gels in vitro alters their physical properties.
Lma14621.t1	RIC6_ARATH	Functions as downstream effector of Rho-related GTP binding proteins of the "Rho of Plants" (ROPs) family. Participates in the propagation of ROP GTPase signals in specific cellular responses. Is involved in pollen tube growth regulation through its interaction with ARAC11/ROP1.
Lma14716.t1	DSC2_ARATH	TIR-NB-LRR receptor-like protein involved in plant defense. Acts as a trigger of hypersensitive response (HR). Functions as guard of CAMTA3, a negative regulator of immunity, during pathogen infection.
Lma16309.t1	ATL6_ARATH	E3 ubiquitin-protein ligase able to catalyze polyubiquitination with ubiquitin-conjugating enzyme E2 UBC8 in vitro. May be involved in the plant C/N response and the early steps of the plant defense signaling pathway.
Lma15651.t1	ROSY1_ARATH	Involved in the regulation of gravitropic response and basipetal auxin transport in roots. Involved in salt stress tolerance. May facilitate membrane trafficking and asymmetric cell elongation via SYT1. Binds stigmasterol and dipalmitoyl phosphoethanolamine (DPPE) in vitro.
Lma16355.t1	LSH2_ARATH	Probable transcription regulator that acts as a developmental regulator by promoting cell growth in response to light.
Lma15787.t1	BIG1A_ARATH	Involved in auxin transport. Regulator of the auxin signaling pathway.
Lma15770.t1	GDL21_ARATH	Involved in organization of the endomembrane system and is required for endoplasmic reticulum morphology and organelle distribution. May act by inhibiting the formation of PYK10 complex by binding to GLL23 and exporting it from the ER. Required for proper subcellular localization of myrosinase TGG2. Has no lipase or esterase activity.
Lma15900.t1	LNK3_ARATH	Probable transcriptional coactivator.

Lma07210.t2	ARF1_SOLTU	GTP-binding protein involved in protein trafficking; may modulate vesicle budding and uncoating within the Golgi apparatus.
Lma08161.t1	CPC_ARATH	Transcription factor. Determines the fate of epidermal cell differentiation. Represses trichome development by lateral inhibition. Together with GL3 or BHLH2, promotes the formation of hair developing cells (H position) in root epidermis, probably by inhibiting non-hair cell formation. Represses the expression of GL2 and WER in H cells. Positively regulates stomatal formation in the hypocotyl
Lma07201.t1	NAC86_ARATH	Transcription factor directing sieve element enucleation and cytosol degradation. Not required for formation of lytic vacuoles. Regulates, with NAC045, the transcription of NEN1, NEN2, NEN3, NEN4, RTM1, RTM2, UBP16, PLDZETA, ABCB10 and At1g26450.
Lma06390.t1	ATL45_ARATH	Probable E3 ubiquitin-protein ligase that may possess E3 ubiquitin ligase activity in vitro (By similarity). May be involved in the early steps of the plant defense signaling pathway (Probable).
Lma08067.t1	IQD14_ARATH	May be involved in cooperative interactions with calmodulins or calmodulin-like proteins. May associate with nucleic acids and regulate gene expression at the transcriptional or post-transcriptional level (By similarity).
Lma07847.t1	FPGS3_ARATH	Catalyzes conversion of folates to polyglutamate derivatives allowing concentration of folate compounds in the cell and the intracellular retention of these cofactors, which are important substrates for most of the folate-dependent enzymes that are involved in one-carbon transfer reactions involved in purine, pyrimidine and amino acid synthesis. Essential for organellar and whole-plant folate homeostasis.
Lma07283.t1	RRT1_ARATH	Glycosyltransferase involved in the formation of rhamnogalacturonan I (RG-I) oligosaccharides in the seed coat mucilage, which is a specialized cell wall with abundant RG-ITransfers the rhamnose residue from UDP-beta-L- rhamnose to RG-I oligosaccharidesPrefers RG-I oligosaccharides with a degree of polymerization of 5 or larger than 5Does not act on oligosaccharides with a degree of polymerization of 4 or smaller than 4Does not require metal ions for its activity

Lma07362.t1	DRM2_ARATH	Involved in de novo DNA methylation. Controls asymmetric and CpNpG methylation. Required for FWA gene silencing but not for the maintenance of SUP gene silencing. Functionally redundant to CMT3 to maintain non-CpG methylation. Involved in RNA-directed DNA methylation (RdDM) (, ,). Acts as major DNA methyltransferase in the RdDM pathway, and is essential for RNA-directed de novo DNA methylation of cytosines in all sequence contexts (,). Associates with long non-coding RNA (lncRNA) produced by RNA polymerase V (Pol V). This association is dependent on AGO4 and IDN2, and results in DNA methylation of RdDM target loci
Lma07669.t1	HFA7A_ARATH	Transcriptional activator that specifically binds DNA sequence 5'-AGAAnnTTCT-3' known as heat shock promoter elements (HSE).
Lma06514.t1	PLRX4_ARATH	Modulates cell morphogenesis by regulating cell wall formation and assembly, and/or growth polarization.
Lma07290.t1	SP1L4_ARATH	Acts redundantly with SPR1 in maintaining the cortical microtubules organization essential for anisotropic cell growth.
Lma07060.t1	DRE1C_ARATH	Transcriptional activator that binds specifically to the DNA sequence 5'-[AG]CCGAC-3'. Binding to the C-repeat/DRE element mediates cold-inducible transcription. CBF/DREB1 factors play a key role in freezing tolerance and cold acclimation.
Lma07365.t1	AGP6_ARATH	Proteoglycan that seems to be implicated in diverse developmental roles such as differentiation, cell-cell recognition, embryogenesis and programmed cell death (By similarity). Plays an important role during the formation of the nexine layer of the pollen wall
Lma07583.t1	SBT17_ARATH	Serine protease. Has a substrate preference for the hydrophobic residues Phe and Ala and the basic residue Asp in the P1 position, and for Asp, Leu or Ala in the P1' positionEssential for mucilage release from seed coats. Triggers the accumulation and/or activation of cell wall modifying enzymes necessary either for the loosening of the outer primary cell wall, or to facilitate swelling of the mucilage
Lma06686.t1	GDU1_ARATH	Probable subunit of an amino acid transporter involved in the regulation of the amino acid metabolism. Stimulates amino acid export by activating nonselective amino acid facilitators. Required the interaction with the RING-type E3 ubiquitin-protein ligase LOG2 to fulfill its function. Plays a role in the Gln export at hydathodes, at xylem parenchyma into xylem sap and from mesophyll into leaf apoplasm. Acts upstream genes involved in the salicylic acid (SA) pathway and in the geminivirus-host interaction.
Lma06448.t1	DRL27_ARATH	Disease resistance protein.

Lma06684.t1	PER46_ARATH	Removal of H(2)O(2), oxidation of toxic reductants, biosynthesis and degradation of lignin, suberization, auxin catabolism, response to environmental stresses such as wounding, pathogen attack and oxidative stress. These functions might be dependent on each isozyme/isoform in each plant tissue.
Lma08130.t1	PX11E_ARATH	Involved in peroxisomal proliferation. Promotes peroxisomal duplication, aggregation or elongation without fission.
Lma25357.t1	MA652_ARATH	Microtubule-associated protein that stabilize microtubules (MT). Involved in the regulation of MT organization and dynamics. Confers MT resistance to the drug propyzamide and cold conditions.
Lma25396.t1	PIP25_ARATH	Aquaporins facilitate the transport of water and small neutral solutes across cell membranes.
Lma17212.t1	CAR1_ARATH	Stimulates the GTPase/ATPase activities of Obg-like ATPases (By similarity). Mediates the transient calcium-dependent interaction of PYR/PYL/RCAR abscisic acid (ABA) receptors with the plasma membrane and thus regulates ABA sensitivityBinds liposomes in the absence of exogenous Ca(2+), but this activity is enhanced in the presence of Ca(2+) and generates membrane curvature (By similarity).
Lma16857.t1	SPER2_ARATH	Adapter-like transcriptional repressor recruiting TPL/TPR corepressors to inhibit TCP transcription factors (By similarity). May be involved in leaf development.
Lma17499.t1	AB8I_ARATH	Involved in light signaling, probably by mediating the transport and correct distribution of protoporphyrin IX, a chlorophyll precursor, in response to far-red light.
Lma17788.t1	NHX5_ARATH	Involved in trafficking to the vacuole. Required for cell proliferation and cell expansion, but not for cell differentiation. Acts in low affinity electroneutral exchange of protons for cations such as Na(+) or K(+) across membranes. May also exchange Li(+) and Cs(+) with a lower affinity.
Lma17999.t1	ERF12_ARATH	Transcriptional activator involved in the regulation of plant development and tolerance to abiotic stressesInvolved in salt and osmotic stress response pathways. May be regulated by the stress-related genes RD29A, RD22, DREB1A or P5CS during stress responseBinds to the GCC-box pathogenesis-related promoter element. May be involved in the regulation of gene expression by stress factors and by components of stress signal transduction pathways (By similarity).
Lma17312.t1	SAU24_ARATH	Functions as positive effectors of cell expansion through modulation of auxin transport.
Lma17739.t1	SPT62_ARATH	Transcription elongation factor that enhances the transcription elongation by RNA polymerase II (RNAPII).
Lma18297.t1	ERF76_ARATH	Involved in the regulation of gene expression by stress factors and by components of stress signal transduction pathways. Transcription factor that binds to the GCC-box pathogenesis- related promoter element. Acts as a transcriptional inhibitor and may regulate other AtERFs (By similarity).

Lma18180.t1	PRL1_ARATH	Pleiotropic regulator of glucose, stress and hormone responses. Also regulates cytochrome P450 CYP90A1/CPD. Coordinates the expression of hormone- and stress-related genes and genes related to cell wall modification and growth, leading to altered sugar-dependent growth and developmental responses. Component of the MAC complex that probably regulates defense responses through transcriptional control and thereby is essential for plant innate immunity. By suppressing the expression of several (1)O(2)-responsive genes, PRL1 seems to play a major role in modulating responses of plants to environmental changes by interconnecting (1)O(2)-mediated retrograde signaling with other signaling pathways. Acts as negative regulator of SNF1-related protein kinases AKIN10 and AKIN11 via the inhibition of their interaction with SKP1/ASK1. Component of the CUL4-RBX1-DDB1-PRL1 E3 ubiquitin- protein ligase complex, PRL1 may function as the substrate recognition module within this complex, leading to the AKIN10 degradation.
Lma16544.t1	SH3P2_ARATH	Regulator for autophaosome formation and/or maturation (, Ref.9). Binds phosphatidylinositol 3-phosphate
Lma17230.t1	NLTP_VIGUN	Potential lipid transfer protein.
Lma17970.t1	EXTN3_ARATH	Structural component which strengthens the primary cell wallForms dendritic structures indicating a propensity for self-assembly through tyrosine cross-linking (,). Forms intermolecular cross- links exclusively by pulcherosine (three Tyr)Scaffold formation requires an unobstructed C-terminus of EXT3Required for the correct positioning of the cell plate during cytokinesis in cells of the developing embryoExtensins contain a characteristic repeat of the pentapeptide Ser-Pro(4). For this particular extensin, a typical repeat of Ser-Pro(3) is found
Lma18089.t1	TAR1_ARATH	Probably involved in auxin production. TAA1, TAR1 and TAR2 are required for proper embryo patterning.
Lma17676.t1	GAOX4_ARATH	Key oxidase enzyme in the biosynthesis of gibberellin that catalyzes the conversion of GA12 and GA53 to GA9 and GA20 respectively, via a three-step oxidation at C-20 of the GA skeleton.
Lma18026.t1	DEF02_ARATH	Confers broad-spectrum resistance to pathogens.
Lma16800.t1	KRP4_ARATH	Binds and inhibits CYCD2-1/CDKA-1 complex kinase activity. May target specifically CDKA-1.

Lma17479.t1	UBQ4_ARATH	Ubiquitin exists either covalently attached to another protein, or free (unanchored). When covalently bound, it is conjugated to target proteins via an isopeptide bond either as a monomer (monoubiquitin), a polymer linked via different Lys residues of the ubiquitin (polyubiquitin chains) or a linear polymer linked via the initiator Met of the ubiquitin (linear polyubiquitin chains). Polyubiquitin chains, when attached to a target protein, have different functions depending on the Lys residue of the ubiquitin that is linked: Lys-11-linked is involved in ERAD (endoplasmic reticulum-associated degradation) and in cell-cycle regulation; Lys-29-linked is involved in lysosomal degradation; Lys-33-linked is involved in kinase modification; Lys-48-linked is involved in protein degradation via the proteasome; Lys-63-linked is involved in endocytosis, and DNA- damage responses. Linear polymer chains formed via attachment by the initiator Met lead to cell signaling. Ubiquitin is usually conjugated to Lys residues of target proteins, however, in rare cases, conjugation to Cys or Ser residues has been observed. When polyubiquitin is free (unanchored-polyubiquitin), it also has distinct roles, such as in activation of protein kinases, and in signaling (By similarity).
Lma16932.t1	BZP16_ARATH	Transcriptional activator that binds to the G-box motif (5'-CACGTG-3') and other cis-acting elements with 5'-ACGT-3' core, such as Hex, C-box and as-1 motifs. Possesses high binding affinity to G-box, much lower affinity to Hex and C-box, and little affinity to as-1 elementG-box and G- box-like motifs are cis-acting elements defined in promoters of certain plant genes which are regulated by such diverse stimuli as light-induction or hormone control (Probable). Binds to the G-box motif 5'-CACGTG-3' of LHCB2.4 (At3g27690) promoter. May act as transcriptional repressor in light-regulated expression of LHCB2.4. Binds DNA as monomer. DNA-binding activity is redox- dependent
Lma17306.t1	DSC2_ARATH	TIR-NB-LRR receptor-like protein involved in plant defense. Acts as a trigger of hypersensitive response (HR). Functions as guard of CAMTA3, a negative regulator of immunity, during pathogen infection.
Lma16540.t1	VATL_GOSHI	Proton-conducting pore forming subunit of the membrane integral V0 complex of vacuolar ATPase. V-ATPase is responsible for acidifying a variety of intracellular compartments in eukaryotic cells.
Lma17789.t1	NHX5_ARATH	Involved in trafficking to the vacuole. Required for cell proliferation and cell expansion, but not for cell differentiation. Acts in low affinity electroneutral exchange of protons for cations such as Na(+) or K(+) across membranes. May also exchange Li(+) and Cs(+) with a lower affinity.

Lma18177.t1	AER_ARATH	Involved in the detoxification of reactive carbonyls (, ,). Acts on lipid peroxide-derived reactive aldehydesSpecific to a double bond activated by an adjacent carbonyl groupCan use both quinones and diamide as substrates, but not menadione, ferricyanide or phylloquinoneCan use 4-hydroxy-(2E)-nonenal (HNE), 4- hydroxy-(2E)-hexenal (HHE), (2E)-nonenal, (2E)-hexenal, (2E)- pentenal, propenal (acrolein), 3-buten-2-one and 3-penten-2-one, but not (R)-(-)-carvone, n-nonanal, n-hexanal, (3Z)-hexanal, cyclohex-2-en-1-one or 12-oxo phytodienoic acid (OPDA) as electron acceptorsCatalyzes the reduction of the alpha,beta-unsaturated bond of 2-alkenals, of lipid peroxide- derived oxenes 9-oxo-10(E),12(Z)-octadecadienoic acid (9-KODE) and 13-oxo-9(Z),11(E)-octadecadienoic acid (13-KODE), as well as 4-oxo-(2E)-nonenal and 4-hydroxynonenalCan use 12-oxo-10(E) dodecanoate (traumatin), trans-1,3 diphenyl-2- propenone, trans-1,4-diphenyl-2-butene-1,4-dione, 9-oxo-12,13- epoxy-(10E)-octadecenoic acid (trans-EKODE-1b) and 9,13-dihydroxy- 10-oxo-11-octadecenoic acid as substratesCatalyzes the reduction of the 7-8 double bond of phenylpropanal substrates, such as p-coumaryl aldehyde and coniferyl aldehyde (in vitro)Has activity towards toxic substrates, such as 4-hydroxy-(2E)-nonenal (in vitro)May play a distinct role in plant antioxidant defense and is possibly involved in NAD(P)/NAD(P)H homeostasis
Lma17664.t1	RS18_ARATH	Located at the top of the head of the 40S subunit, it contacts several helices of the 18S rRNA.
Lma18053.t1	GUN3_ARATH	May be involved in the sloughing (cell-cell separation) of the root cap cells from root tip.
Lma16980.t1	URH1_ARATH	Involved in pyrimidine breakdown rather than in pyrimidine salvage. Unable to use cytidine as a substrate.
Lma16764.t1	CYT2_ARATH	Specific inhibitor of cysteine proteinases. Probably involved in the regulation of endogenous processes and in defense against pests and pathogens (By similarity).
Lma18038.t1	RMR3_ARATH	Involved in the trafficking of vacuolar proteins. May function as a sorting receptor for protein trafficking to the protein storage vacuole (PSV) (By similarity).
Lma25382.t1	TRXB3_ARATH	Thioredoxin reductase (TR) that exhibits both TR and thioredoxin (Trx) activities. Contains a C-terminal functional Trx domain. Functions as an electron donor for plastidial 2-Cys peroxiredoxins and participates in a NADPH-dependent hydrogen peroxide scavenging system in chloroplasts in the dark. Required for chlorophyll biosynthesis and biogenesis of the photosynthetic apparatus. Activates aerobic cyclase which converts Mg- protoporhyrin monomethyl ester into protochlorophyllide. Involved in a light-dependent regulation of starch biosynthesis by redox activation of the ADP-glucose pyrophosphorylase (AGPase), a central enzyme of starch synthesis.
Lma12212.t1	EDL13_ARATH	Sugar transporter.

Lma11826.t1	SG1_ARATH	Required for the early stage of chloroplast development. May be involved in chloroplast protein biosynthesis and/or degradation.
Lma12004.t1	P2_ARATH	Catalyzes the reduction of the 7-8 double bond of phenylpropanal substrates, such as p-coumaryl aldehyde and coniferyl aldehyde (in vitro). Has activity towards toxic substrates, such as 4-hydroxy-(2E)-nonenal (in vitro) (By similarity). May play a distinct role in plant antioxidant defense and is possibly involved in NAD(P)/NAD(P)h homeostasis.
Lma10837.t2	PLDZ2_ARATH	Hydrolyzes glycerol-phospholipids at the terminal phosphodiesteric bond to generate phosphatidic acids (PA). Phosphatidylcholine-selectiveRegulates vesicle trafficking and auxin responsesRequired for the normal cycling of PIN-2 containing vesiclesContributes to the supply of inorganic phosphorus for cell metabolism and diacylglycerol moieties for galactolipid synthesis in phosphorus-starved roots (,). Involved in root elongation during phosphate limitation
Lma10915.t1	CATIN_ARATH	Required for embryogenesis. May be involved with the spliceosome.
Lma11167.t1	OHP2_ARATH	May play a photoprotective role within PSI in response to light stress.
Lma10991.t1	CALS3_ARATH	Involved in callose synthesis at the forming cell plate during cytokinesis. During plant growth and development, callose is found as a transitory component of the cell plate in dividing cells, is a major component of pollen mother cell walls and pollen tubes, and is found as a structural component of plasmodesmatal canals (By similarity).
Lma10934.t1	PLT3_ARATH	Plasma membrane sugar-proton symporter.
Lma11436.t1	AGP7_ARATH	Proteoglycan that seems to be implicated in diverse developmental roles such as differentiation, cell-cell recognition, embryogenesis and programmed cell death.
Lma10781.t3	DXS_ARATH	Catalyzes the acyloin condensation reaction between C atoms 2 and 3 of pyruvate and glyceraldehyde 3-phosphate to yield 1-deoxy-D-xylulose-5-phosphate (DXP). Is a limiting enzyme for plastidic isoprenoid biosynthesis and essential for chloroplast development.
Lma11781.t1	ZAT5_ARATH	Probable transcription factor that may be involved in stress responses.
Lma11896.t1	PBL8_ARATH	May be involved in plant defense signaling.
Lma12275.t1	PTR33_ARATH	Low-affinity nitrate transporter.
Lma11648.t1	GPAT7_ARATH	Esterifies acyl-group from acyl-ACP to the sn-1 position of glycerol-3-phosphate, an essential step in glycerolipid biosynthesis.
Lma12101.t1	DAW1_CHLRE	Functions as a cargo-specific adapter between intraflagellar transport (IFT) particles and outer row dynein. Required for efficient dynein-mediated transport into the flagellar compartment.

Lma11829.t1	TIC32_ARATH	Involved in protein precursor import into chloroplasts. Part of the redox regulon consisting of TIC32, TIC 55 and TIC62.
Lma11854.t1	ROSY1_ARATH	Involved in the regulation of gravitropic response and basipetal auxin transport in roots. Involved in salt stress tolerance. May facilitate membrane trafficking and asymmetric cell elongation via SYT1. Binds stigmasterol and dipalmitoyl phosphoethanolamine (DPPE) in vitro.
Lma10821.t1	CP20C_ARATH	PPIases accelerate the folding of proteins. It catalyzes the cis-trans isomerization of proline imidic peptide bonds in oligopeptides. Required for the light-induced increase of thiol accumulation. Assists the folding or assembly of SAT1 enzyme to form the cysteine synthase complex. Links light and redox signals to the regulation of cysteine biosynthesis in response to stress.
Lma11664.t1	LIMYB_ARATH	Transcriptional repressor that associates with ribosomal protein promoters.
Lma12155.t1	GL17_ARATH	May play a role in plant defense. Probably has no oxalate oxidase activity even if the active site is conserved.
Lma10939.t1	SPNS3_ARATH	Probable sphingolipid transporter.
Lma10870.t1	ADS3_ARATH	Fatty acid desaturase involved in the first desaturation step leading to the formation of hexadeca 7,10,13-trienoic acid (16:3(7Z,10Z,13Z)), the major functional components of thylakoid membranes (, ,). Required for chloroplast biogenesis at low temperatureAlso indirectly involved in the production of the oxylipin dinor-oxo-phyto-dienoic acid implicated in wound signaling
Lma01628.t1	RNS3_ARATH	May remobilize phosphate, particularly when cells senesce or when phosphate becomes limiting.
Lma00207.t1	ROGFB_ARATH	Guanine-nucleotide exchange factor (GEF) that acts as an activator of Rop (Rho of plants) GTPases by promoting the exchange of GDP for GTP. Functions as a light-signaling switch that functions in root growth and development through the activation of Rop in a phytochrome-dependent manner. May act as a negative regulator of phytochrome-mediated primary root development.
Lma00520.t1	PERK4_ARATH	Required during abscisic acid (ABA)-mediated activation of Ca(2+) channels. Regulates ABA signaling pathways. Modulates the expression of genes related to cell elongation and ABA signaling during root growth.
Lma00078.t1	NFYA5_ARATH	Stimulates the transcription of various genes by recognizing and binding to a CCAAT motif in promoters (By similarity). Involved in the blue light (BL) and abscisic acid (ABA) signaling pathways.

Lma00093.t1	UBIQP_HORVU	Ubiquitin exists either covalently attached to another protein, or free (unanchored). When covalently bound, it is conjugated to target proteins via an isopeptide bond either as a monomer (monoubiquitin), a polymer linked via different Lys residues of the ubiquitin (polyubiquitin chains) or a linear polymer linked via the initiator Met of the ubiquitin (linear polyubiquitin chains). Polyubiquitin chains, when attached to a target protein, have different functions depending on the Lys residue of the ubiquitin that is linked: Lys-6-linked may be involved in DNA repair; Lys-11-linked is involved in ERAD (endoplasmic reticulum-associated degradation) and in cell-cycle regulation; Lys-29-linked is involved in lysosomal degradation; Lys-33-linked is involved in kinase modification; Lys-48-linked is involved in protein degradation via the proteasome; Lys-63-linked is involved in endocytosis, DNA-damage responses as well as in signaling processes leading to activation of the transcription factor NF-kappa-B. Linear polymer chains formed via attachment by the initiator Met lead to cell signaling. Ubiquitin is usually conjugated to Lys residues of target proteins, however, in rare cases, conjugation to Cys or Ser residues has been observed. When polyubiquitin is free (unanchored-polyubiquitin), it also has distinct roles, such as in activation of protein kinases, and in signaling (By similarity).
Lma01110.t1	DEF2_SINAL	Inhibits bovine beta-trypsin and alpha-chymotrypsin on a 1:1 molar basis.
Lma00708.t1	IWS1_ARATH	Transcription factor involved in RNA polymerase II (RNAPII) transcription regulation. Involved in transcription elongation. May function at post-recruitment and elongation steps of transcription. May be recruited by BZR2/BES1 to target genes and promote their expression during transcription elongation process. Required for brassinosteroid (BR)-induced gene expressionRequired the for regulation of numerous nitrogen-responsive genes in roots. Acts in roots to repress NRT2.1 transcription in response to high nitrogen supply. This repression is associated with an IWS1-dependent increase of trimethylation on 'Lys-27' H3K27me3 at the NRT2.1 locus
Lma01493.t1	HAC12_ARATH	Acetyltransferase enzyme. Acetylates histones, giving a specific tag for transcriptional activation.
Lma02279.t1	P23A_ARATH	Acts as a co-chaperone for HSP90Controls root development through the modulation of auxin distribution in the root meristem
Lma00266.t1	SPP1_ARATH	Catalyzes the final step of sucrose synthesis.
Lma01326.t1	DHAR2_ARATH	Displays a dual function. As a soluble protein, exhibits glutathione-dependent thiol transferase and dehydroascorbate (DHA) reductase activitiesExhibits glutathione- dependent thiol transferase and dehydroascorbate (DHA) reductase activities. Key component of the ascorbate recycling system. Involved in the redox homeostasis, especially in scavenging of ROS under oxidative stresses. Plays a role in ozone tolerance.

Lma02073.t1	JASON_ARATH	Required for normal spindle orientation at male meiosis II and normal formation of tetrad of microspores. Acts as positive regulator of PS1 in male sporogenesis. Not involved in female meiosis.
Lma00981.t1	C86B1_ARATH	Involved in very long chain fatty acids (VLCFA) omega- hydroxylation. Required for the synthesis of saturated VLCFA alpha, omega-bifunctional suberin monomers.
Lma02103.t1	CML7_ARATH	Potential calcium sensor.
Lma00043.t1	BAM5_ARATH	Beta-amylase activity. Major cytosolic beta-amylase isoform in rosette leaves and inflorescences stems.
Lma01972.t1	RGAP5_ARATH	Acts as a GTPase activator for the Rac-type GTPase by converting it to an inactive GDP-bound state.
Lma00153.t1	GT14A_ARATH	Beta-glucuronosyltransferase involved in the biosynthesis of type II arabinogalactan (AG). Modifies both the beta-1,6-linked galactan and beta-1,3-linked galactan present in type II AG. Transfers glucuronate to beta-1,6- galactooligosaccharides with degrees of polymerization ranging from 3 to 11. Transfers glucuronate to beta-1,3- galactooligosaccharides with degrees of polymerization ranging from 5 to 7. The addition of glucuronate at the O6 position may terminate galactose chain extension. Required for cell elongation during seedling growth.
Lma01102.t1	T2FA_ARATH	TFIIF is a general transcription initiation factor that binds to RNA polymerase II and helps to recruit it to the initiation complex in collaboration with TFIIB. It promotes transcription elongation (By similarity).
Lma00175.t1	MPPA1_ARATH	Cleaves presequences (transit peptides) from mitochondrial protein precursors.
Lma01170.t1	PUM8_ARATH	Sequence-specific RNA-binding protein that regulates translation and mRNA stability by binding the 3'-UTR of target mRNAs.
Lma00058.t1	BRE1B_ARATH	E3 ubiquitin-protein ligase that monoubiquitinates H2B to form H2BK143ub1. H2BK143ub1 gives a specific tag for epigenetic transcriptional activation and is also prerequisite for H3K4me and maybe H3K79me. It thereby plays a central role in histone code and gene regulation. Forms a ubiquitin ligase complex in cooperation with the E2 enzyme UBC2/RAD6.
Lma00054.t1	DIR20_ARATH	Dirigent proteins impart stereoselectivity on the phenoxy radical-coupling reaction, yielding optically active lignans from two molecules of coniferyl alcohol in the biosynthesis of lignans, flavonolignans, and alkaloids and thus plays a central role in plant secondary metabolism.

Lma02086.t1	AGDP1_ARATH	Heterochromatin-binding protein that preferentially occupies long transposons and specifically recognizes the histone H3 'Lys-9' methylation (H3K9me) marks, whith a stronger affinity for dimethylated H3K9 (H3K9me2) (,). Required for transcriptional silencing, non-CG DNA methylation (e.g. CHG and CHH regions), and H3K9 dimethylation (H3K9me2) at some loci (,). Mediates heterochromatin phase separation and chromocenter formation
Lma00101.t1	PME2_ARATH	Acts in the modification of cell walls via demethylesterification of cell wall pectin.
Lma01225.t1	NSRB_ARATH	Alternative splicing (AS) regulator that binds to specific mRNAs and modulates auxin effects on the transcriptome. Displaced from its targets upon binding to AS competitor long non- coding RNA (ASCO-RNA).
Lma00832.t1	CIN2_ARATH	Involved in monoterpene (C10) biosynthesis. The major product is 1,8-cineole (52%) followed by minor amounts of sabinene (14.5%), myrcene (13.3%), (-)-(1S)-beta-pinene (7.8%), (-)-(4S)- limonene (4.0%), (E)-beta-ocimene (2.7%), alpha-terpineol (2.4%), (-)-(1S)-alpha-pinene (1.9%), terpinolene (0.8%), and (+)-alpha- thujene (0.6%).
Lma00451.t1	REHY_ARATH	Thiol-specific peroxidase that catalyzes the reduction of hydrogen peroxide and organic hydroperoxides to water and alcohols, respectively (By similarity). Seems to contribute to the inhibition of germination during stress
Lma00610.t2	IF413_TOBAC	ATP-dependent RNA helicase which is a subunit of the eIF4F complex involved in cap recognition and is required for mRNA binding to ribosome. In the current model of translation initiation, eIF4A unwinds RNA secondary structures in the 5'-UTR of mRNAs which is necessary to allow efficient binding of the small ribosomal subunit, and subsequent scanning for the initiator codon (By similarity).
Lma00809.t1	CSP1_ARATH	Chaperone that binds to RNA, single- (ssDNA) and double- stranded (dsDNA) DNA, and unwinds nucleic acid duplex. Exhibits a DNA melting activity. May be involved in cold resistance. Prevents seed germination under dehydration or salt stress conditions.
Lma00552.t1	PSBS_ARATH	Plays an important role in non-photochemical quenching, a process maintains the balance between dissipation and utilization of light energy to minimize generation of oxidizing molecules, thereby protecting the plant against photo-oxidative damage. Is not necessary for efficient light harvesting and photosynthesis.

Lma03645.t1	GSH1_ARATH	Seems to play an important role in controlling the expression of resistance responses like the regulation of salicylic acid (SA) and phytoalexin (camalexin) production. Involved in resistance to fungal and bacterial pathogens. Required for the regulation of cell proliferation in root apical meristems through the GSH-dependent developmental pathway. Also participates in the detoxification process, the antioxidant response and is essential for embryo development and proper seed maturation.
Lma03960.t1	PME20_ARATH	Acts in the modification of cell walls via demethylesterification of cell wall pectin.
Lma02547.t1	TPX2_ARATH	Regulates prospindle assembly during late prophase and at the onset of mitosis, before nuclear envelope breakdown (NEB). Is exported from the nucleus shortly before NEB and organized into two polar crescents. After NEB, is progressively associated with the forming spindle. Probably mediates AUR1 activation and localization to spindle microtubules. Has a microtubule binding capability and is able to trigger microtubule assembly induced by RanGTP in a heterologous system. Not involved in phragmoplast assembly, nuclear envelope reformation, and cortical microtubule assembly at the onset of G1Involved in the formation of specific nuclear and perinuclear microtubular arrays in the nuclei of acentrosomal plant cells. Fungi and plants have acentrosomal microtubule arrays because they lack centrosomes. They use other microtubule organizing center (MTOC) structures to organize their microtubules. May function through interaction with importin
Lma03828.t1	NHP2_ARATH	Required for ribosome biogenesis. Part of a complex which catalyzes pseudouridylation of rRNA. This involves the isomerization of uridine such that the ribose is subsequently attached to C5, instead of the normal N1. Pseudouridine ("psi") residues may serve to stabilize the conformation of rRNAs (By similarity).
Lma03102.t1	DGK6_ARATH	Phosphorylates the second messenger diacylglycerol (DAG) to generate phosphatidic acid (PA), another important signaling molecule. PA is required for plant development and responses to abiotic stress and pathogen attack. May be involved in the accumulation of PA during cold stress.
Lma03146.t1	SPL_ARATH	Transcriptional regulator of sporocyte developmentActs as an adapter-like transcriptional repressor recruiting TPL/TPR corepressors to inhibit TCP transcription factorsRequired for nucellus and embryo sac developmentPlays a central role in patterning both the proximal-distal and the adaxial-abaxial axes during ovule developmentInvolved in establishing the prospective chalaza of the ovule and in controlling the cell number and the length of the funiculus, and is required for the development of the integumentsRequired, with BEL1, for cytokinin-induced PIN1 expression in ovulesInvolved in controlling stamen identityMay also regulate the morphology of lateral organs by repressing auxin production

Lma03025.t1	AGP10_ARATH	Proteoglycan that seems to be implicated in diverse developmental roles such as differentiation, cell-cell recognition, embryogenesis and programmed cell death.
Lma02837.t1	SCL34_ARATH	Probable transcription factor involved in plant development.
Lma03216.t1	PUM6_ARATH	Sequence-specific RNA-binding protein that regulates translation and mRNA stability by binding the 3'-UTR of target mRNAs. Binds the APUM-binding elements (APBEs) in the 3'-UTR mRNA sequence of CLV1, PNH, WUS and FAS2.
Lma03112.t1	BOB2_ARATH	Small heat shock protein required for the establishment of auxin gradients and for patterning of the apical domain of the embryo. Involved in the specification of the cotyledon primordia. Also required for normal inflorescence and floral meristem function, normal developmental patterning and thermotolerance. Acts as a molecular chaperone (By similarity).
Lma03281.t1	OLEO3_BRANA	May have a structural role to stabilize the lipid body during desiccation of the seed by preventing coalescence of the oil. Probably interacts with both lipid and phospholipid moieties of lipid bodies. May also provide recognition signals for specific lipase anchorage in lipolysis during seedling growth.
Lma03350.t1	RHA4A_ARATH	Probable E3 ubiquitin-protein ligase that may possess E3 ubiquitin ligase activity in vitro.
Lma02404.t1	MGP4_ARATH	Catalyzes the transfer of D-xylose from UDP-alpha-D- xylose onto L-fucose. Probably involved in the biosynthesis of rhamnogalacturonan II (RG-II) through xylosylation of the internal fucose moiety of the A-chain of RG-II, a structurally complex pectic polysaccharide of the primary cell wall. RG-II is essential for the cell wall integrity of rapidly growing tissues such as roots and pollen tube growth and elongation.
Lma03144.t1	MYO2_ARATH	Myosin heavy chain that is required for the cell cycle- regulated transport of various organelles and proteins for their segregation. Functions by binding with its tail domain to receptor proteins on organelles and exerting force with its N-terminal motor domain against actin filaments, thereby transporting its cargo along polarized actin cables (By similarity). Involved in endocytosis via its action in endosomal trafficking.
Lma03902.t1	P5CS2_ARATH	P5CS plays a key role in proline biosynthesis, leading to osmoregulation in plants.
Lma03750.t1	MPS1_ARATH	Involved in the regulation of the onset of mitosis. Involved in a pathway that coordinates cell proliferation and differentiation. Implicated in spindle pole body (SPD) duplication (By similarity). May be a downstream regulator of auxin signaling in the formation of secondary roots (Probable).
Lma03175.t1	TI222_ARATH	Together with HP30-1 and HP30-2, triggers the import and insertion of transit sequence-less multi-pass transmembrane proteins (e.g. CEQORH) into the chloroplastic inner membrane.
Lma02634.t1	VA722_ARATH	Involved in the targeting and/or fusion of transport vesicles to their target membrane.

Lma03580.t1	CHLI1_ARATH	Involved in chlorophyll biosynthesis. Catalyzes the insertion of magnesium ion into protoporphyrin IX to yield Mg- protoporphyrin IX. The magnesium-chelatase is a complex of three subunits, CHLI, CHLD and CHLH. The reaction takes place in two steps, with an ATP-dependent activation followed by an ATP-dependent chelation step. Possesses high affinity for ATP and may play a major role in chlorophyll biosynthesis. Does not bind abscisic acid (ABA), but is a positive regulator of ABA signaling.
Lma24632.t1	AT18F_ARATH	The PI(3,5)P2 regulatory complex regulates both the synthesis and turnover of phosphatidylinositol 3,5-bisphosphate (PtdIns(3,5)P2). Required for autophagy (By similarity).
Lma24149.t1	AS1_ARATH	Transcription factor required for normal cell differentiation. Positively regulates LATERAL ORGAN BOUNDARIES (LOB) within the shoot apex, and the class III HD-ZIP genes REV, PHB, and PHV. Interacts directly with ASYMMETRIC LEAVES 2 (LBD6/AS2) to repress the knox homeobox genes BP/KNAT1, KNAT2, and KNAT6 and the abaxial determinants ARF3/ETT, KAN2 and YAB5. May act in parallel with the RDR6-SGS3-AGO7 pathway, an endogenous RNA silencing pathway, to regulate the leaf morphogenesis (, , , , , , , ,). Binds directly to KNAT1, KNAT2, and KNATM chromatin, regulating leaf developmentLBD6 is required for this bindingPositive regulator of flowering that binds to the promoter of FTRegulates FT expression by forming a functional complex with COInvolved in leaf polarity establishment by functioning cooperatively with NUCL1 to repress abaxial genes ARF3, ARF4, KAN1, KAN2, YAB1 and YAB5, and the knox homeobox genes KNAT1, KNAT2, KNAT6, and STM to promote adaxial development in leaf primordia at shoot apical meristems at high temperatures
Lma24092.t1	S40A1_ARATH	May be involved in iron transport and iron homeostasis.
Lma25242.t1	IPYR1_ARATH	Catalyzes the irreversible hydrolysis of pyrophosphate (PPi) to phosphate. The MgPPi(2-) complex binds to the enzyme only after a free Mg(2+) ion has bound (Ref.9). No activity with glycerol-3-phosphate, glucose-6-phosphate, p-nitrophenylphosphate, ADP, NADP(+), NAD(+),NADH, NADPH or phosphoribosyl pyrophosphate as substrates (Ref.9). Controls the equilibrium of gluconeogenic reactions in the heterotrophic growth phase of early seedling establishment. Determinates the rate of cytosolic glycolysis, providing carbon for seed storage lipid accumulation

Lma25196.t1	MD19B_ARATH	Component of the Mediator complex, a coactivator involved in the regulated transcription of nearly all RNA polymerase II-dependent genes. Mediator functions as a bridge to convey information from gene-specific regulatory proteins to the basal RNA polymerase II transcription machinery. The Mediator complex, having a compact conformation in its free form, is recruited to promoters by direct interactions with regulatory proteins and serves for the assembly of a functional preinitiation complex with RNA polymerase II and the general transcription factors (By similarity).
Lma23526.t1	ARFK_ARATH	Auxin response factors (ARFs) are transcriptional factors that bind specifically to the DNA sequence 5'-TGTCTC-3' found in the auxin-responsive promoter elements (AuxREs). Could act as transcriptional activator or repressor. Formation of heterodimers with Aux/IAA proteins may alter their ability to modulate early auxin response genes expression.
Lma24365.t1	CPSF2_ARATH	CPSF plays a key role in pre-mRNA 3'-end formation, recognizing the AAUAAA signal sequence and interacting with poly(A)polymerase and other factors to bring about cleavage and poly(A) addition (By similarity). Required for antisense-RNA- mediated gene silencing
Lma23710.t1	OEP37_ARATH	Voltage-dependent peptide-sensitive high conductance rectifying cation channel with a strong affinity for TIC32 that is imported into the chloroplast. Conductance is pH-dependent decreasing with decreasing pH values.
Lma23590.t1	SAP18_ARATH	Links the histone deacetylase complex to transcriptional repressors bound to chromatin. Involved in the tethering of the SIN3 complex to core histone proteins.
Lma23285.t1	GDU4_ARATH	Probable subunit of an amino acid transporter involved in the regulation of the amino acid metabolism. Stimulates amino acid export by activating nonselective amino acid facilitators.
Lma24466.t1	CP31B_ARATH	Required for specific RNA editing events in chloroplasts and stabilizes specific chloroplast mRNAs ().
Lma24309.t2	TRXO1_ARATH	Thiol-disulfide oxidoreductase that may participate in various redox reactions. Possesses insulin disulfide bonds reducing activity. Reduced by thioredoxin reductases NTRA and NTRB.
Lma24495.t2	MYB82_ARATH	Transcription activation factor positively regulating trichomes developmentHas a function nearly equivalent to that of GL1 and can complement gl1 mutants
Lma23840.t1	AGP23_ARATH	Proteoglycan that seems to be implicated in diverse developmental roles such as differentiation, cell-cell recognition, embryogenesis and programmed cell death.
Lma24311.t1	ATL31_ARATH	E3 ubiquitin-protein ligase that is required for the plant C/N response during seedling growth transition. May be involved in the early steps of the plant defense signaling pathway.
Lma24240.t1	LOG2_ARATH	Cytokinin-activating enzyme working in the direct activation pathway. Phosphoribohydrolase that converts inactive cytokinin nucleotides to the biologically active free-base forms.

Lma25212.t1	HIP33_ARATH	Heavy-metal-binding protein.
Lma23965.t1	WIH2_ARATH	Required for the promotion of megasporogenesis, or promotion of germ cell formation from somatic precursor cells. Acts redundantly with WIH1. Functions in a genetic pathway downstream of SPL/NZZ and WUS and together with TRN2 in promoting megasporogenesis.
Lma25087.t1	OFP10_ARATH	Transcriptional repressor that may regulate multiple aspects of plant growth and development through the regulation of BEL1-LIKE (BLH) and KNOX TALE (KNAT) homeodomain transcription factors.
Lma24680.t1	EDM2_ARATH	Cellular antisilencing factor and regulator of genome DNA methylation patterns involved in the regulation of chromatin states. Together with SUVH4, monitors repressive epigenetic marks H3K27me1, H3K9me2, and prevents DNA-methylation at CHG sites, affecting especially the expression of transposons and developmentally important genes (, ,). Regulates alternative RNA processing such as distal 3' polyadenylation by intronic heterochromatinTranscription factor that binds DNA and contributes to transcriptional transposable element (TE) silencing by modulating levels of the repressive post-translational histone modifications (PHM) H3K9me2In cv. Columbia, required for RPP7-dependent disease resistance against the Hyaloperonospora arabidopsidis isolate Hiks1, by promoting levels of RPP7 via alternative polyadenylation (APA), resulting from cooption of epigenetic information at the TE insertion locus COPIA-R7 (, ,). Regulates development processes such as the formation of leaf pavement cells, leaf expansion, fertility and flowering (, ,). Prevents FLC accumulation to control flowering
Lma24656.t1	ODO2A_ARATH	The 2-oxoglutarate dehydrogenase complex catalyzes the overall conversion of 2-oxoglutarate to succinyl-CoA and CO(2). It contains multiple copies of three enzymatic components: 2- oxoglutarate dehydrogenase (E1), dihydrolipoamide succinyltransferase (E2) and lipoamide dehydrogenase (E3) (By similarity).
Lma24673.t1	WAXS5_ARATH	Catalyzes the final step in the synthesis of long-chain linear esters (waxes).
Lma23269.t1	FLA3_ARATH	May be a cell surface adhesion protein.
Lma24605.t1	MYO2_ARATH	Myosin heavy chain that is required for the cell cycle- regulated transport of various organelles and proteins for their segregation. Functions by binding with its tail domain to receptor proteins on organelles and exerting force with its N-terminal motor domain against actin filaments, thereby transporting its cargo along polarized actin cables (By similarity). Involved in endocytosis via its action in endosomal trafficking.
Lma23900.t1	THAH_ARATH	Hydroxylates thalianol into thalian-diol.

Lma25128.t1	SAU76_ARATH	May be involved in the regulation of ethylene receptor signaling. Promotes cell expansion and plant growthInvolved in the regulation of cell elongation
Lma05011.t1	PP145_ARATH	Involved in RNA editing event in chloroplasts. Required for the editing of a single site in ndhD transcript, which is a plastid-encoded subunits of the chloroplast NAD(P)H dehydrogenase (NDH) complex. Not essential for the activity of the NDH complex of the photosynthetic electron transport chain.
Lma05246.t1	WDR5A_ARATH	Forms multiple COMPASS-like complexes involved in histone methylation by interacting with different histone H3 'Lys- 4' methyltransferases such as ATX1, SDG14 or SDG16Binds to target loci chromatin, increasing H3K4 trimethylation and causing activation of the geneUpregulates FLC and MAF4 expression to delay flowering
Lma04673.t1	Y1790_ARATH	May act as a substrate-specific adapter of an E3 ubiquitin-protein ligase complex (CUL3-RBX1-BTB) which mediates the ubiquitination and subsequent proteasomal degradation of target proteins.
Lma05836.t1	BZIP9_ARATH	Transcription factor.
Lma04332.t1	FLZ13_ARATH	May act as an adapter to facilitate the interaction of SnRK1 complex with effector proteins, conferring tissue- and stimulus-type specific differences in the SnRK1 regulation pathway.
Lma06079.t1	LNK1_ARATH	Transcriptional coactivator necessary for expression of the clock genes PRR5 and TOC1 (,). Antagonizes REV8 function in the regulation of anthocyanin accumulationInvolved in red light input to the clockActivates clock-controlled genes with afternoon peakMediates light inhibition of hypocotyl elongation
Lma04626.t1	HHO3_ARATH	Probable transcription factor involved in phosphate signaling in roots.
Lma05469.t1	GIL1_ARATH	Required for red (R) and far red (FR) light-induced and phytochrome-mediated deregulation of negative gravitropism leading to randomization of hypocotyl growth orientation.
Lma04996.t1	WHY3_ARATH	Single-stranded DNA-binding protein that functions in both chloroplasts and nucleus. In chloroplasts, maintains plastid genome stability by preventing break-induced and short homology- dependent illegitimate recombinations. In the nucleus, is recruited to a distal element upstream of the kinesin KP1 to mediate the transcriptional repression of KP1. Can bind double- stranded DNA in vivo.
Lma06179.t1	WIH1_ARATH	Required for the promotion of megasporogenesis, or promotion of germ cell formation from somatic precursor cells. Acts redundantly with WIH2. Functions in a genetic pathway downstream of SPL/NZZ and WUS and together with TRN2 in promoting megasporogenesis.

Lma05286.t1	VP52A_ARATH	Acts as component of the GARP complex that is involved in retrograde transport from early and late endosomes to the trans-Golgi network (TGN). The GARP complex facilitates tethering as well as SNARE complex assembly at the Golgi (By similarity). Required for pollen tube elongation and other polar growth.
Lma05202.t1	RPK1_ARATH	Involved in the main abscisic acid-mediated (ABA) signaling pathway and in early ABA perception. Together with RPK2, required for pattern formation along the radial axis (e.g. the apical embryonic domain cell types that generate cotyledon primordia), and the apical-basal axis (e.g. differentiation of the basal pole during early embryogenesis).
Lma06194.t1	PUB2_ARATH	Functions as an E3 ubiquitin ligase.
Lma04304.t1	BBX21_ARATH	Transcription activator that acts as positive regulator of seedling photomorphogenesisActs downstream of COP1 and play an important role in early and long-term adjustment of the shade avoidance syndrome (SAS) responses in natural environments
Lma06105.t1	WRK19_ARATH	Transcription factor. Interacts specifically with the W box (5'-(T)TGAC[CT]-3'), a frequently occurring elicitor- responsive cis-acting element. May act also as a disease resistance protein with a serine/threonine-protein kinase activity (By similarity).
Lma05310.t1	EMS1_ARATH	Receptor with a serine/threonine-protein kinase activity required for the specification of the correct number of male archesporial initials and for the subsequent specification of tapetal and middle cell layer identities. In seeds, required for enhancing cell size and the rate of embryonic development.
Lma06002.t2	SUD1_ARATH	Probable E3 ubiquitin ligase acting as a positive post- transcriptional regulator of 3-hydroxy-3-methylglutaryl-coenzyme A reductase activity. Might be involved in the quality control that degrades misfolded proteins (By similarity).
Lma05056.t1	KPYC_ARATH	Key regulatory enzyme of the glycolytic pathway that catalyzes the final step of glycolysis, converting ADP and phosphoenolpyruvate (PEP) to ATP and pyruvate by essentially irreversible transphosphorylation.
Lma06020.t1	PEX14_ARATH	Controls intracellular transport of both PTS1- and PTS2- containing proteins. Required for the proper targeting of PEX7 to the peroxisome.
Lma05279.t1	IMDH1_ARATH	Catalyzes the conversion of inosine 5'-phosphate (IMP) to xanthosine 5'-phosphate (XMP), the first committed and rate- limiting step in the de novo synthesis of guanine nucleotides, and therefore plays an important role in the regulation of cell growth.
Lma06087.t1	AGP1_ARATH	Proteoglycan that seems to be implicated in diverse developmental roles such as differentiation, cell-cell recognition, embryogenesis and programmed cell death.

Lma04147.t2	BRG2_ARATH	Probable E3 ubiquitin-protein ligase. Has no effect on the stability of the DELLA proteins.
Lma04261.t1	CST_ARATH	Acts as a spatial inhibitor of signaling that modulates abscission zone cell adhesion and expansion. Acts both directly and indirectly by physically interacting with RLK5/HAE and SOBIR1/EVR at the cell surface.
Lma04495.t1	PUB10_ARATH	Functions as an E3 ubiquitin ligase.
Lma05455.t1	PLCD4_ARATH	The production of the second messenger molecules diacylglycerol (DAG) and inositol 1,4,5-trisphosphate (IP3) is mediated by activated phosphatidylinositol-specific phospholipase C enzymes.
Lma04668.t1	CESA6_ARATH	Catalytic subunit of cellulose synthase terminal complexes ('rosettes'), required for beta-1,4-glucan microfibril crystallization, a major mechanism of the cell wall formation. Involved in the primary cell wall formation. The presence of each protein CESA1 and CESA6 is critical for cell expansion. The hypocotyl elongation is based on a CESA6-dependent cell elongation in dark and a CESA6-independent cell elongation in light. The transition between these two mechanisms requires photosynthesis and PHYB, but not CRY1. The CESA6-dependent cell elongation seems to be independent of gibberellic acid, auxin and ethylene. May be involved in sensitivity to isoxaben. Associates with and moves along cortical microtubules for the process of cellulose deposition.
Lma05314.t1	NAS2_ARATH	Synthesizes nicotianamine, a polyamine which serves as a sensor for the physiological iron status within the plant, and/or might be involved in the transport of iron.
